# A challenging redox neutral Cp*Co(III)-catalysed alkylation of acetanilides with 3-buten-2-one: synthesis and key insights into the mechanism through DFT calculations

**DOI:** 10.3762/bjoc.14.212

**Published:** 2018-09-10

**Authors:** Andrew Kenny, Alba Pisarello, Arron Bird, Paula G Chirila, Alex Hamilton, Christopher J Whiteoak

**Affiliations:** 1 Department of Biosciences and Chemistry, Sheffield Hallam University, Sheffield, S1 1WB, United Kingdom

**Keywords:** acetanilides, alkylation, C–H activation, cobalt catalysis, DFT studies

## Abstract

Traditional, established palladium cross-coupling procedures are widely applied in complex molecule synthesis; however, there is a significant disadvantage in the requirement for pre-functionalised substrates (commonly halides/triflates). Direct C–H activation protocols provide the opportunity for a novel approach to synthesis, although this field is still in its relative infancy and often transferability between substrate classes remains unresolved and limitations not fully understood. This study focuses on the translation of an established Cp*Co(III)-catalysed alkylation of benzamides to related acetanilides using 3-buten-2-one as coupling partner. The developed procedure provides a wide substrate scope in terms of substituted acetanilides, although the optimised conditions were found to be more forcing than those for the corresponding benzamide substrates. Interestingly, density functional theory (DFT) studies reveal that the major impediment in the mechanism is not the C–H activation step, but instead and unexpectedly, effective competition with more stable compounds (resting states) not involved in the catalytic cycle.

## Introduction

**Scheme 1:**
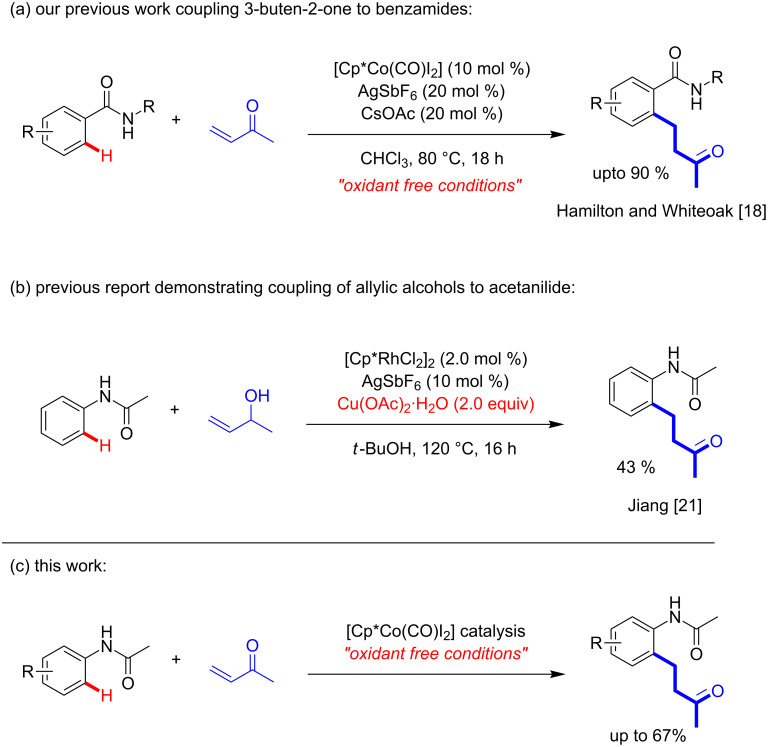
(a) Our previously reported Cp*Co(III) redox-neutral coupling of 3-buten-2-one to benzamides, (b) previous oxidative alkylation of acetanilide through the coupling of allylic alcohols under Cp*Rh(III) catalysis, and (c) the Cp*Co(III) redox-neutral coupling described in this work.

Controlled functionalisation of ubiquitous C–H bonds has been identified as one of the key challenges in modern day chemical research [1–3], providing the potential to access complex chemical structures more efficiently. In this context, transition metal catalysis is seen as a potential solution, building on the traditional and well-established palladium-catalysed cross-coupling protocols [4]. Whilst second and third row transition metals are well applied in cross-coupling protocols through C–H activation under mild conditions [5], the drive to use first row metals continues to provide an exciting challenge [6]. The interest in the application of these first-row transition metals stems from their low cost, ready availability and often wider reactivity profiles. One particular example which is currently attracting significant interest is cobalt, a metal which has found many applications in C–H functionalisation through exploitation of its diverse mechanisms [7]. Since 2013, the cobalt pre-catalysts, [Cp*Co(C_6_H_6_)](PF_6_)_2_ and [Cp*Co(CO)I_2_], have been successfully applied in a number of diverse C–H functionalisation protocols [8–12]. Whilst many of these protocols are very elegant, few examples are able to be applied to the full range of substrates and this presents one of the limitations to date compared with traditional palladium cross-coupling which is diversely applicable. Of interest to us are the readily available benzamide substrates, which are an interesting class of compounds as the amide moiety has been exploited as a common directing group [13] and countless pharmaceutical and agrochemical compounds contain these moieties. If the amide is reversed in the benzamide, the resulting compounds are acetanilides, which have been utilised far less as substrates in C–H functionalisation protocols [13], although a few examples do exist using the [Cp*Co(CO)I_2_] pre-catalyst [14–17]. Cp*Co(III)-catalysed C–H alkylation of unactivated aromatic C–H bonds with α,β-unsaturated ketones has been previously reported by ourselves (Scheme 1a) [18] and others [19–20]. Given our example focusing on the functionalisation of benzamides we wondered if the previously developed protocol could be directly transferred successfully to acetanilides, therefore further expanding the applicability of the developed methodology. The expected product from this reaction has previously been obtained through a C–H functionalisation approach in 43% yield from the Cp*Rh(III)-catalysed coupling of allylic alcohols with acetanilide through a redox-active mechanism (Scheme 1b) [21], thus requiring stoichiometric oxidant (Cu(OAc)_2_), whereas the new protocol described in this report is intended to provide a more attractive redox-neutral alternative, obviating the requirement for addition of terminal oxidant (Scheme 1c). Herein, our results from this study will be reported and the difficulties of this translation will be explained through a DFT study of the mechanism, which will also be directly compared with the use of benzamides as substrates.

## Results and Discussion

**Scheme 2:**
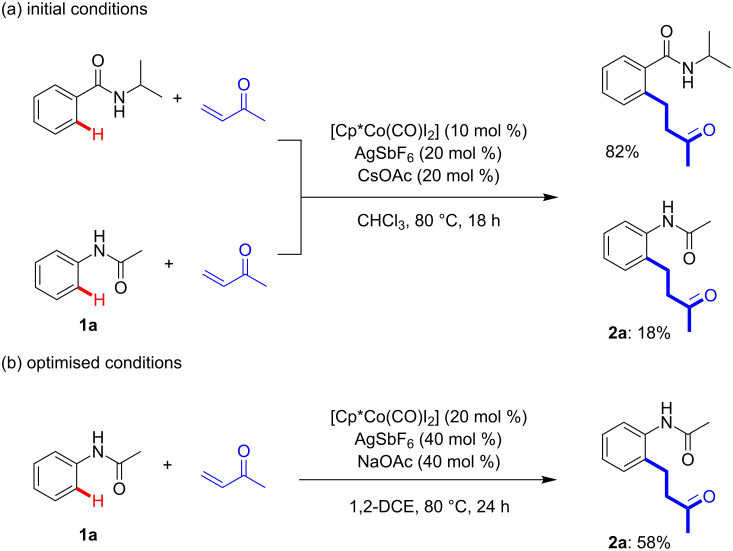
Summary of reaction conditions optimisation.

Initial investigations into the Cp*Co(III)-catalysed coupling of acetanilide (**1a**) with 3-buten-2-one, using the optimised conditions for the same coupling previously reported with benzamides, provided poor yields (18%; Scheme 2a). Subsequent reaction condition optimisation led to the inclusion of an increased catalyst loading (20 mol %) and change of solvent/base, which resulted in a synthetically useful yield of the coupling product **2a** (58%; Scheme 2b). This need for increased catalyst loading was also previously reported by Kanai and Matsunaga for the alkenylation of acetanilide with ethyl acrylate under Cp*Co(III) catalysis [14]. To the best of our knowledge, this is the first time that 3-buten-2-one has been successfully coupled to acetanilide through metal-mediated C–H functionalisation and provides a redox-neutral alternative, with enhanced yield, to the Cp*Rh(III)-catalysed coupling of allylic alcohols reported by Jiang and co-workers which requires the inclusion of 2.0 equivalents of Cu(OAc)_2_ for the same products [21].

**Scheme 3:**
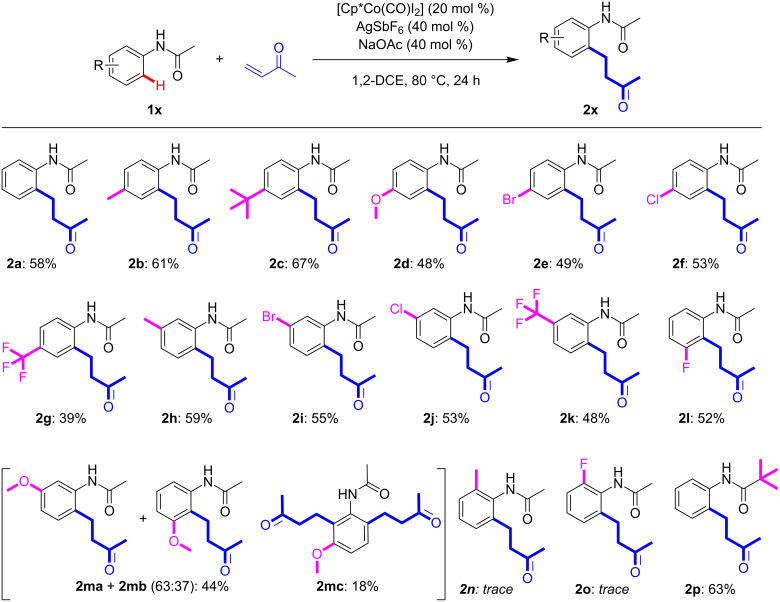
Substrate scope of Cp*Co(III)-catalysed coupling of 3-buten-2-one with functionalised acetanilides. All reactions carried out on a 1.0 mmol scale with isolated yields reported.

With the optimised conditions in hand, the potential scope/limitations of the catalytic protocol were studied (Scheme 3). Pleasingly, acetanilides with both electron-donating (**1b**–**d**) and electron-withdrawing substituents (**1e**–**g**) could be converted in yields of between 39-67%. The lower yields of some of these conversions highlight the challenging nature of this coupling. Thereafter, regioselectivity was studied by the inclusion of a range of *meta*-substituted acetanilides (**1h**–**m**). In most cases the products were obtained in a regioselective manner with substitution at the least hindered C–H bond. This regioselectivity has been observed previously in Cp*Co(III)-catalysis using benzamides as substrates by ourselves and others [14,18,22–24]. There are, however, two notable examples which should be commented upon; as we and others have previously observed, the *meta*-fluoro substituted compound favours functionalisation at the most hindered C–H bond, furnishing **2l**. Whilst the *meta*-methoxy-substituted acetanilide provided an unexpected inseparable mixture of the products derived from functionalisation of the least/most hindered C–H bond (**2ma** and **2mb**; combined yield of 44%) and a isolable amount (18%) of doubly functionalised product (functionalisation of least and most hindered C–H bonds), **2mc**. Neither acetanilides with either methyl or fluoro substituents in the *ortho*-position (**1n** and **1o**, respectively) could be successfully converted under the optimised conditions, with only traces of the products observed in the crude reaction mixtures. Increasing the steric bulk on the carbonyl from methyl to *tert*-butyl did not affect the obtained yield (**2p**).

**Figure 1:**
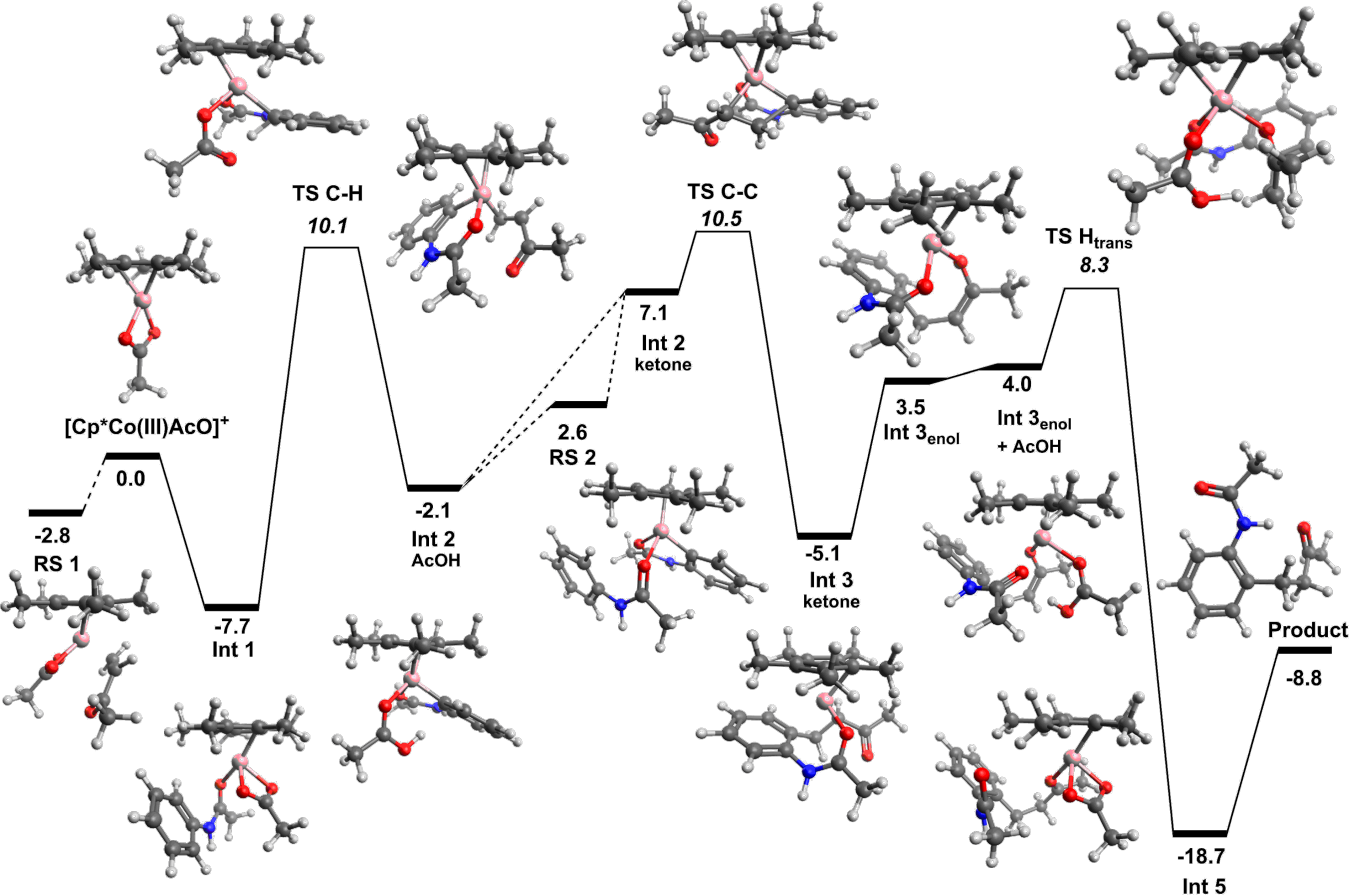
Mechanistic pathway for Cp*Co(III)-catalysed alkylation of acetanilide with 3-buten-2-one obtained from DFT studies; **Int A** is the direct interaction between the cationic [Cp*Co(III)AcO]^+^ species and the 3-buten-2-one coupling partner.

**Figure 2:**
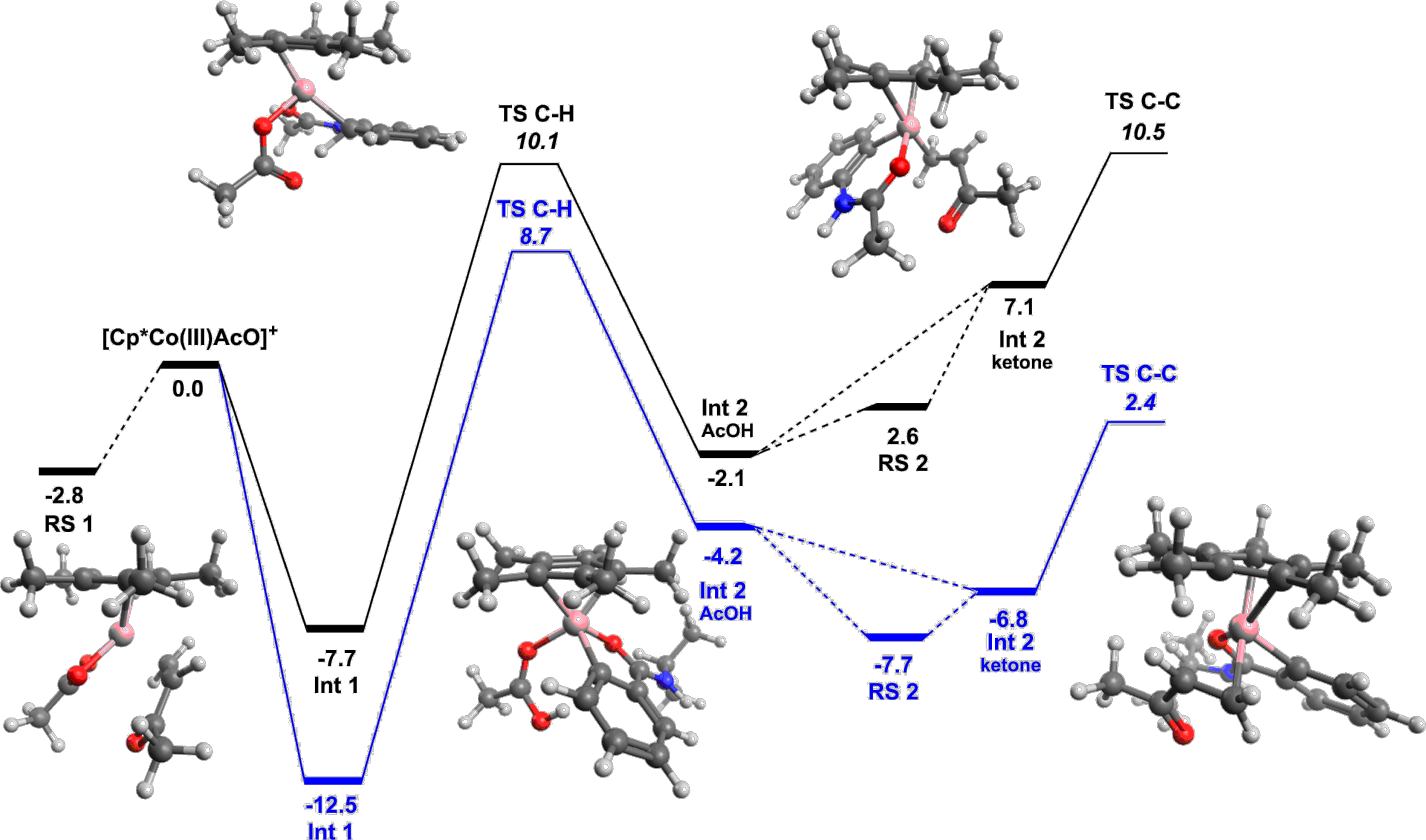
Comparison between energies during the Cp*Co(III)-catalysed coupling of 3-buten-2-one with acetanilide (black line) and benzamide (blue line); **RS 1** is the direct interaction between the cationic [Cp*Co(III)AcO]^+^ species and the 3-buten-2-one coupling partner and **RS 2** is the interaction of the metallocycle intermediate with a second acetanilide.

In an effort to further understand the reaction mechanism involved in the C–H functionalisation of acetanilide substrates with 3-buten-2-one, we employed DFT calculations (Figure 1) using M06 level of theory which has been previously successfully applied for cobalt-catalysed C–H functionalisation reactions [25–26]. Previous studies from our group have already discussed the O- vs N-binding of benzamide substrates to the [Cp*Co(III)OAc]^+^ catalyst [18]. In line with this benzamide functionalisation mechanism, the acetanilide coordinates to the cobalt centre through the ketone oxygen to form **Int 1**. This allows for reasonably close proximity of the C_sp_**_²_**-H proton for internal abstraction by the acetate group. The C–H activation step has an energy span barrier of 17.8 kcal mol^−1^, leading to the formation of the 6-membered organometallic cobaltacycle (**Int 2_AcOH_**) with an associated acetic acid. This barrier is approximately 3.5 kcal mol^−1^ lower in energy than the related benzamide C–H activation step, this in itself is an interesting result as it might logically be thought that C–H activation at the δ-position would be less favourable compared to the γ-position. Substitution of the acetic acid for 3-buten-2-one is energetically unfavourable (≈9 kcal mol^−1^), which differs significantly from the benzamide functionalisation example, where the substitution if favoured (Figure 2). The carbon–carbon bond formation step, functionalisation of the aromatic ring, proceeds with a low barrier (3.4 kcal mol^−1^) leading to an 8-membered cobaltacycle. As with the previous study the tautomerization to the metallo–enol structure is an important step in the reaction, interestingly the 8- to 10-membered ring tautomerization is energetically less hindered than the 7- to 9-membered benzamide equivalent. This energy difference could be influenced by the ordering of the reaction steps, with the addition of an acetic acid group to either the keto or enol form (benzamide or acetanilide respectively). Addition of the acetic acid group to the acetanilide keto intermediate (**Int 3_ketone_**) was calculated but proved to be less favourable than the initial tautomerization. Protonation of the unsaturated β-carbon position formed the highly stable **Int 5**, which dissociates to form the observed product and regenerate the cationic active catalyst species [Cp*Co(III)OAc]^+^. The less than 0.5 kcal mol^−1^ energy difference between the C–H activation and C–C bond formation steps makes identification of the rate limiting step difficult by DFT calculations alone, however, parallel kinetic isotope effect (KIE) experiments do suggest that the C–H activation step is not rate limiting (KIE = 1.3), which is not inconsistent with the calculated mechanism.

As demonstrated in this work, experimentally functionalisation of the acetanilide with 3-buten-2-one requires significantly harsher reaction conditions compared to the equivalent benzamide functionalisation. From initial comparison of the two free energy surfaces these results are difficult to interpret. Although the barriers for the acetanilide reaction are greater, no one barrier is significantly large enough to account for harsher conditions. One interesting difference between the two mechanisms is the different energy requirements for the addition of the ketone group and the 3-buten-2-one (Figure 2). The endergonic ligand exchange between acetic acid and ketone, for the acetanilide reaction, is clearly a differentiating step in the reaction. Coupled with a more energetically favourable resting state (**RS2**), resulting from addition of another substrate molecule to the initial metallocycle, the conversion is more challenging and therefore requires harsher reaction conditions. This competitive binding (**Int 2_substrate_** vs **RS 2**) is similar to that proposed by Bergman and Ellman for Cp*Rh(III)-catalysed arylation of imines [27]. Additionally **RS 1**, resulting from binding of the 3-buten-2-one to the active catalyst, for the acetanilide reaction is energetically more competitive compared to the benzamide reaction where both the ketone and substrate binding are preferable. The inclusion of a number of competitive intermediates/resting states on the potential energy surface goes some way to account for the observed differential experimental conditions for the two, different, yet related classes of substrate. This reactant limitation from **RS 1** is not observed in the benzamide reaction due to the exergonic nature of the ligand exchange (Figure 2). Although **RS 2** is energetically more favourable, compared to **Int 2_ketone_**, the energy difference of only 0.9 kcal mol^−1^ would lead to facile ligand exchange. Structurally the main difference between the acetanilide and benzamide intermediates is the 6- vs 5-membered cobaltacycle ring. Understanding the influence this difference has on the binding strength of the functionalising group (3-buten-2-one in this example) is an important step in understanding why some reactions catalysed by [Cp*Co(III)OAc]^+^ are more successful than others. To probe this phenomenon in more detail we performed quantum theory of atoms in molecules (QTAIM) analysis using Multiwfn software [28] of the two intermediate structures, identifying the relevant parameters at the bond critical points (*bcp*) of interest. QTAIM analysis has been used previously in the field of transition metal organometallic complexes to understand ligand binding [29–31].

**Figure 3:**
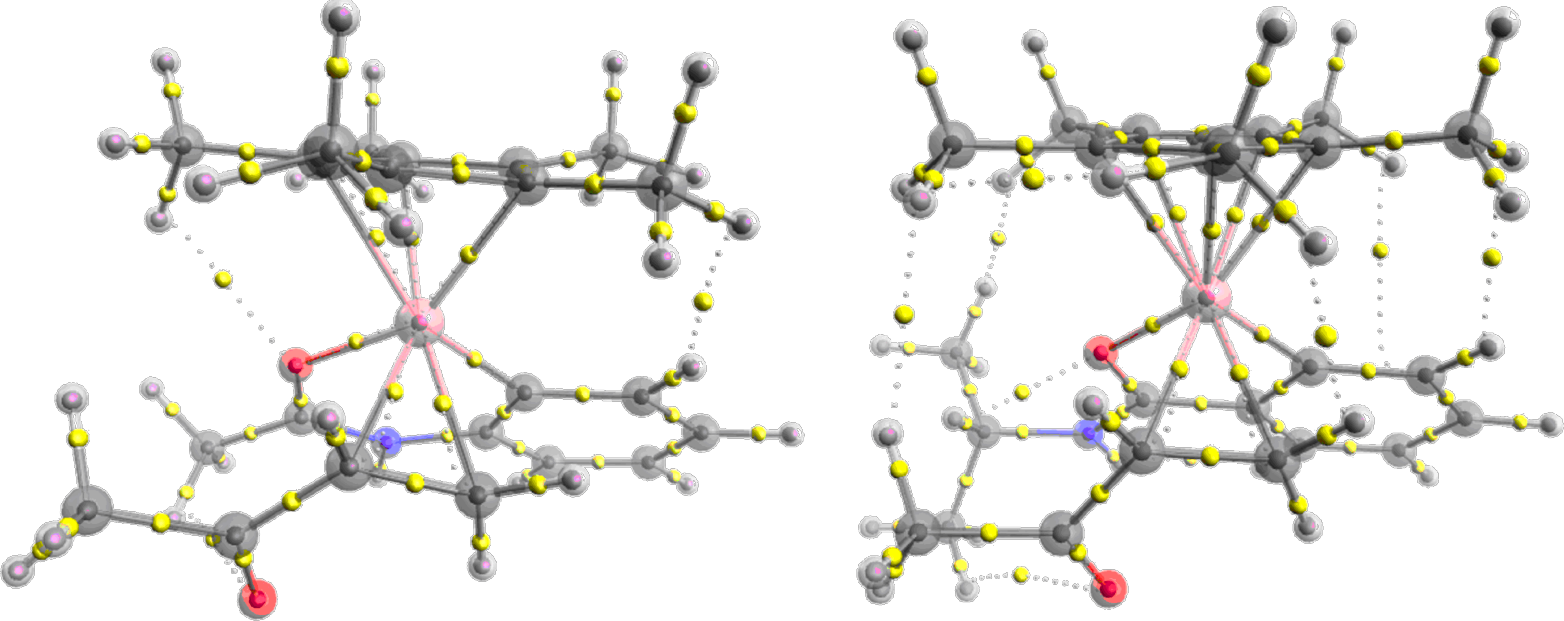
Comparative visualisation of *bcp* for **Int 2_ketone_** with the acetanilide (left) and benzamide substrates (right).

Analysis of the relative structural parameters for the two complexes (Table 1 and Figure 3) highlights an increase in bond lengths for the ketone substrate bound to the cobalt with the acetanilide ligand. The implied stronger cobalt to ketone interaction with the benzamide ligand is also confirmed with the QTAIM *bcp* parameters (Co·C_α_ and Co·C_β_); the increased electron density (ρ) and the greater negative terms for H(r) and V(r) all suggest a stronger bonding interaction. The decreased electron density at the C_α_·C_β_ *bcp* suggests greater donation of electron density to the cobalt, this is confirmed by the increase in electron density at the three centred *bcp* (Co·C_α_=C_β_). The slight asymmetric binding of the ketone is highlighted with shorter bond lengths and greater ρ and H(r) and V(r) parameters for Co·C_β_, this asymmetry is more pronounced for the acetanilide complex. The reason for the stronger binding of the ketone substrate to the Co-benzamide complex can be explained by the significant differences observed for the cobaltacycle ligand binding. The 5-membered cobaltacycle (with benzamide as the ligand) shows a significantly stronger cobalt–carbon interaction (Co·C_lig_) coupled with a decrease in the ionic nature of the Co·O interaction (positive 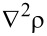 term) suggesting better orbital overlap for the 5-membered ring. The stronger binding to the benzamide ligand makes the cobalt centre more electron deficient, facilitating greater alkene π-electron donation and therefore a stronger interaction with the substrate. The combination of these two stabilising interactions reduces the relative energy of the benzamide complex with respect to the acetanilide complex.

**Table 1:** QTAIM and structural parameters for **Int 2_ketone_** with the acetanilide and benzamide substrates.

	QTAIM properties				

acetanilide	ρ	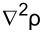	H(r)	V(r)	bond (Å)

Co·C_α_	0.0777	0.1924	−0.0225	−0.0931	2.13
Co·C_β_	0.0792	0.1851	−0.0241	−0.0945	2.10
C_α_·C_β_	0.3038	−0.8028	−0.3106	−0.4205	1.40
Co·C_α_=C_β_	0.0769	0.2423	−0.0199	−0.1003	2.00
Co·O	0.0859	0.4713	−0.0167	−0.1512	1.95
Co·C_lig_	0.1147	0.1385	−0.0504	−0.1355	1.97

benzamide	ρ	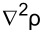	H(r)	V(r)	bond (Å)

Co·C_α_	0.0829	0.1950	−0.0261	−0.1001	2.09
Co·C_β_	0.0839	0.1906	−0.0271	−0.1018	2.08
C_α_·C_β_	0.3012	−0.7910	−0.3061	−0.4145	1.41
Co·C_α_=C_β_	0.0815	0.2600	−0.0221	−0.1092	1.96
Co·O	0.0853	0.4346	−0.0185	−0.1458	1.96
Co·C_lig_	0.1222	0.1489	−0.0565	−0.1502	1.94

**Scheme 4:**
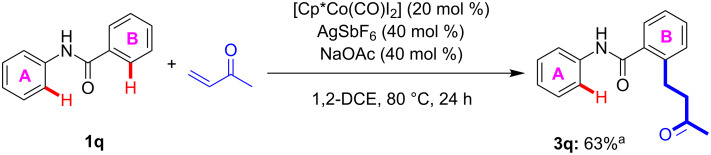
Competitive experiment between coupling to acetanilide (ring A) or benzamide (ring B). ^a^Major product **3q** obtained after purification with inseparable traces of proposed acetanilide coupling product.

In order to experimentally exemplify the preference in reactivity between the acetanilide and benzamide substrates, the acetanilide containing two aromatic moieties (**1q**) was subjected to the optimised reaction conditions (Scheme 4). The DFT studies suggested that selectivity should be observed between the two aromatic rings, in favour of the benzamide-type C–H functionalisation. In agreement with this proposal the reaction outcome demonstrates that the acetanilide environment is more challenging to convert than the corresponding benzamide environment. Indeed, the purified reaction product predominantly contains the benzamide substituted product **3q**, with traces of impurity which is proposed to be the acetanilide product (for the spectra see Supporting Information File 1). The exact regioselectivity of the major product was confirmed through the correlation between the carbonyl C atom and the single *ortho*-hydrogen atom on the newly substituted aromatic ring (see Supporting Information File 1 for all correlation spectra).

## Conclusion

In summary, the translation to acetanilides of a previously successful Cp*Co(III)-catalysed alkylation of benzamides with 3-buten-2-one has been attempted. It has been found that this reaction is extremely challenging under these original conditions and that in order to obtain synthetically useful yields a significant increase in catalyst loading (20 mol %) is required. The optimised protocol is able to successfully provide coupling products starting from a range of substituted acetanilides. The DFT studies on the mechanism demonstrate that in comparison to the previously reported benzamide example, the key step of co-ordination of the unsaturated coupling partner to the organometallic intermediate is significantly less favourable, thus a number of resting states of the catalyst become energetically more accessible, providing the reason for the requirement of more forcing conditions. Overall, this study provides an example of the challenges that need to be overcome when attempting to directly transfer an established protocol to even a related substrate class.

## Experimental

**Typical reaction protocol for alkylation:** The experimental alkylation procedure is similar to that as described in [18]. A screw top vial, under air, was charged with acetanilide substrate (1.0 mmol), [Cp*Co(CO)I_2_] (20 mol %, 0.20 mmol, 95.2 mg), AgSbF_6_ (40 mol %, 0.4 mmol, 137.4 mg), NaOAc (40 mol %, 0.4 mmol, 16.4 mg), 3-buten-2-one (1.5 equiv, 1.5 mmol, 105 mg) and 1,2-DCE (8.0 mL). The vial was sealed, and the reaction mixture heated to 80 °C with stirring for 24 hours. After this period, the solvent was removed under reduced pressure and the crude product purified by column chromatography (ethyl acetate/petroleum ether; 80:20 in most cases). For full characterisation data of all products obtained, see Supporting Information File 1.

**Computational details:** All DFT calculations undertaken using the ORCA 3.03 computational software [32]. Optimisations were performed at the BP86-D3BJ/def2-TZVP level of theory [33–39] and final single point energies and solvation corrections calculated at M06/def2-TZVP [38–41]. Frequencies calculations approximated the ZPE correction and entropic contributions to the free energy term as well as confirming all intermediate were true with no imaginary modes and all transition states had the correct critical frequency of decomposition (imaginary mode). Solvation correction was implemented with the COSMO [42] model for CH_2_Cl_2_. Graphical visualisation using Gabedit 2.4.8 [43] and Avogadro 1.2.0 [44] programs. For full computational details see Supporting Information File 1. QTAIM analysis was performed with Multiwfn software [28].

## Supporting Information

File 1 Experimental details and analytical data of new compounds including their original ^1^H and ^13^C and COSY spectra and data for all structures obtained from the DFT study.
