# Supplementary material for: A challenging redox neutral Cp*Co(III)-catalysed alkylation of acetanilides with 3-buten-2-one: synthesis and key insights into the mechanism through DFT calculations
Source: Beilstein J Org Chem. 2018 Sep 10;14:2366–74. doi: 10.3762/bjoc.14.212 (PMC6142744; doi:10.3762/bjoc.14.212)
Supplement: File 1 — Experimental details and analytical data of new compounds including their original 1H and 13C and COSY spectra and data for all structures obtained from the DFT study. [file Beilstein_J_Org_Chem-14-2366-s001.pdf]

Supporting Information

for

A challenging redox neutral Cp*Co(III)-catalyzed alkylation of acetanilides with 3-buten-2-one: synthesis and key insights into the mechanism through DFT calculations

Andrew Kenny, Alba Pisarello, Arron Bird, Paula G. Chirila, Alex Hamilton* and Christopher J. Whiteoak*

Address: Department of Biosciences and Chemistry, Sheffield Hallam University, Sheffield, S1 1WB, United Kingdom

Email: Christopher J. Whiteoak* - c.whiteoak@shu.ac.uk; Alex Hamilton* - a.hamilton@shu.ac.uk

*Corresponding author

Experimental details and analytical data of new compounds including their original ^{1}H and ^{13}C and COSY spectra and data for all structures obtained from the DFT study

Contents:

Page S2………………………. [1] General Experimental Considerations

Page S2………………………. [2] Optimized Procedure for Cp*Co(III)-Catalyzed Coupling of Acetanilides and 3-Buten-2-one

Page S2………………………. [3] Characterization Data for Aliphatic Ketone Products

Page S9…….................... [4] Original NMR Spectra for all Compounds

Page S34…………………. [5] Computational Details

Page S52…………………. [6] References

[1] General experimental considerations:

All solvents and reagents were purchased from Sigma-Aldrich, Fisher Scientific or Fluorochem and used without further purification. The [Cp*Co(CO)I_2] pre-catalyst was prepared according to the methodology of Kanai/Matsunaga.[1] ^{1}H, ^{13}C {^{1}H}, ^{19}F {^{1}H} and 2D NMR spectra were recorded on a Bruker AV-400 spectrometer in either $CDCl_3$ or D_6-DMSO purchased from Apollo Scientific. High-resolution mass spectra (HRMS) were recorded on a Xevo G2-Xs QTof Mass Spectrometer at Sheffield Hallam University.

[2] Optimized Procedure for Cp*Co(III)-Catalyzed Coupling of Acetanilides and 3-Buten-2-one:

This procedure is analogous to that described in our previous work.[2] A screw top vial, under air, was charged with acetanilide substrate (1.0 mmol), [Cp*Co(CO)I_2] (20 mol %, 0.20 mmol, 95.2 mg), $AgSbF_6$ (40 mol %, 0.4 mmol, 137.4 mg), NaOAc (40 mol %, 0.4 mmol, 16.4 mg), 3-buten-2-one (1.5 equiv, 1.5 mmol, 105 mg) and 1,2-DCE (8.0 mL). The vial was sealed, and the reaction mixture heated to 80 ^{o}C with stirring for 24 hours. After this period the solvent was removed under reduced pressure and the crude product purified by column chromatography (ethyl acetate/petroleum ether; 80:20 in most cases, unless otherwise stated). For full characterization data of all products obtained, see within this document.

[3] Characterization data for aliphatic ketone products:

N-(2-(3-Oxobutyl)phenyl)acetamide (**2a**)

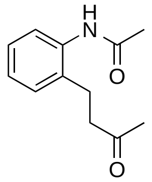

This compound was prepared by the general alkylation protocol described above starting from substrate **1a** (135 mg, 1.0 mmol) to yield a pale brown viscous oil (119 mg, 58%). ^{1}H NMR (D_6-DMSO, 400 MHz, 298 K); δ 9.33 (br s, 1H), 7.34 (d, 1H, $^3J_{HH}$ = 7.2 Hz), 7.21-7.05 (m, 3H), 2.79-2.64 (m, 4H), 2.10 (s, 3H), 2.05 (s, 3H). ^{13}C {^{1}H} NMR (D_6-DMSO, 100 MHz, 298 K); δ

= 207.9, 168.5, 135.9, 135.2, 129.1, 126.1, 126.0, 125.4, 42.9, 29.7, 24.7, 23.2. HR-MS (ASAP+, *m/z*); calcd. for $C_{12}H_{15}NO_2$+H = 206.1181; obtained = 206.1172 $[M+H]^+$. R_f = 0.54 (Ethyl acetate:Petroleum ether; 80:20).

N-(4-Methyl-2-(3-oxobutyl)phenyl)acetamide (**2b**)

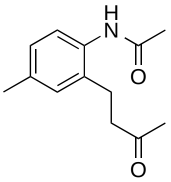

This compound was prepared by the general alkylation protocol described above starting from substrate **1b** (149 mg, 1.0 mmol) to yield a pale brown solid (133 mg, 61%). ^{1}H NMR (D_6-DMSO, 400 MHz, 298 K); δ 9.25 (br s, 1H), 7.18 (d, 1H, $^3J_{HH}$ = 7.9 Hz), 7.00 (s, 1H), 6.96 (d, 1H, $^3J_{HH}$ = 7.6 Hz), 2.71-2.65 (m, 4H), 2.24 (s, 3H), 2.09 (s, 3H), 2.02 (s, 3H). ^{13}C {^{1}H} NMR (D_6-DMSO, 100 MHz, 298 K); δ = 208.0, 168.4, 135.3, 134.5, 133.3, 129.6, 126.7, 126.1, 43.0, 29.6, 24.7, 23.1, 20.5. HR-MS (ASAP+, *m/z*); calcd. for $C_{13}H_{17}NO_2$+H = 220.1338; obtained = 220.1327 $[M+H]^+$. R_f = 0.58 (Ethyl acetate:Petroleum ether; 80:20).

N-(4-(*tert*-Butyl)-2-(3-oxobutyl)phenyl)acetamide (**2c**)

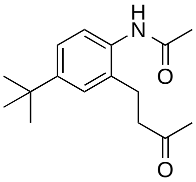

This compound was prepared by the general alkylation protocol described above starting from substrate **1c** (191 mg, 1.0 mmol) to yield a pale brown solid (175 mg, 67%). ^{1}H NMR (D_6-DMSO, 400 MHz, 298 K); δ 9.26 (br s, 1H), 7.24-7.13 (m, 3H), 2.76-2.65 (m, 4H), 2.09 (s, 3H), 2.03 (s, 3H), 1.26 (s, 9H). ^{13}C {^{1}H} NMR (D_6-DMSO, 100 MHz, 298 K); δ = 208.0, 168.5, 147.7, 134.8, 133.3, 125.9, 125.9, 43.2, 34.0, 31.1, 29.6, 25.2, 23.1. HR-MS (ASAP+, *m/z*); calcd. for $C_{16}H_{23}NO_2$+H = 262.1807; obtained = 262.1801 $[M+H]^+$. R_f = 0.51 (Ethyl acetate:Petroleum ether; 80:20).

N-(4-Methoxy-2-(3-oxobutyl)phenyl)acetamide (**2d**)

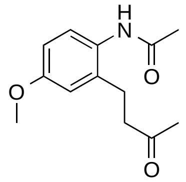

This compound was prepared by the general alkylation protocol described above starting from substrate **1d** (165 mg, 1.0 mmol) to yield a pale brown solid (112 mg, 48%). ^{1}H NMR (D_6-DMSO, 400 MHz, 298 K); δ 9.20 (br s, 1H), 7.14 (d, 1H, $^{3}J_{HH}$ = 8.0 Hz), 6.78-6.70 (m, 2H), (3.74 (s, 3H), 2.71-2.63 (m, 4H), 2.09 (s, 3H), 2.01 (s, 3H). ^{13}C {^{1}H} NMR (D_6-DMSO, 100 MHz, 298 K); δ = 207.9, 168.5, 156.9, 137.5, 128.8, 127.8, 114.3, 111.3, 55.1, 43.0, 29.6, 25.0, 23.0. HR-MS (ASAP+, *m/z*); calcd. for $C_{13}H_{17}NO_3$+H = 236.1287; obtained = 236.1278 $[M+H]^+$. R_f = 0.60 (Ethyl acetate:Petroleum ether; 80:20).

N-(4-Bromo-2-(3-oxobutyl)phenyl)acetamide (**2e**)

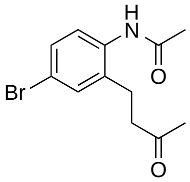

This compound was prepared by the general alkylation protocol described above starting from substrate **1e** (214 mg, 1.0 mmol) to yield a pale brown solid (139 mg, 49%). ^{1}H NMR (D_6-DMSO, 400 MHz, 298 K); δ 9.37 (br s, 1H), 7.42-7.38 (m, 1H), 7.36-7.32 (m, 2H), 2.75-2.69 (m, 4H), 2.10 (s, 3H), 2.05 (s, 3H). ^{13}C {^{1}H} NMR (D_6-DMSO, 100 MHz, 298 K); δ = 207.7, 168.6, 137.7, 135.4, 131.7, 128.9, 127.7, 117.5, 42.5, 29.6, 24.4, 23.2. HR-MS (ASAP+, *m/z*); calcd. for $C_{12}H_{14}NO_2Br$+H = 284.0286; obtained = 284.0277 $[M+H]^+$. R_f = 0.48 (Ethyl acetate:Petroleum ether; 80:20).

N-(4-Chloro-2-(3-oxobutyl)phenyl)acetamide (**2f**)

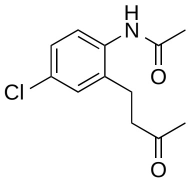

This compound was prepared by the general alkylation protocol described above starting from substrate **1f** (169 mg, 1.0 mmol) to yield a pale brown solid (127 mg, 53%). ^{1}H NMR

(D_6-DMSO, 400 MHz, 298 K); δ 9.38 (br s, 1H), 7.40 (d, 1H, $^3J_{HH}$ = 8.3 Hz), 7.27 (s, 1H), 7.22 (d, 1H, $^3J_{HH}$ = 8.3 Hz), 2.75-2.70 (m, 4H), 2.10 (s, 3H), 2.05 (s, 3H). ^{13}C {^1H} NMR (D_6-DMSO, 100 MHz, 298 K); δ = 207.7, 168.6, 137.4, 135.0, 129.1, 128.8, 127.4, 126.0, 42.5, 30.0, 24.5, 23.2. HR-MS (ASAP+, *m/z*); calcd. for $C_{12}H_{14}NO_2Cl$+H = 240.0791; obtained = 240.0781 $[M+H]^+$. R_f = 0.52 (Ethyl acetate:Petroleum ether; 80:20).

N-(2-(3-Oxobutyl)-4-(trifluoromethyl)phenyl)acetamide (**2g**)

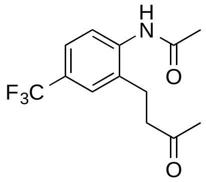

This compound was prepared by the general alkylation protocol described above starting from substrate **1g** (203 mg, 1.0 mmol) to yield a pale brown solid (106 mg, 39%). ^1H NMR (D_6-DMSO, 400 MHz, 298 K); δ 9.53 (br s, 1H), 7.73 (d, 1H, $^3J_{HH}$ = 8.9 Hz), 7.56 (s, 1H), 7.52 (d, 1H, $^3J_{HH}$ = 8.7 Hz), 2.87-2.80 (m, 2H), 2.80-2.73 (m, 2H), 2.11 (s, 6H). ^{19}F {^1H} NMR (D_6-DMSO, 376 MHz, 298 K); δ = 60.6. ^{13}C {^1H} NMR (D_6-DMSO, 100 MHz, 298 K); δ = 207.7, 168.8, 139.8, 135.0, 126.1 (q, $^3J_{CF}$ = 5.5 Hz), 125.3, 125.1 (q, $^2J_{CF}$ = 29.0 Hz), 124.3 (q, $^1J_{CF}$ = 271.0 Hz), 123.1 (q, $^3J_{CF}$ = 3.8 Hz) 42.4, 29.6, 24.4, 23.4. HR-MS (ASAP+, *m/z*); calcd. for $C_{13}H_{14}NO_2F_3$+H = 274.1055; obtained = 274.1046 $[M+H]^+$. R_f = 0.52 (Ethyl acetate:Petroleum ether; 80:20).

N-(5-Methyl-2-(3-oxobutyl)phenyl)acetamide (**2h**)

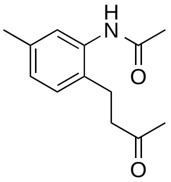

This compound was prepared by the general alkylation protocol described above starting from substrate **1h** (149 mg, 1.0 mmol) to yield a pale brown solid (129 mg, 59%). ^1H NMR (D_6-DMSO, 400 MHz, 298 K); δ 9.27 (br s, 1H), 7.15 (s, 1H), 7.06 (d, 1H, $^3J_{HH}$ = 7.7 Hz), 6.92 (d, 1H, $^3J_{HH}$ = 7.7 Hz), 2.73-2.64 (m, 4H), 2.24 (s, 3H), 2.09 (s, 3H), 2.04 (s, 3H). ^{13}C {^1H} NMR (D_6-DMSO, 100 MHz, 298 K); δ = 208.0, 168.4, 135.7, 135.1, 132.1, 129.0, 126.5, 126.1, 43.0, 30.0, 24.4, 23.2, 20.5. HR-MS (ASAP+, *m/z*); calcd. for $C_{13}H_{17}NO_2$+H = 220.1338; obtained = 220.1328 $[M+H]^+$. R_f = 0.53 (Ethyl acetate:Petroleum ether; 80:20).

N-(5-Bromo-2-(3-oxobutyl)phenyl)acetamide (**2i**)

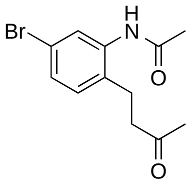

This compound was prepared by the general alkylation protocol described above starting from substrate **1i** (214 mg, 1.0 mmol) to yield a pale brown solid (156 mg, 55%). ^{1}H NMR (D_6-DMSO, 400 MHz, 298 K); δ 9.42 (br s, 1H), 7.66 (s, 1H), 7.28 (d, 1H, $^{3}J_{HH}$ = 7.4 Hz), 7.15 (d, 1H, $^{3}J_{HH}$ = 7.4 Hz), 2.76-2.66 (m, 4H), 2.10 (s, 3H), 2.07 (s, 3H). ^{13}C {^{1}H} NMR (D_6-DMSO, 100 MHz, 298 K); δ = 207.7, 168.7, 137.6, 133.8, 131.1, 127.7, 127.6, 118.3, 42.5, 30.0, 24.2, 23.3. HR-MS (ASAP+, *m/z*); calcd. for $C_{12}H_{14}NO_2Br$+H = 284.0286; obtained = 284.0279 $[M+H]^+$. R_f = 0.54 (Ethyl acetate:Petroleum ether; 80:20).

N-(5-Chloro-2-(3-oxobutyl)phenyl)acetamide (**2j**)

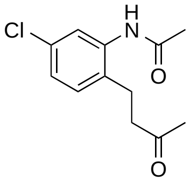

This compound was prepared by the general alkylation protocol described above starting from substrate **1j** (169 mg, 1.0 mmol) to yield a pale brown solid (127 mg, 53%). ^{1}H NMR (D_6-DMSO, 400 MHz, 298 K); δ 9.41 (br s, 1H), 7.54 (s, 1H), 7.22 (d, 1H, $^{3}J_{HH}$ = 7.8 Hz), 7.15 (d, 1H, $^{3}J_{HH}$ = 7.8 Hz), 2.78-2.65 (m, 4H), 2.10 (s, 3H), 2.08 (s, 3H). ^{13}C {^{1}H} NMR (D_6-DMSO, 100 MHz, 298 K); δ = 207.8, 168.7, 137.3, 133.3, 130.7, 130.0, 124.7, 124.7, 42.6, 29.7, 24.1, 23.3. HR-MS (ASAP+, *m/z*); calcd. for $C_{12}H_{14}NO_2Cl$+H = 240.0791; obtained = 240.0785 $[M+H]^+$. R_f = 0.51 (Ethyl acetate:Petroleum ether; 80:20).

N-(2-(3-Oxobutyl)-5-(trifluoromethyl)phenyl)acetamide (**2k**)

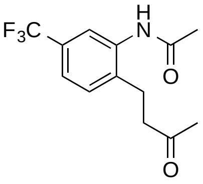

This compound was prepared by the general alkylation protocol described above starting from substrate **1k** (203 mg, 1.0 mmol) to yield a pale brown solid (131 mg, 48%). ^{1}H NMR

(D_6-DMSO, 400 MHz, 298 K); δ 9.53 (br s, 1H), 7.85-7.80 (s, 1H), 7.47-7.40 (m, 2H), 2.87-2.80 (m, 2H), 2.80-2.72 (m, 2H), 2.11 (s, 3H), 2.10 (s, 3H). ^{19}F {^{1}H} NMR (D_6-DMSO, 376 MHz, 298 K); δ = 60.9. ^{13}C {^{1}H} NMR (D_6-DMSO, 100 MHz, 298 K); δ = 207.6, 169.0, 139.1, 136.7, 130.2, 126.8 (q, $^2J_{CF}$ = 34.9 Hz), 123.9 (q $^1J_{CF}$ = 273.0 Hz), 121.5 (q, $^3J_{CF}$ = 4.1 Hz), 121.3 (q, $^3J_{CF}$ = 4.2 Hz) 42.2, 29.6, 24.5, 23.3. HR-MS (ASAP+, *m/z*); calcd. for $C_{13}H_{14}NO_2F_3$+H = 274.1055; obtained = 274.1048 $[M+H]^+$. R_f = 0.46 (Ethyl acetate:Petroleum ether; 80:20).

N-(3-Fluoro-2-(3-oxobutyl)phenyl)acetamide (**2l**)

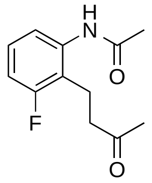

This compound was prepared by the general alkylation protocol described above starting from substrate **1l** (153 mg, 1.0 mmol) to yield a pale brown solid (115 mg, 52%). ^{1}H NMR (D_6-DMSO, 400 MHz, 298 K); δ 9.47 (br s, 1H), 7.30-7.14 (m, 2H), 7.03-7.6.94 (m, 1H), 2.81-2.70 (m, 2H), 2.70-2.61 (m, 2H), 2.10 (s, 3H), 2.07 (s, 3H). ^{19}F {^{1}H} NMR (D_6-DMSO, 376 MHz, 298 K); δ = 116.8. ^{13}C {^{1}H} NMR (D_6-DMSO, 100 MHz, 298 K); δ = 207.9, 168.7, 160.8 (d, $^1J_{CF}$ = 242.0 Hz), 137.7 (d $^3J_{CF}$ = 6.0 Hz), 127.1 (d, $^3J_{CF}$ = 7.7 Hz), 122.5 (d, $^2J_{CF}$ = 15.5 Hz), 121.7, 111.8 (d, $^2J_{CF}$ = 18.0 Hz), 42.2, 29.5, 23.2, 18.1 (d, $^3J_{CF}$ = 3.5 Hz). HR-MS (ASAP+, *m/z*); calcd. for $C_{12}H_{14}NO_2F$+H = 224.1087; obtained = 224.1079 $[M+H]^+$. R_f = 0.52 (Ethyl acetate:Petroleum ether; 80:20).

<u>**Mixture**</u> of *N*-(5-methoxy-2-(3-oxobutyl)phenyl)acetamide (**2ma**) and *N*-(3-methoxy-2-(3-oxobutyl)phenyl)acetamide (**2mb**)

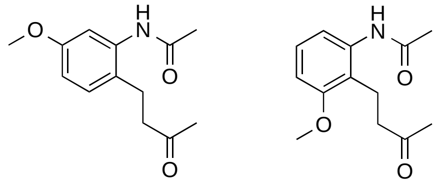

This compound mixture was prepared by the general alkylation protocol described above starting from substrate **1m** (165 mg, 1.0 mmol) to yield a combined pale brown solid (103 mg, 44%).For original spectra see later in this supporting information document. This mixture is consistent with the expected ^{1}H NMR spectra of the proposed compounds. HR-

MS (ASAP+, *m/z*); calcd. for $C_{13}H_{17}NO_3$+H = 236.1287; obtained = 236.1281 $[M+H]^+$. R_f = 0.53 (Ethyl acetate:Petroleum ether; 80:20).

N-(3-Methoxy-2,6-bis(3-oxobutyl)phenyl)acetamide (**2mc**)

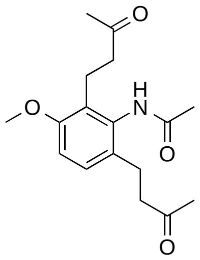

This compound was prepared by the general alkylation protocol described above starting from substrate **1m** (165 mg, 1.0 mmol) and obtained as an isolable side product from the intended synthesis of **2ma** as a pale brown solid (55 mg, 18%). DMSO, 400 MHz, 298 K); δ 9.28 (br s, 1H), 6.94 (m, 2H), 3.70 (s, 3H), 2.68-2.60 (m, 8H), 2.08 (s, 6H), 2.04 (s, 3H). ^{13}C {^1H} NMR (D_6-DMSO, 100 MHz, 298 K); δ = 208.2, 207.9, 168.5, 155.0, 134.7, 130.0, 126.5, 125.9, 108.5, 55.3, 43.4, 42.8, 29.6, 29.6, 24.1, 23.7, 23.2. HR-MS (ASAP+, *m/z*); calcd. for $C_{17}H_{23}NO_4$+H = 306.1705; obtained = 306.1695 $[M+H]^+$. R_f = 0.33 (Ethyl acetate:Petroleum ether; 80:20).

N-(2-(3-Oxobutyl)phenyl)pivalamide (**2p**)

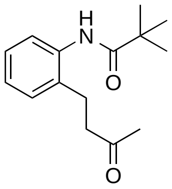

This compound was prepared by the general alkylation protocol described above starting from substrate **1p** (177 mg, 1.0 mmol) to yield a pale brown viscous oil (155 mg, 63%). ^1H NMR (D_6-DMSO, 400 MHz, 298 K); δ 9.03 (br s, 1H), 7.24-7.19 (m, 1H), 7.19-7.11 (m, 2H), 2.76-2.65 (m, 4H), 2.07 (s, 3H), 1.23 (s, 9H). ^{13}C {^1H} NMR (D_6-DMSO, 100 MHz, 298 K); δ = 208.3, 176.6, 137.0, 136.1, 129.2, 127.4, 126.1, 126.0, 43.3, 38.6, 29.6, 27.3, 24.7. HR-MS (ASAP+, *m/z*); calcd. for $C_{15}H_{21}NO_2$+H = 248.1651; obtained = 248.1641 $[M+H]^+$. R_f = 0.64 (Ethyl acetate:Petroleum ether; 20:80).

2-(3-Oxobutyl)-*N*-phenylbenzamide (**3q**)

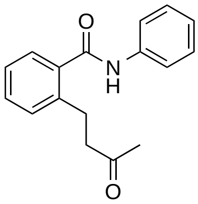

This compound was prepared by the general alkylation protocol described above starting from substrate **1q** (197 mg, 1.0 mmol) to yield a pale brown viscous oil which contains traces of another product which is proposed to be alkylation on the second aromatic ring, although is purely speculative (168 mg, 63%). ^{1}H NMR ($CDCl_3$, 400 MHz, 298 K); δ 8.82 (br s, 1H), 7.63 (d, 2H, $^{3}J_{HH}$ = 7.1 Hz), 7.46 (d, 1H, $^{3}J_{HH}$ = 7.1 Hz), 7.34-7.24 (m, 3H), 7.22-7.15 (m, 2H), 7.10-7.03 (m, 1H), 2.99-2.89 (m, 4H), 2.08 (s, 3H). ^{13}C {^{1}H} NMR ($CDCl_3$, 100 MHz, 298 K); δ = 209.4, 168.1, 138.4, 138.3, 137.2, 130.3, 130.2, 129.2, 129.1, 128.5, 128.0, 126.6, 124.4, 120.0, 44.7, 30.2, 26.4. HR-MS (ASAP+, *m/z*); calcd. for $C_{17}H_{16}NO_2$+H = 267.1259; obtained = 267.1254 $[M+H]^+$. R_f = 0.61 (Ethyl acetate:Petroleum ether; 20:80).

[4] Original NMR Spectra for all Compounds:

This section contains the original ^{1}H, ^{13}C {^{1}H}, ^{19}F {^{1}H} and COSY NMR spectra obtained for all alkylation products reported in this manuscript.

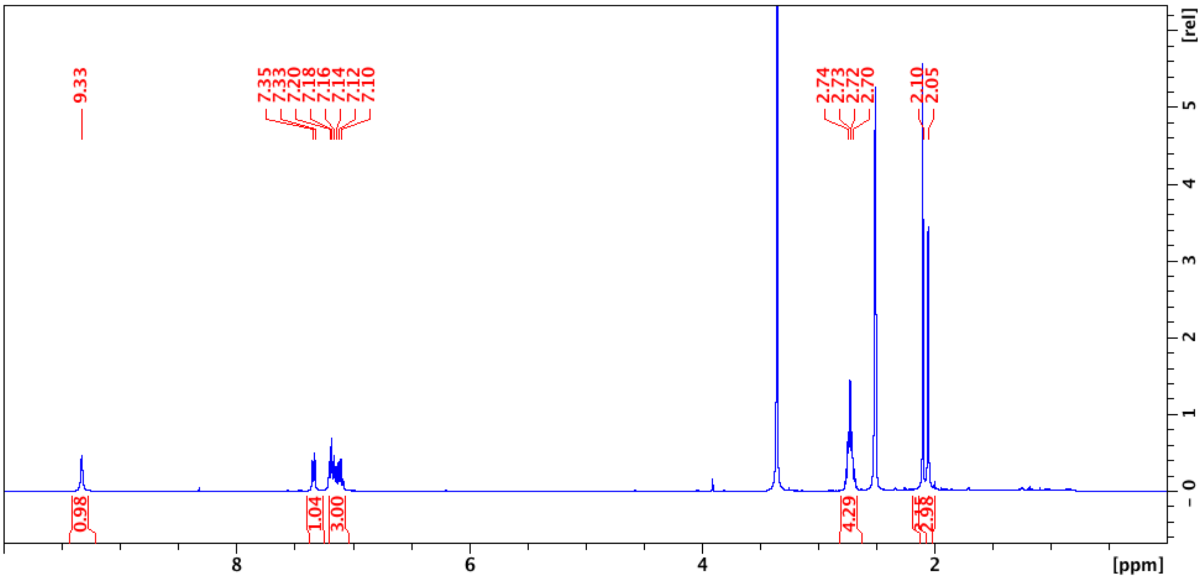

*Figure S1: ^{1}H NMR spectrum of compound **2a** in D_6-DMSO at 298 K.*

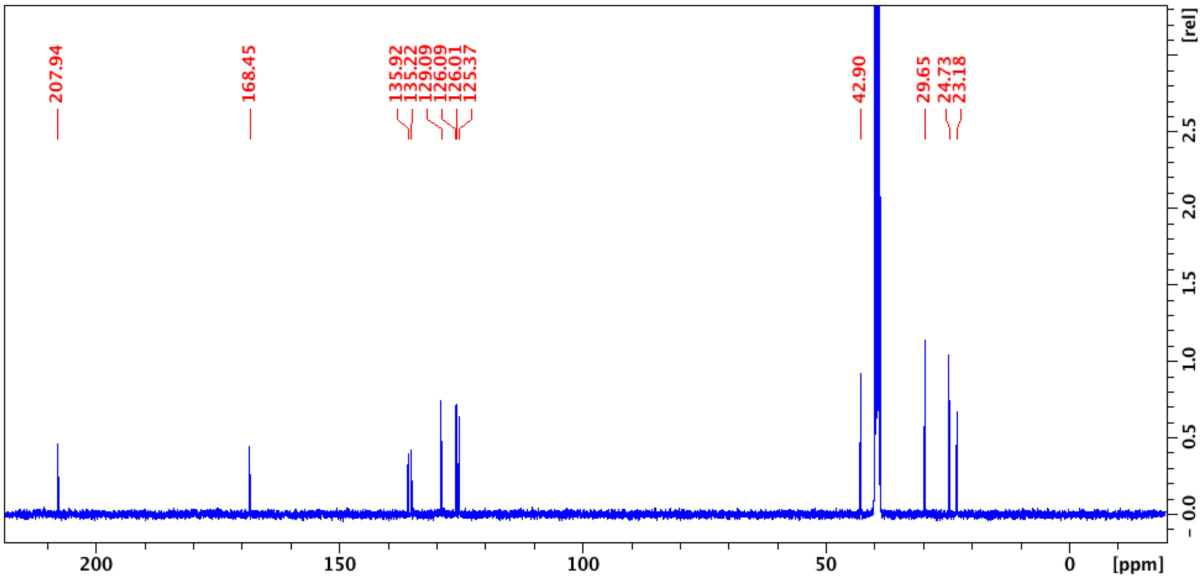

*Figure S2: $^{13}C\ \{^1H\}$ NMR spectrum of compound **2a** in D_6-DMSO at 298 K.*

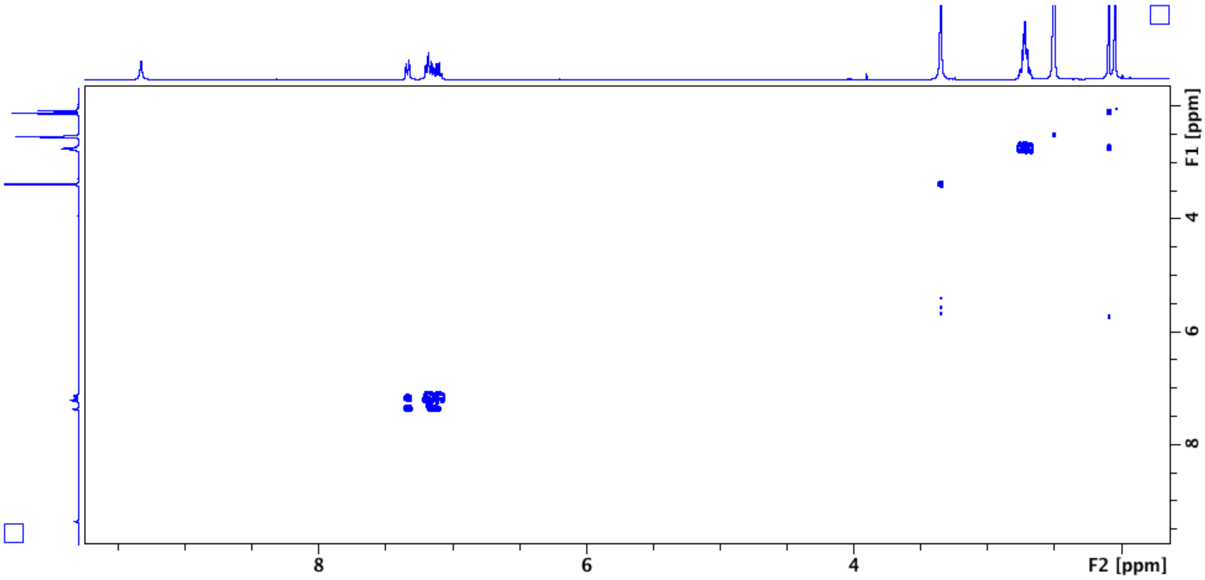

*Figure S3: COSY NMR spectrum of compound **2a** in D_6-DMSO at 298 K.*

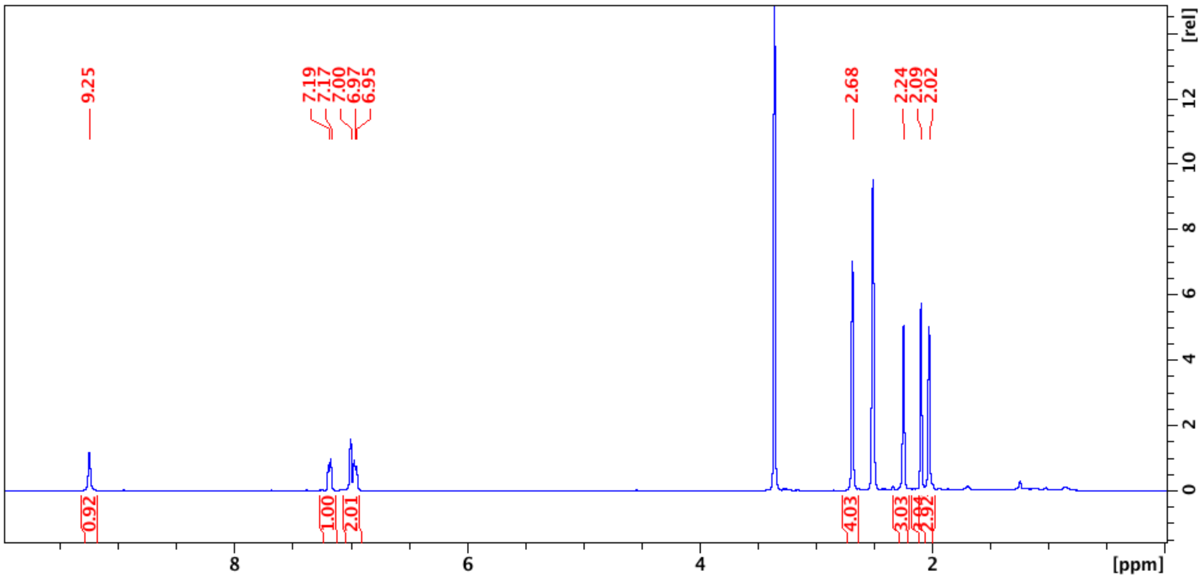

*Figure S4: ^{1}H NMR spectrum of compound **2b** in D_6-DMSO at 298 K.*

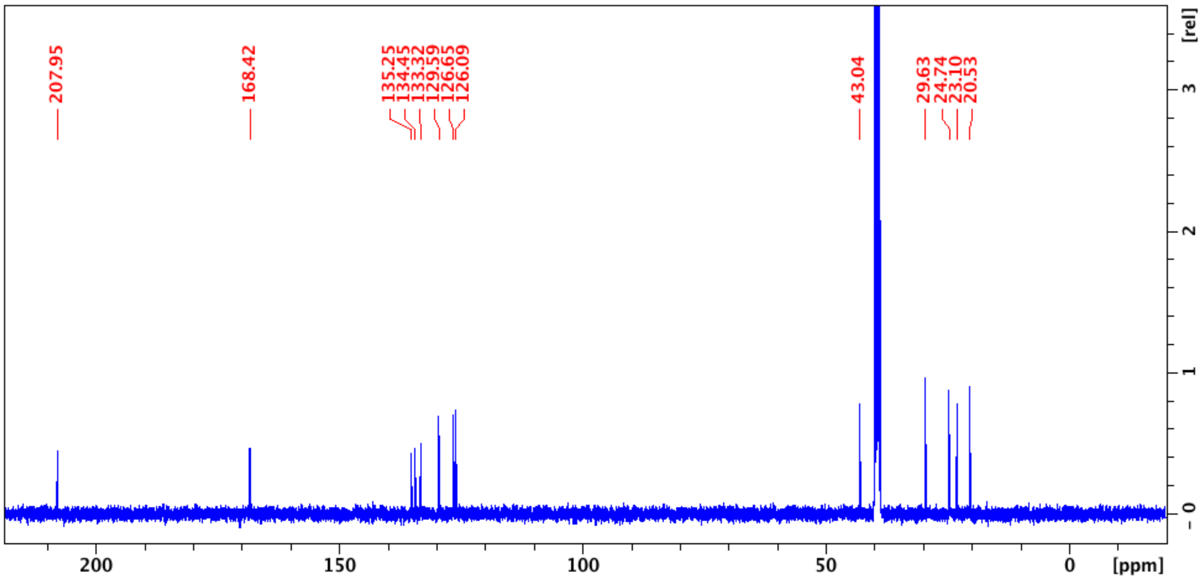

*Figure S5: ^{13}C {^{1}H} NMR spectrum of compound **2b** in D_6-DMSO at 298 K.*

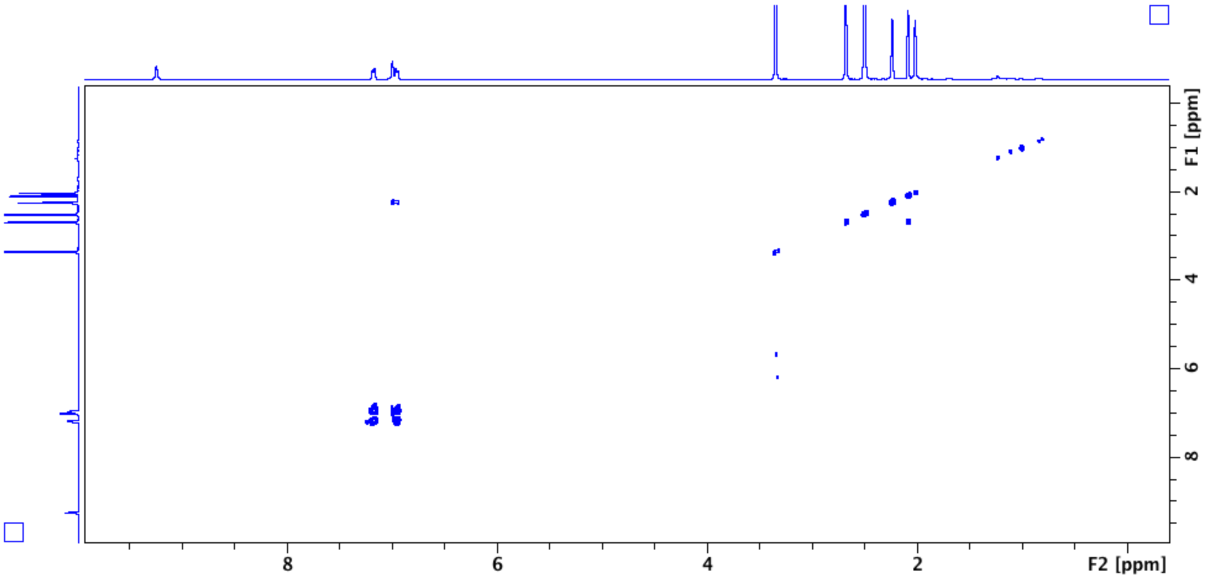

*Figure S6: COSY NMR spectrum of compound **2b** in D_6-DMSO at 298 K.*

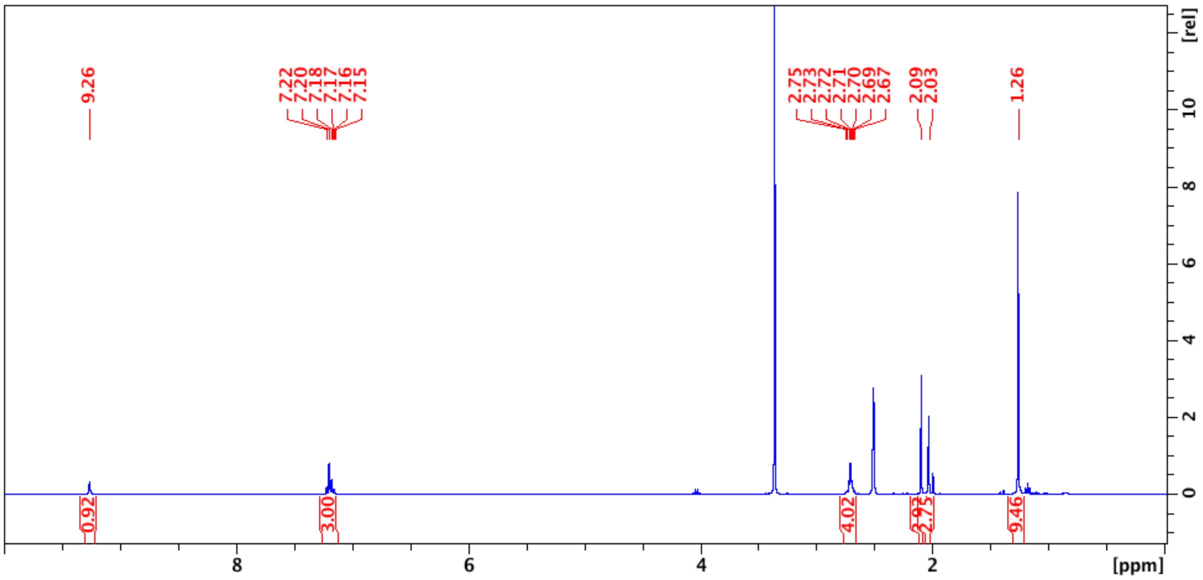

*Figure S7: ^{1}H NMR spectrum of compound **2c** in D_6-DMSO at 298 K. NB. This spectra contains traces of ethyl acetate which could not be removed under reduced pressure for extensive time.*

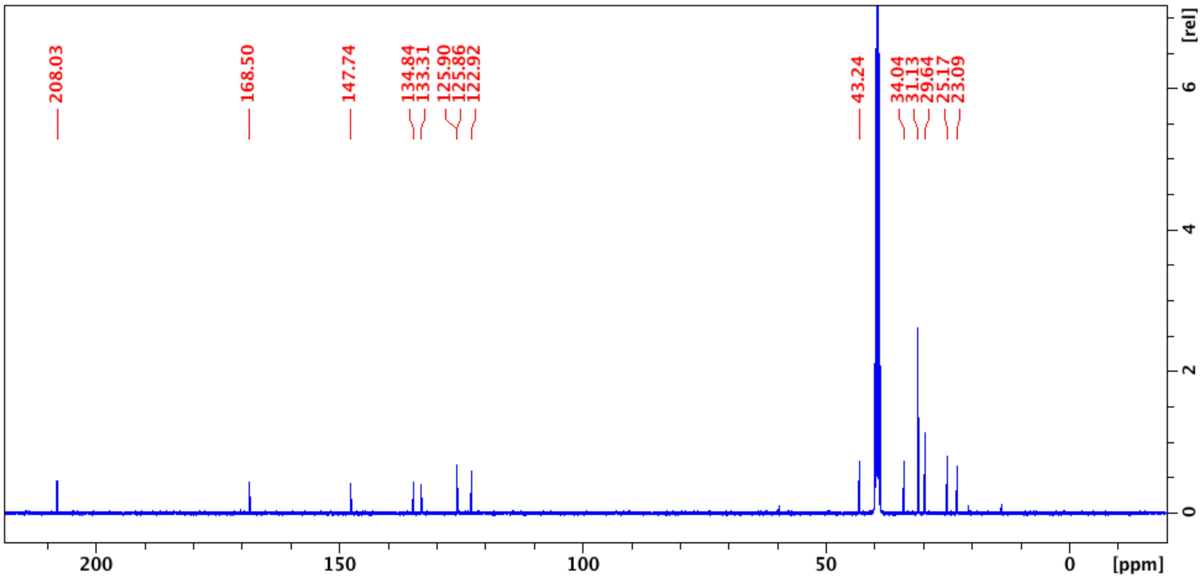

Figure S8: $^{13}C\ \{^{1}H\}$ NMR spectrum of compound ***2c*** *in D_6-DMSO at 298 K.*

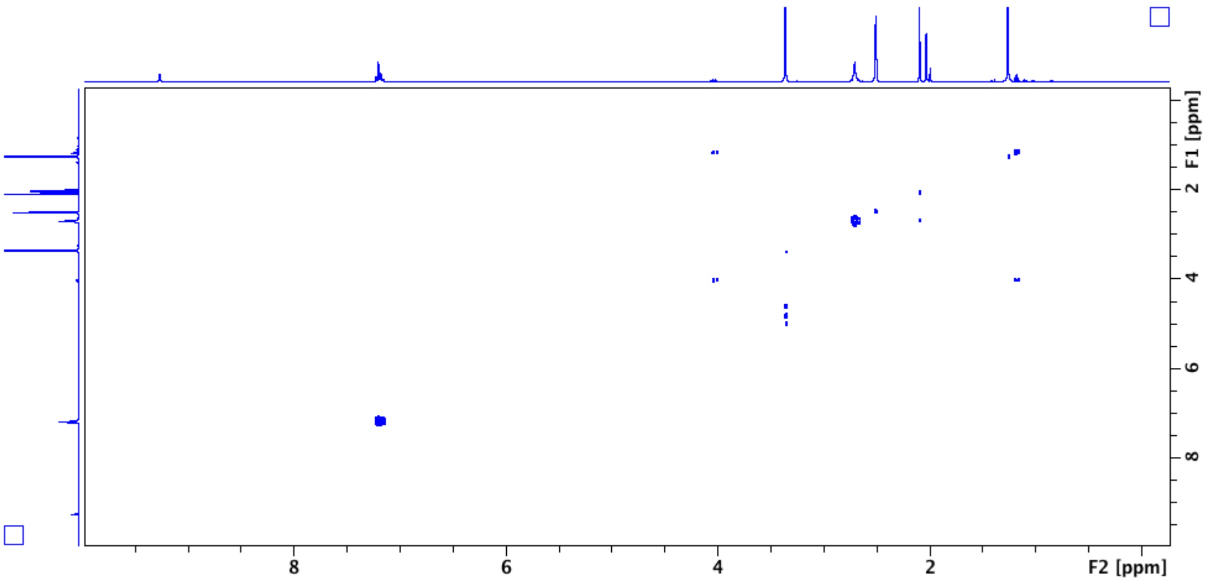

Figure S9: COSY NMR spectrum of compound ***2c*** *in D_6-DMSO at 298 K.*

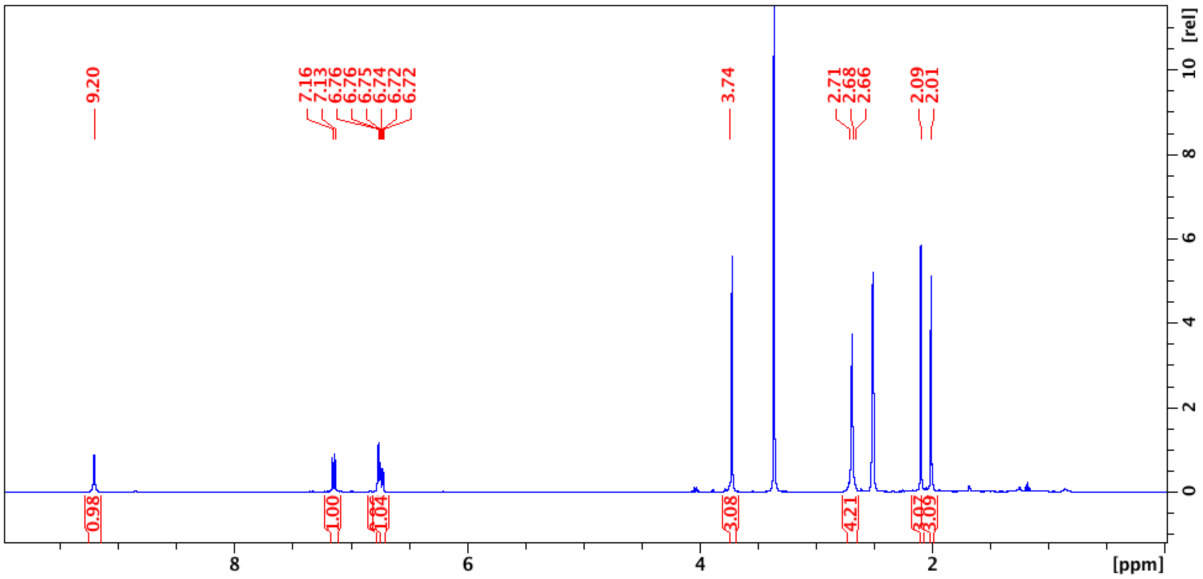

*Figure S10: ^{1}H NMR spectrum of compound **2d** in D_6-DMSO at 298 K.*

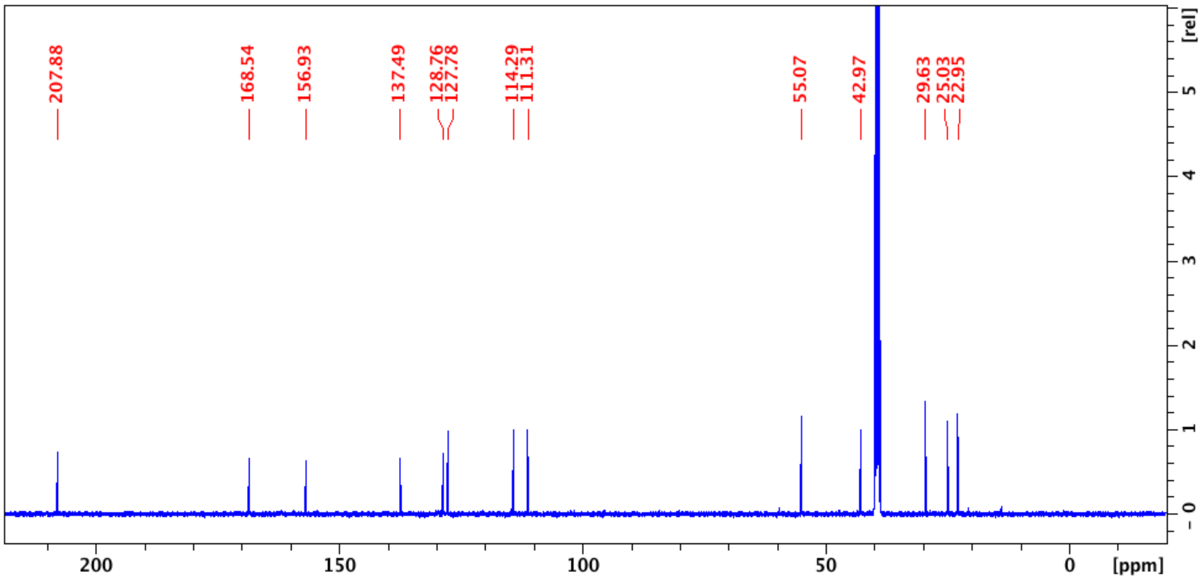

*Figure S11: ^{13}C {^{1}H} NMR spectrum of compound **2d** in D_6-DMSO at 298 K.*

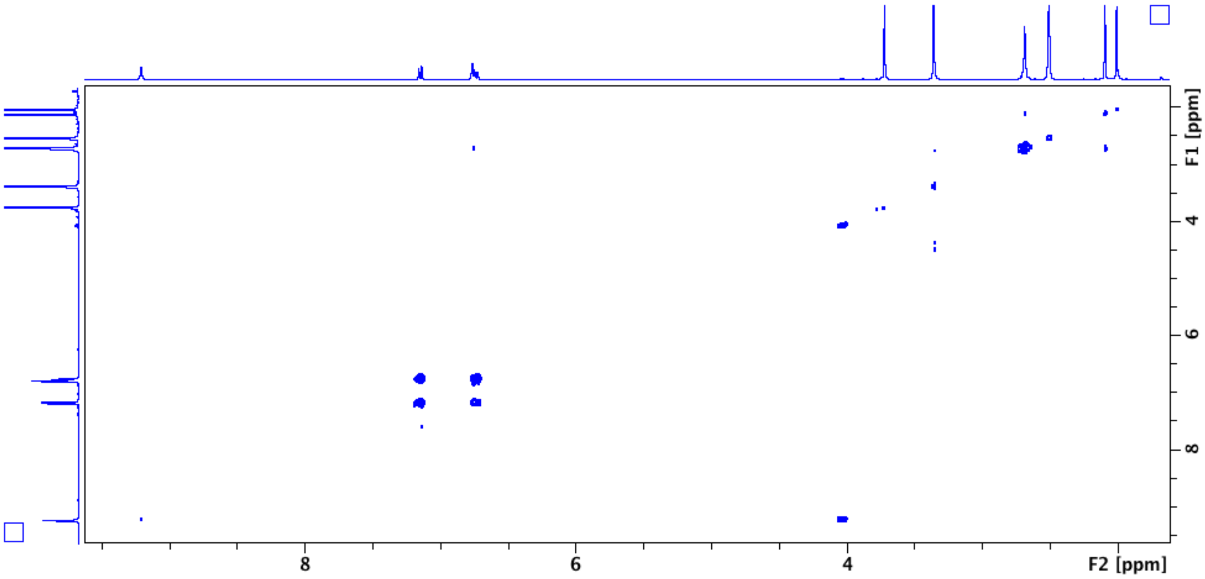

*Figure S12: COSY NMR spectrum of compound **2d** in D_6-DMSO at 298 K.*

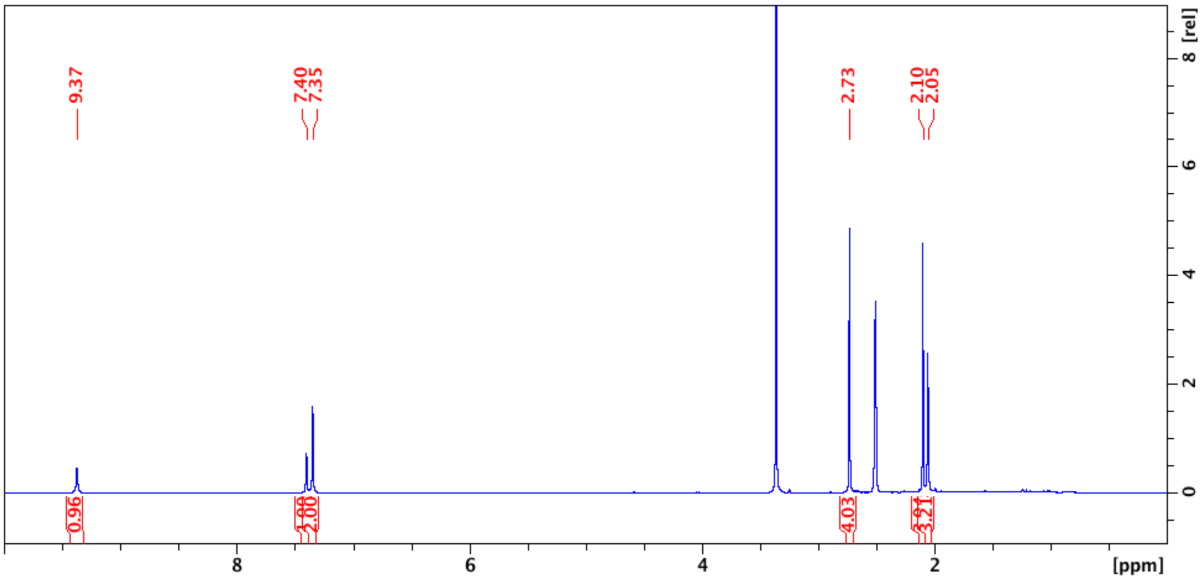

*Figure S13: ^{1}H NMR spectrum of compound **2e** in D_6-DMSO at 298 K.*

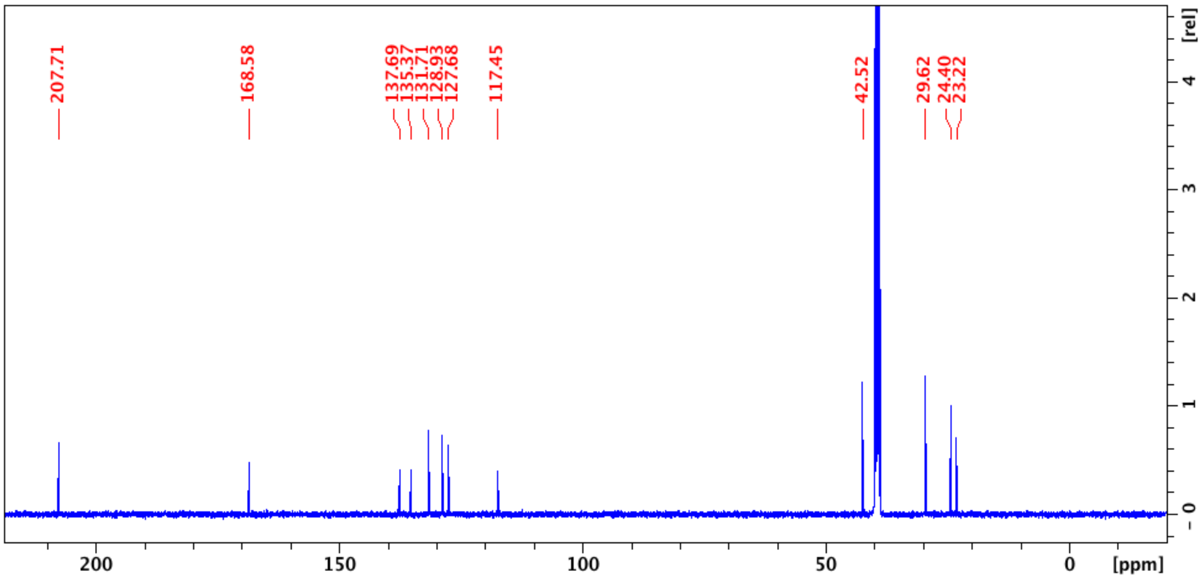

*Figure S14: $^{13}C\{^1H\}$ NMR spectrum of compound **2e** in D_6-DMSO at 298 K.*

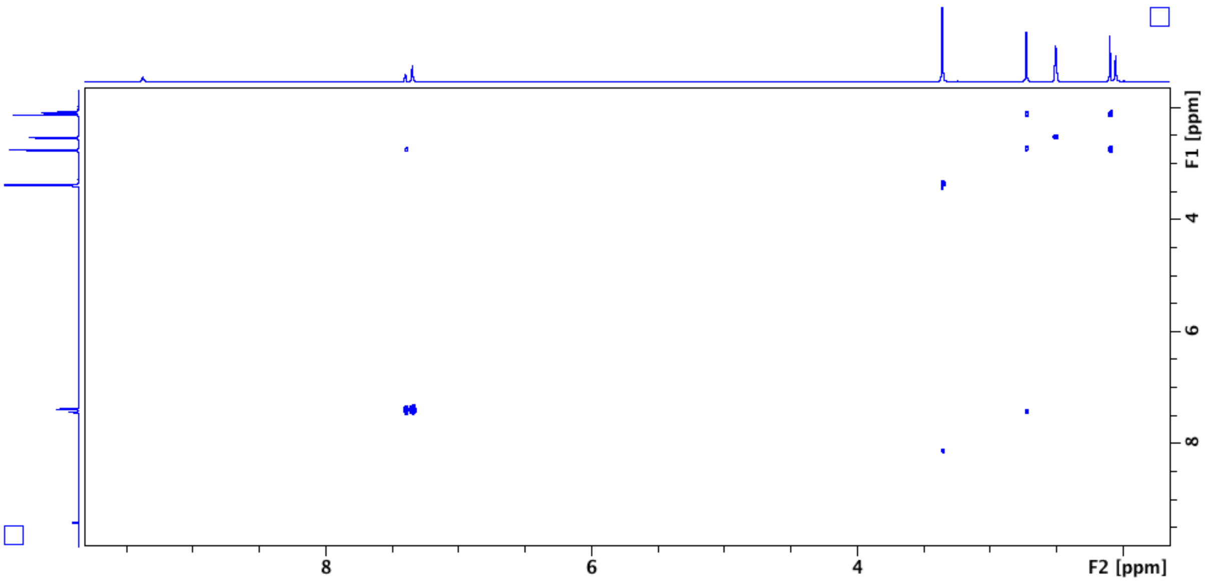

*Figure S15: COSY NMR spectrum of compound **2e** in D_6-DMSO at 298 K.*

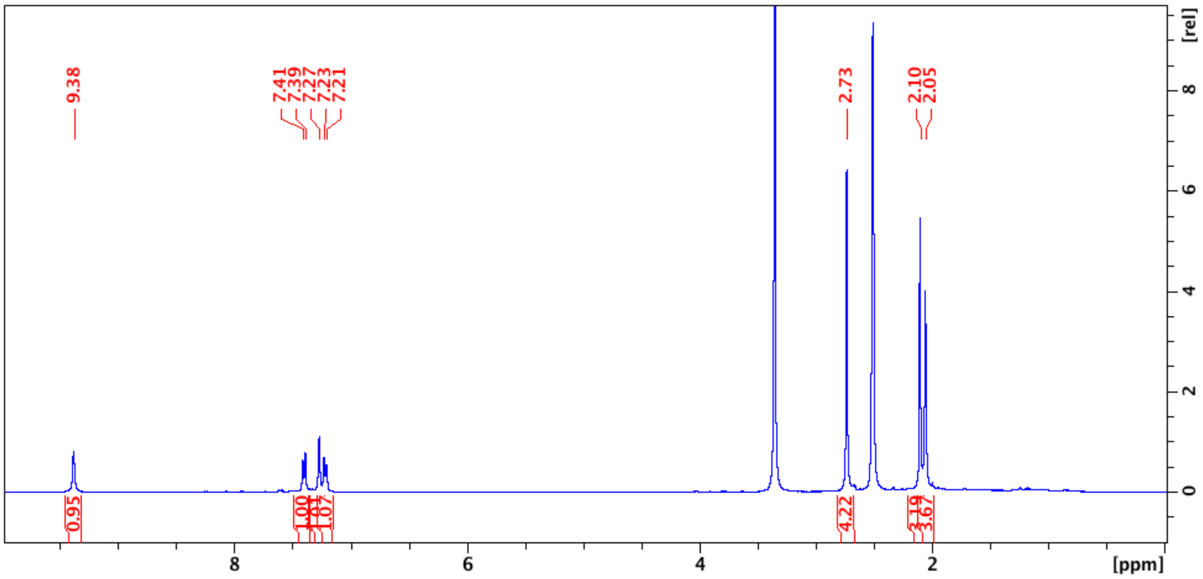

*Figure S16: ^{1}H NMR spectrum of compound **2f** in D_6-DMSO at 298 K.*

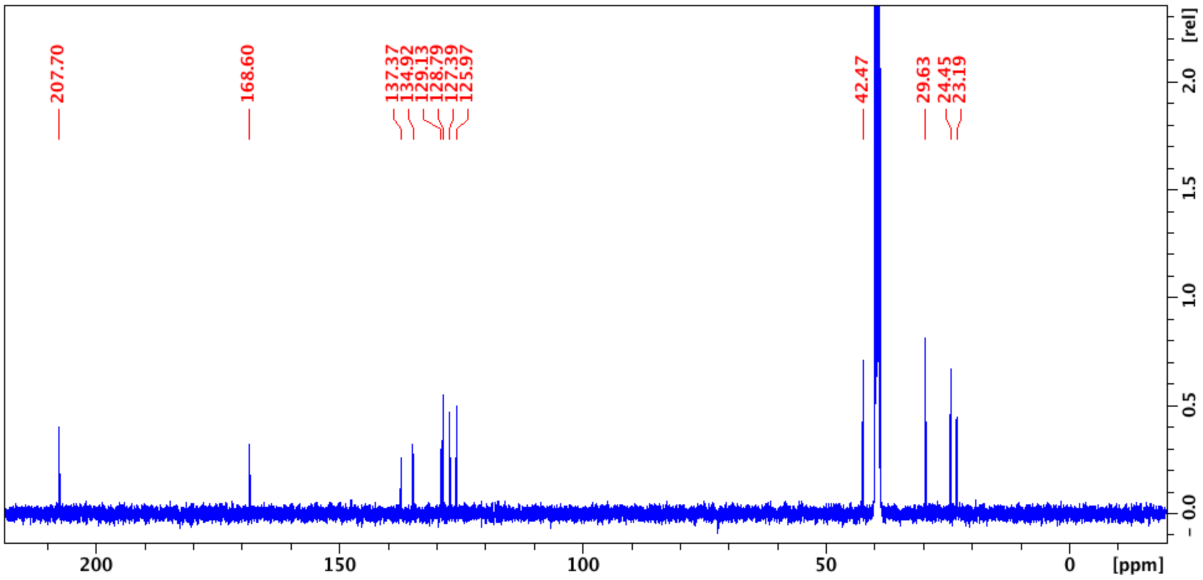

*Figure S17: ^{13}C {^{1}H} NMR spectrum of compound **2f** in D_6-DMSO at 298 K.*

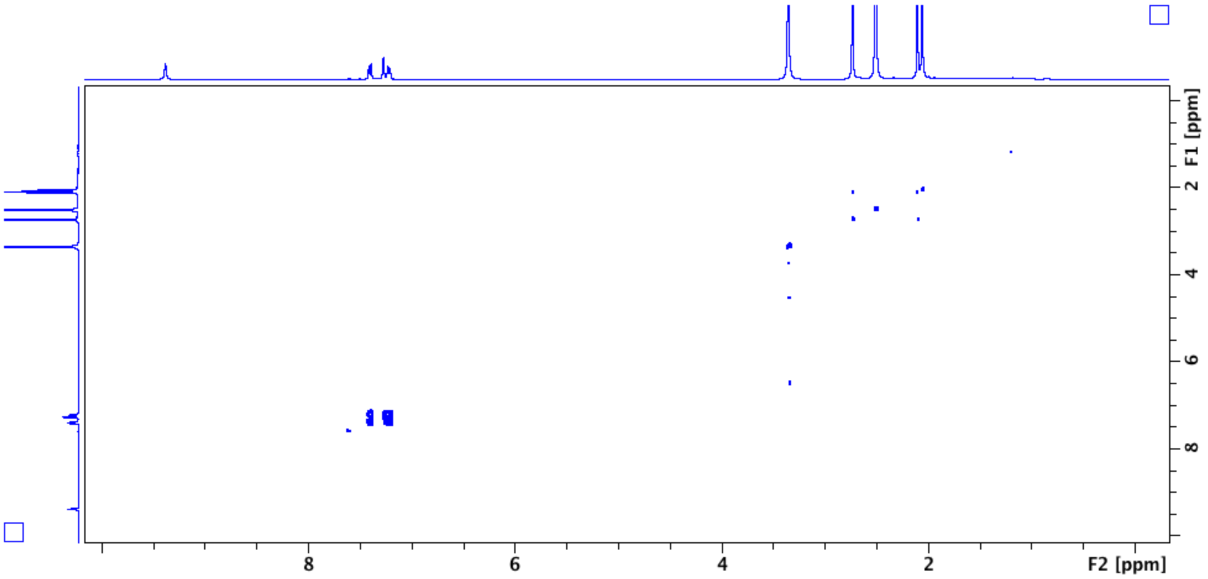

*Figure S18: COSY NMR spectrum of compound **2f** in D_6-DMSO at 298 K.*

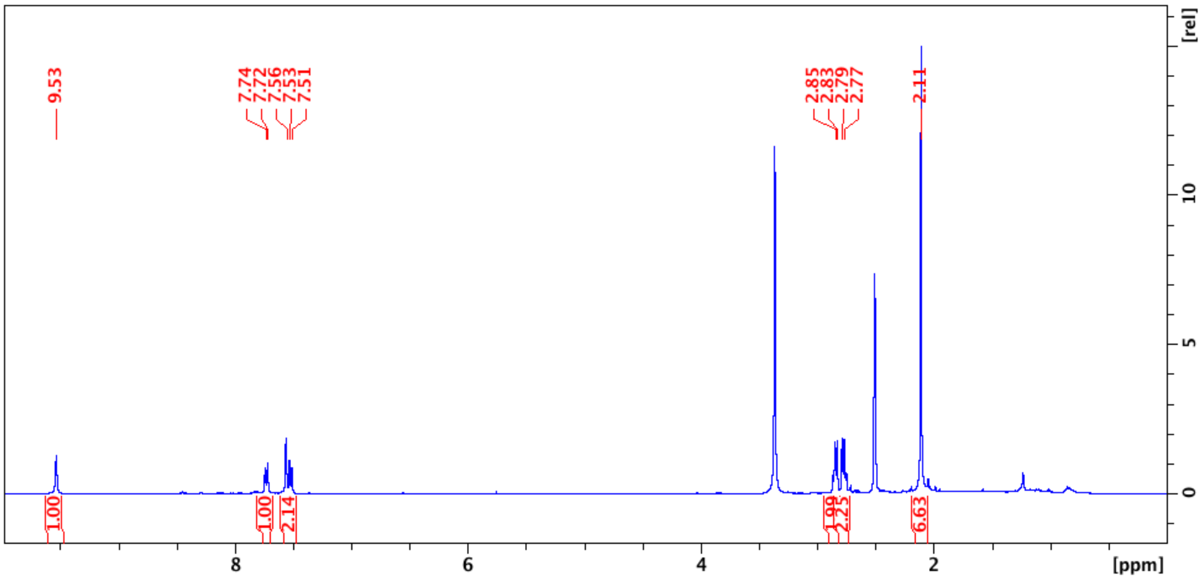

*Figure S19: ^{1}H NMR spectrum of compound **2g** in D_6-DMSO at 298 K.*

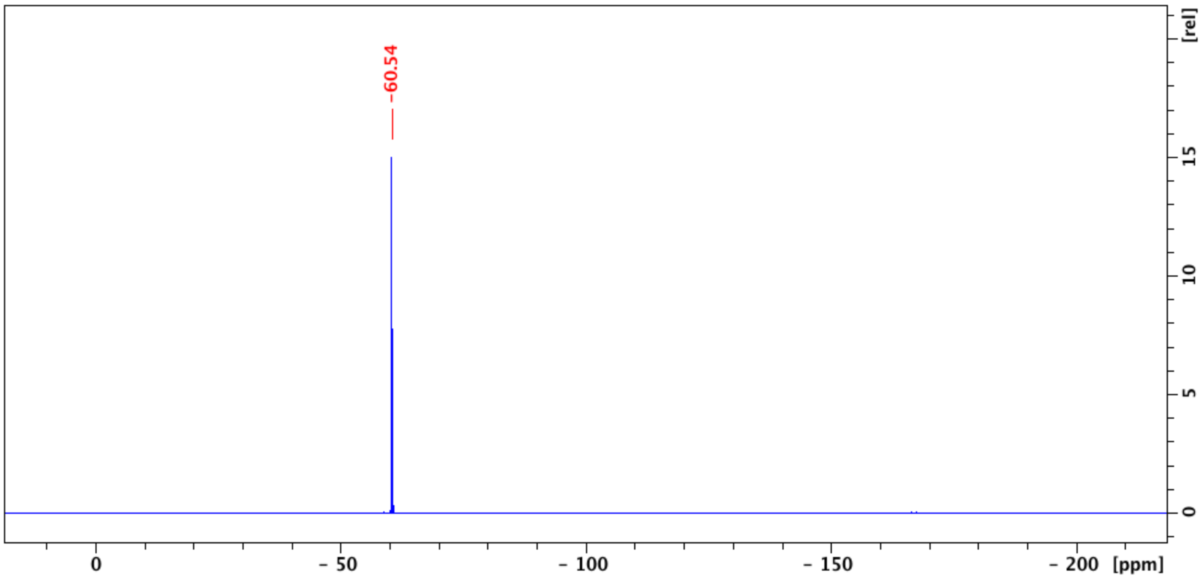

*Figure S20: ^{19}F {^{1}H} NMR spectrum of compound **2g** in D_6-DMSO at 298 K.*

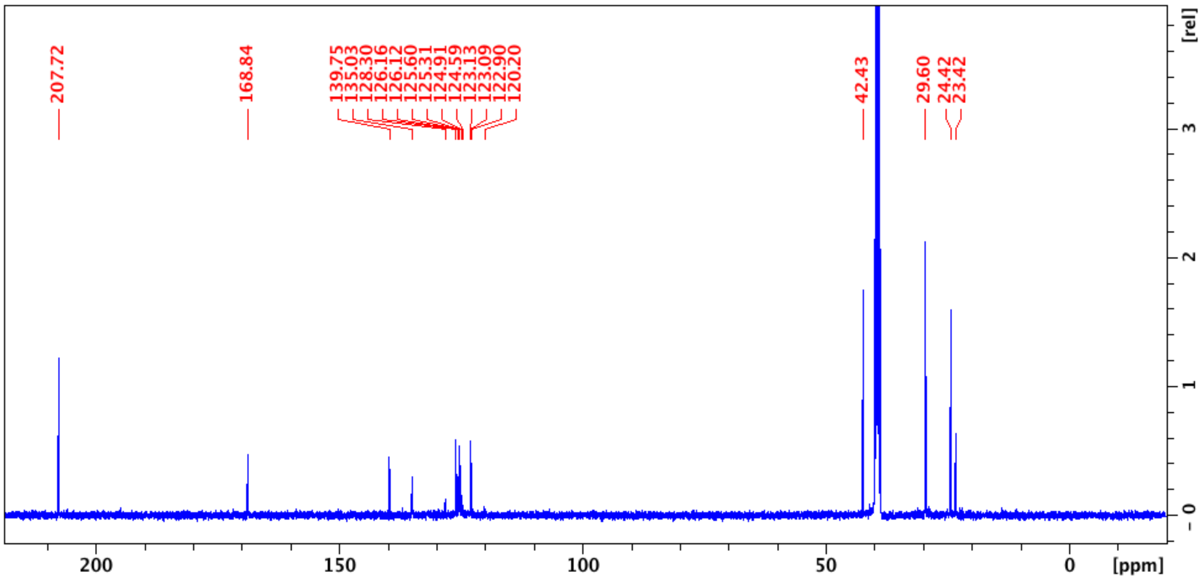

*Figure S21: ^{13}C {^{1}H} NMR spectrum of compound **2g** in D_6-DMSO at 298 K.*

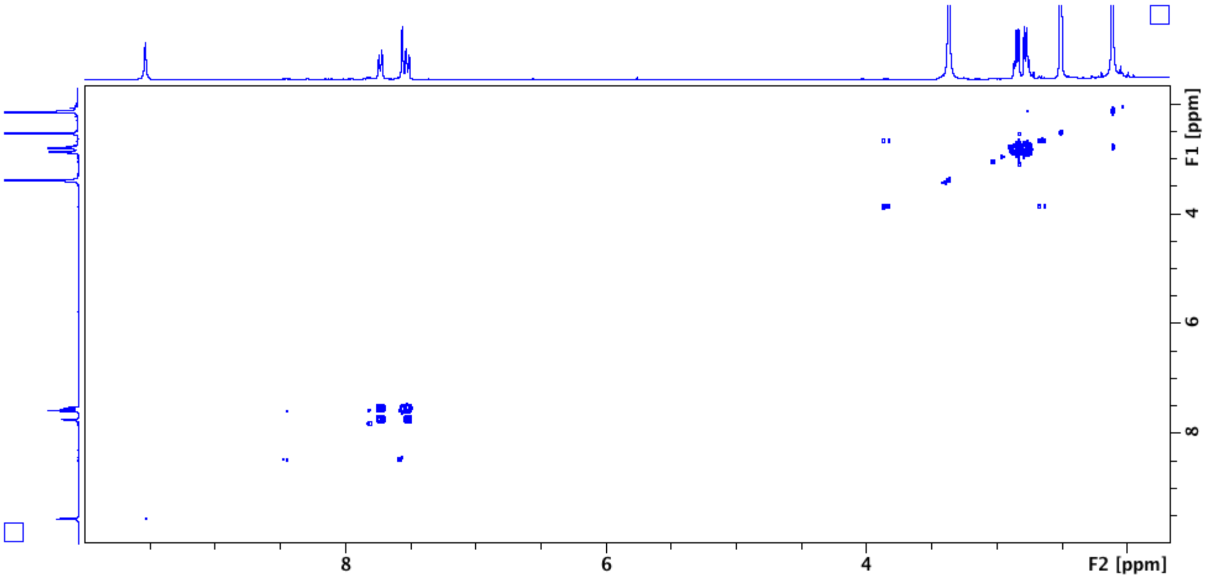

*Figure S22: COSY NMR spectrum of compound **2g** in D_6-DMSO at 298 K.*

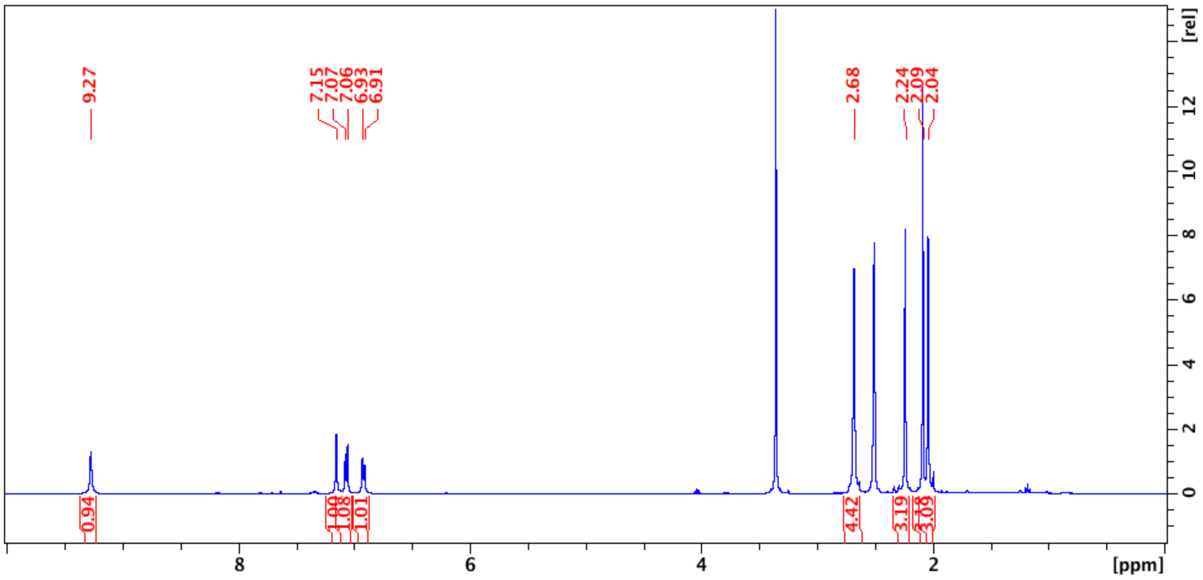

*Figure S23: ^{1}H NMR spectrum of compound **2h** in D_6-DMSO at 298 K.*

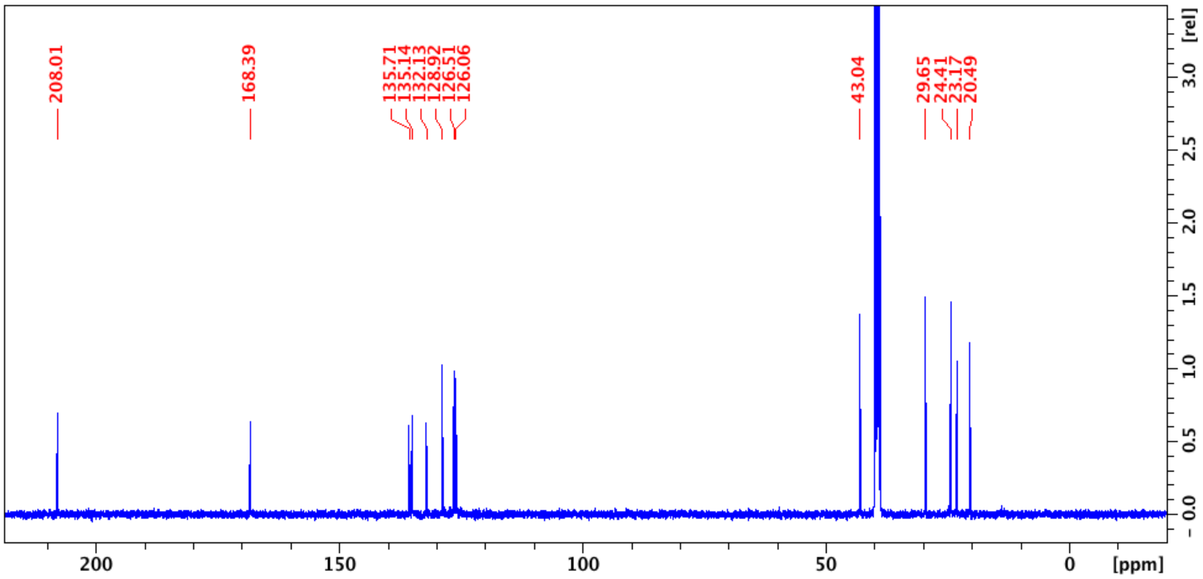

*Figure S24: $^{13}C\{^{1}H\}$ NMR spectrum of compound **2h** in D_6-DMSO at 298 K.*

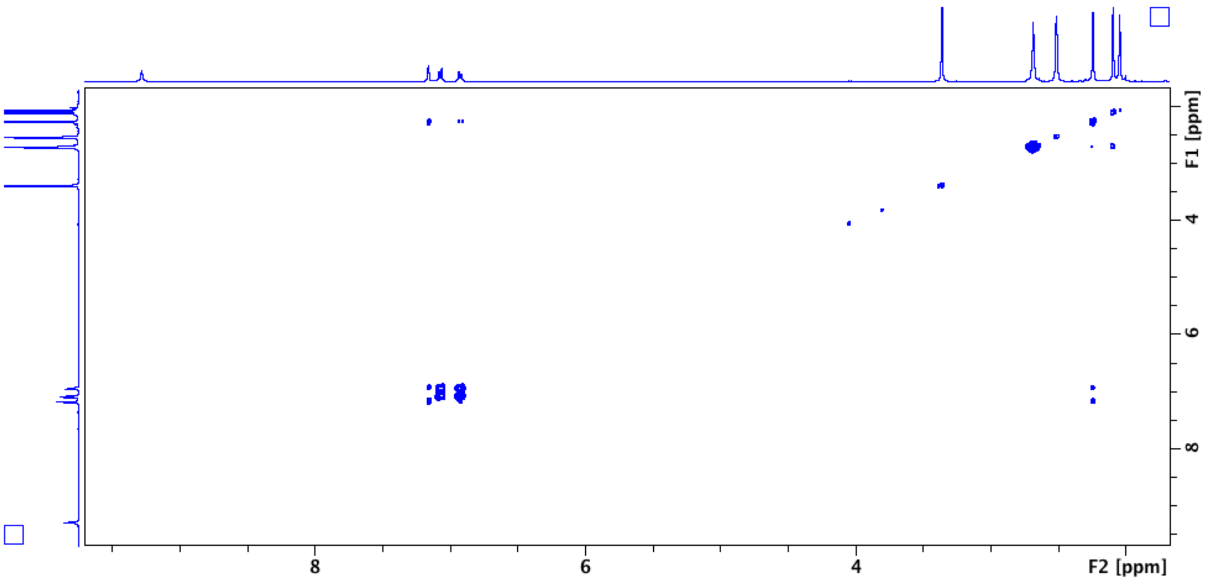

*Figure S25: COSY NMR spectrum of compound **2h** in D_6-DMSO at 298 K.*

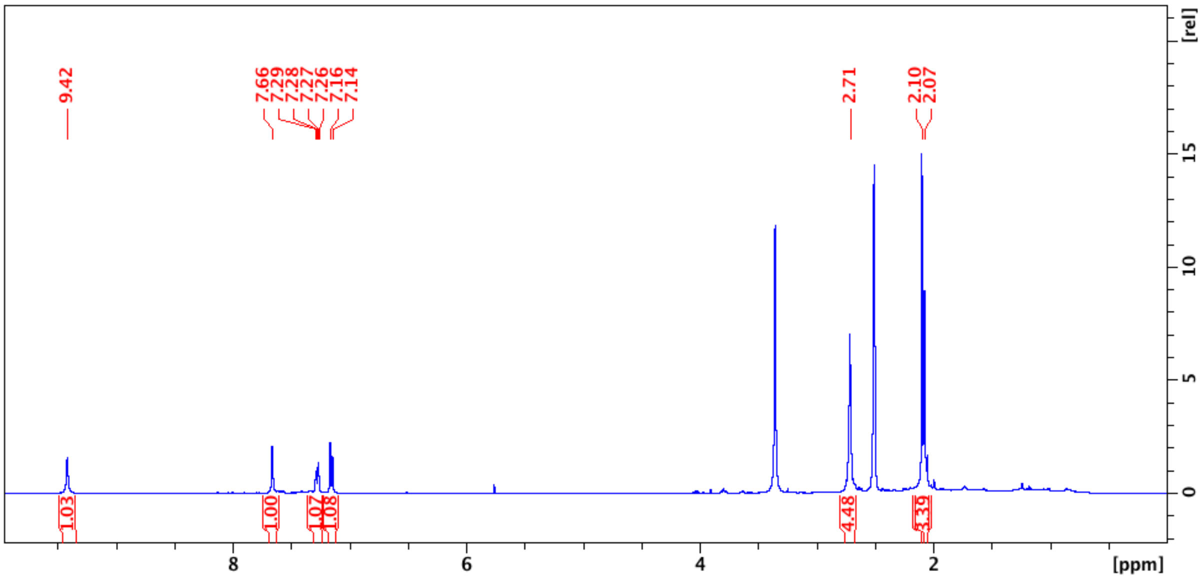

*Figure S26: ^{1}H NMR spectrum of compound **2i** in D_6-DMSO at 298 K.*

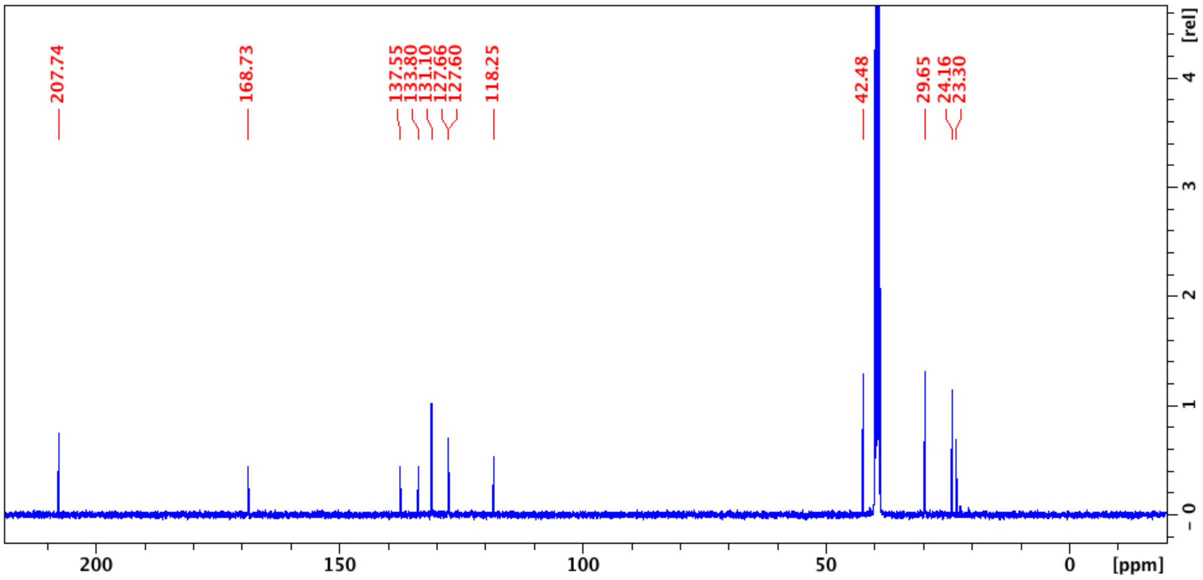

*Figure S27: $^{13}C\{^{1}H\}$ NMR spectrum of compound **2i** in D_6-DMSO at 298 K.*

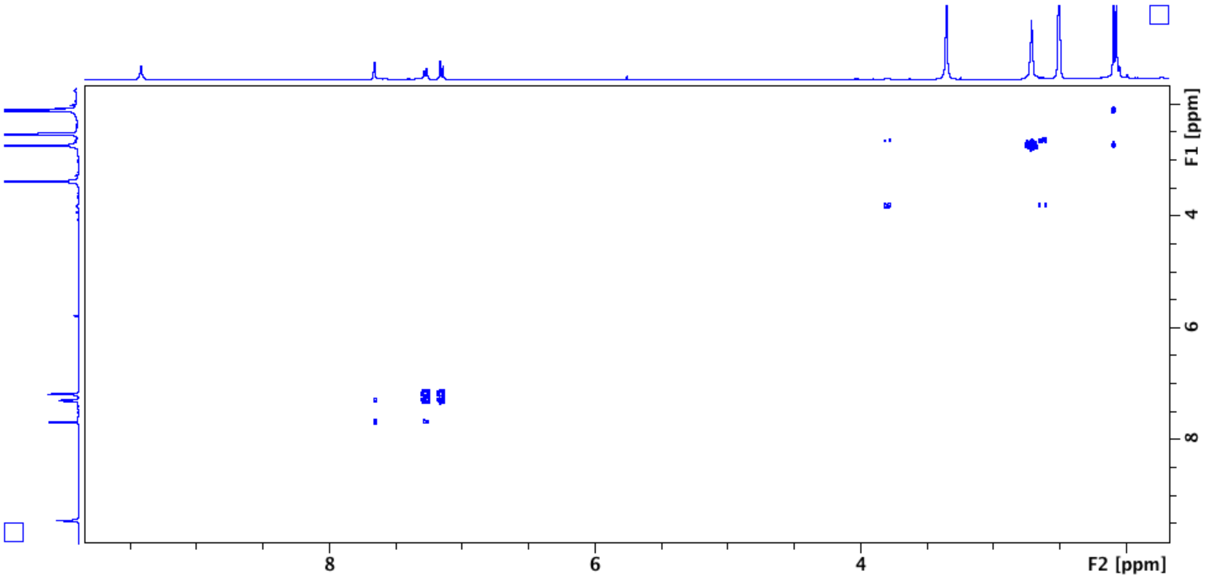

*Figure S28: COSY NMR spectrum of compound **2i** in D_6-DMSO at 298 K.*

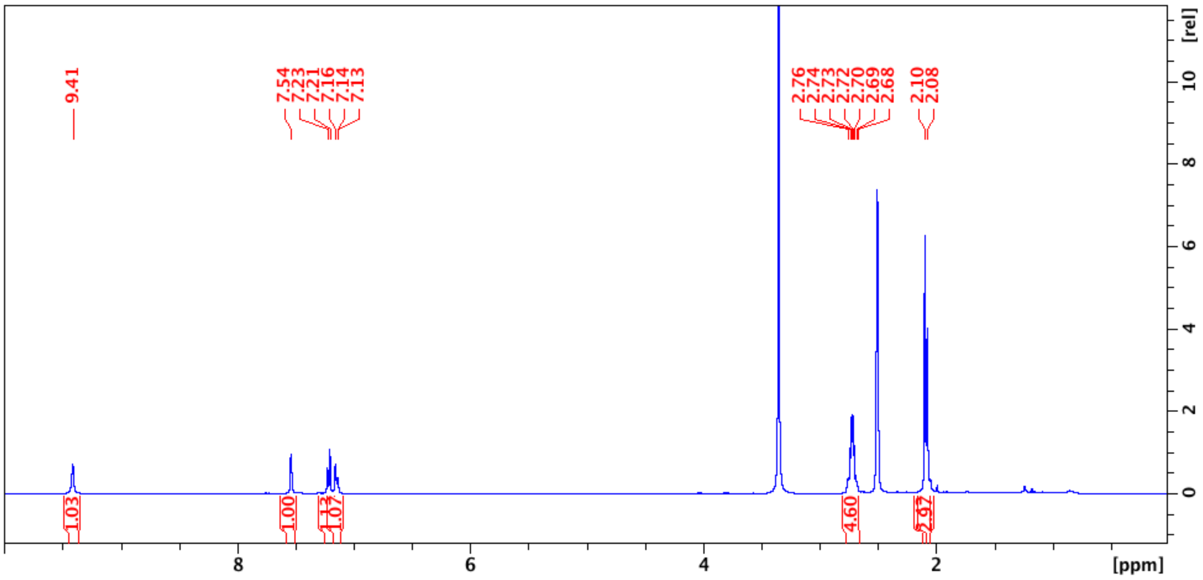

*Figure S29: ^{1}H NMR spectrum of compound **2j** in D_6-DMSO at 298 K.*

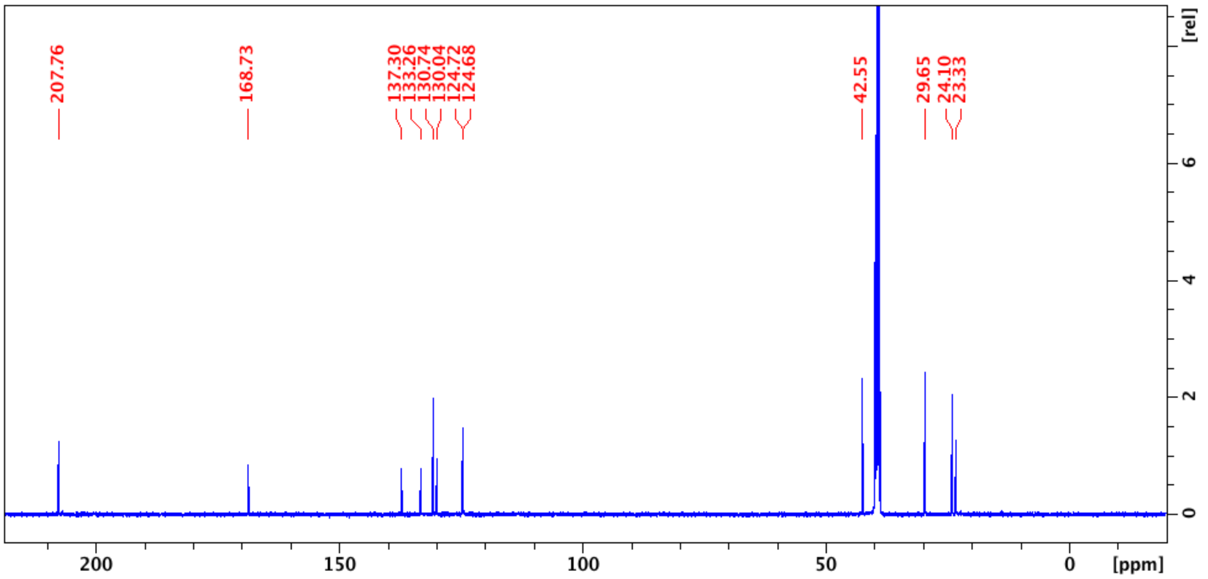

*Figure S30: $^{13}C\ \{^{1}H\}$ NMR spectrum of compound **2j** in D_6-DMSO at 298 K.*

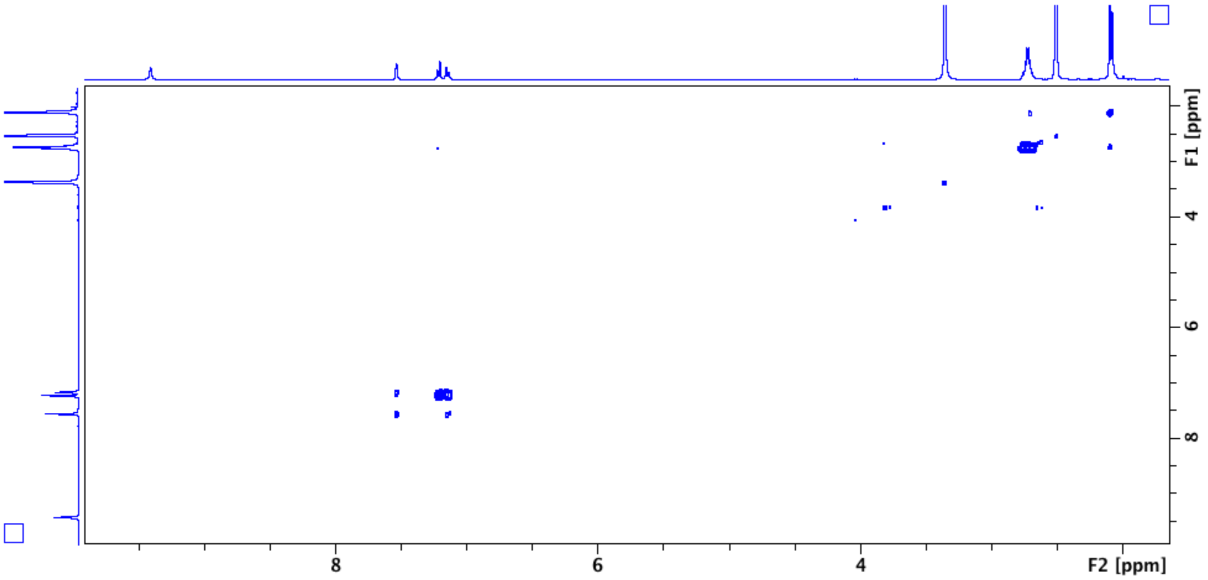

*Figure S31: COSY NMR spectrum of compound **2j** in D_6-DMSO at 298 K.*

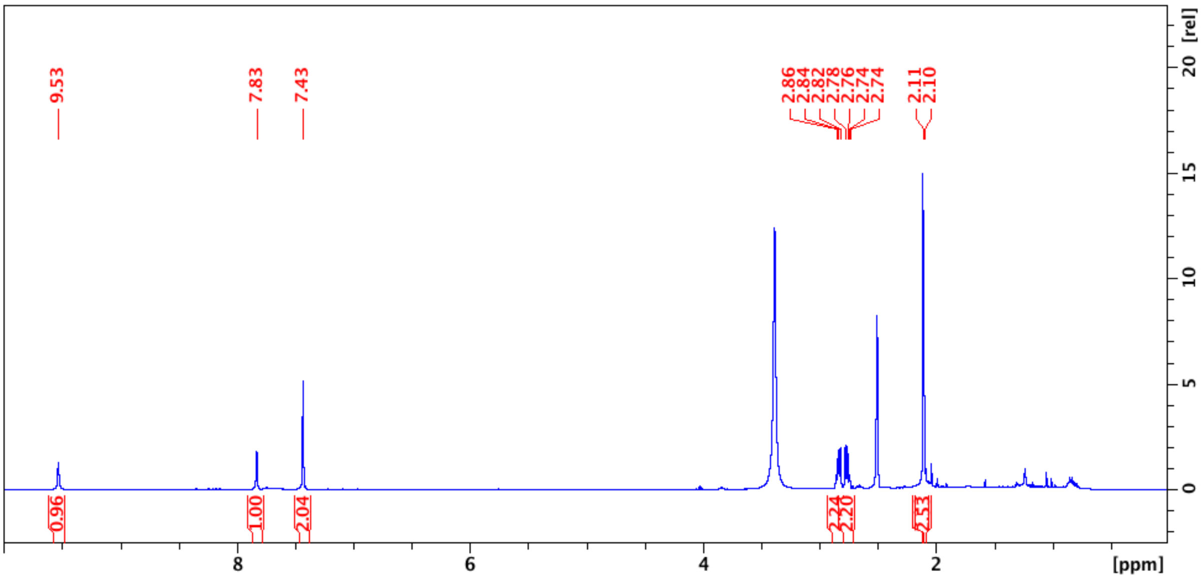

Figure S32: ^{1}H NMR spectrum of compound ***2k*** *in D_6-DMSO at 298 K.*

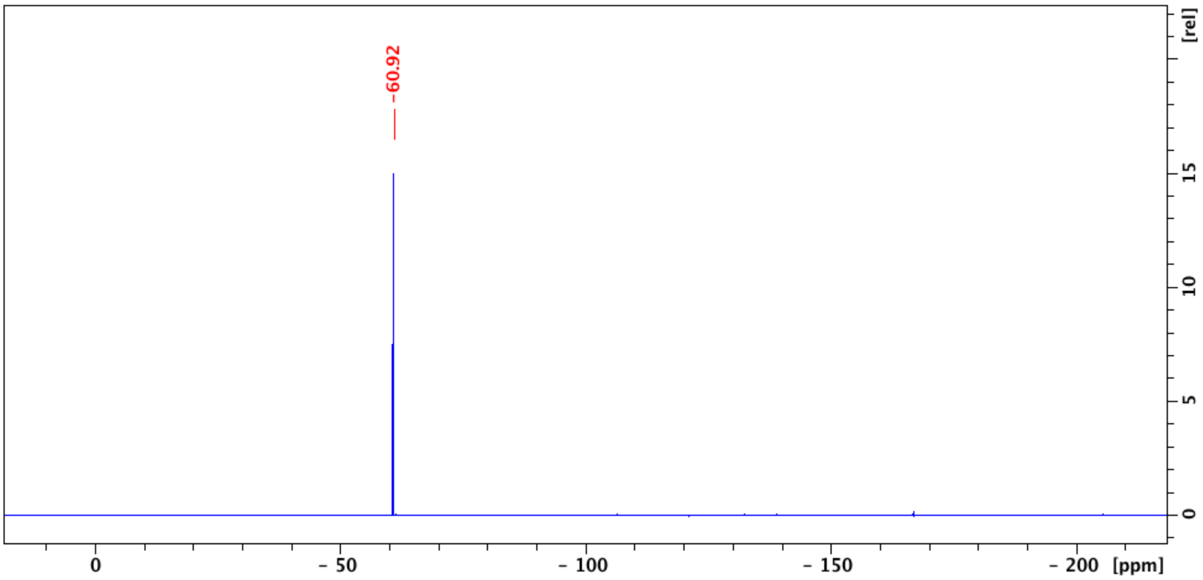

Figure S33: ^{19}F {^{1}H} NMR spectrum of compound ***2k*** *in D_6-DMSO at 298 K.*

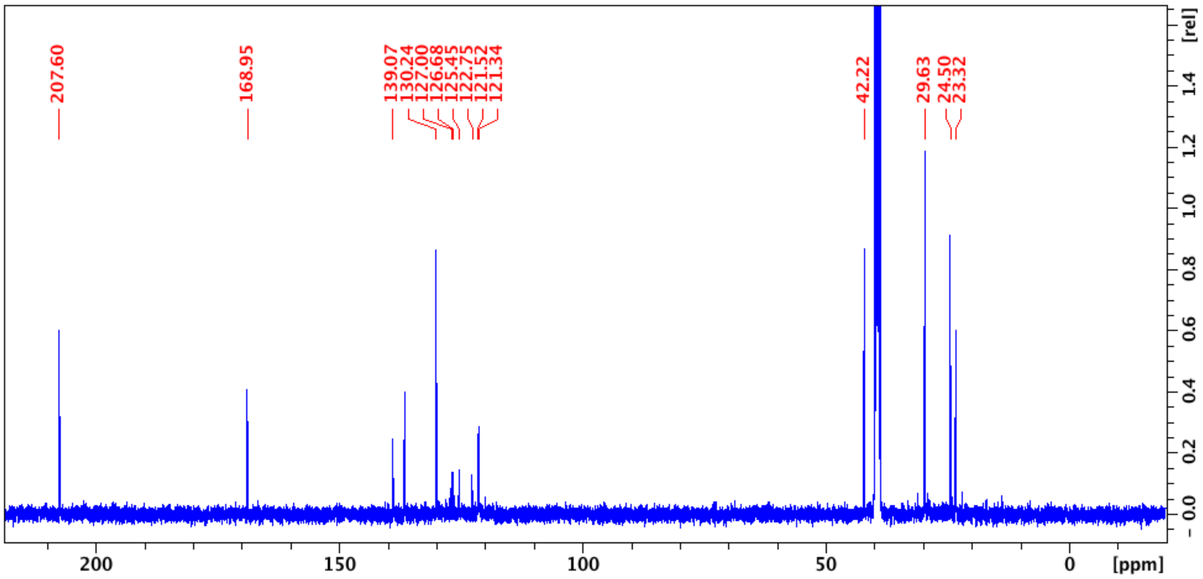

*Figure S34: $^{13}C\ \{^{1}H\}$ NMR spectrum of compound **2k** in D_6-DMSO at 298 K.*

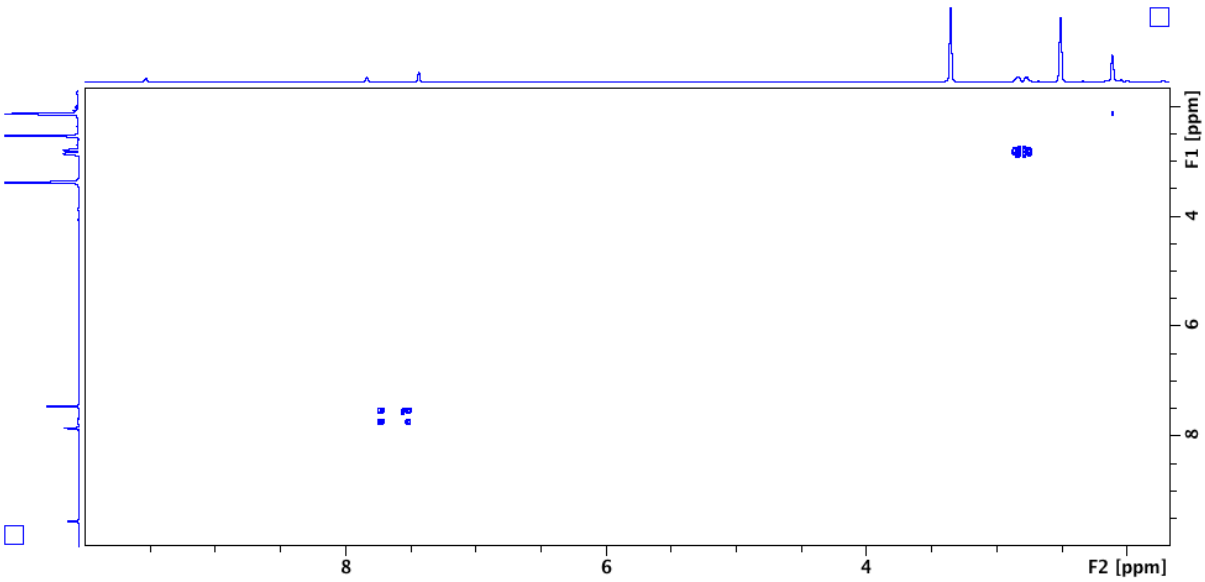

*Figure S35: COSY NMR spectrum of compound **2k** in D_6-DMSO at 298 K.*

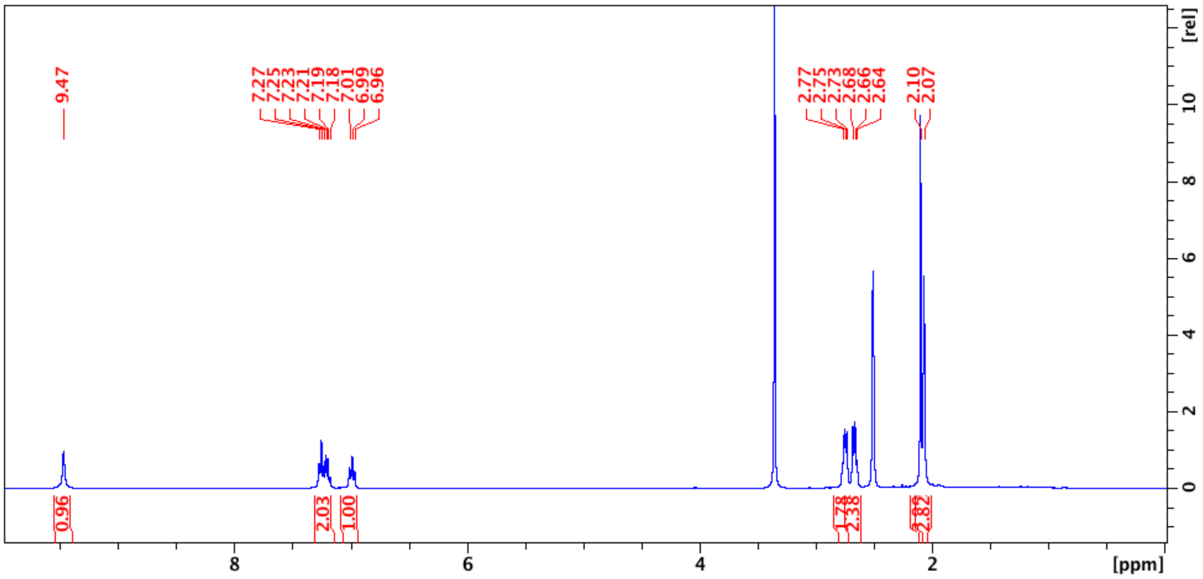

*Figure S36: ^{1}H NMR spectrum of compound **2l** in D_6-DMSO at 298 K.*

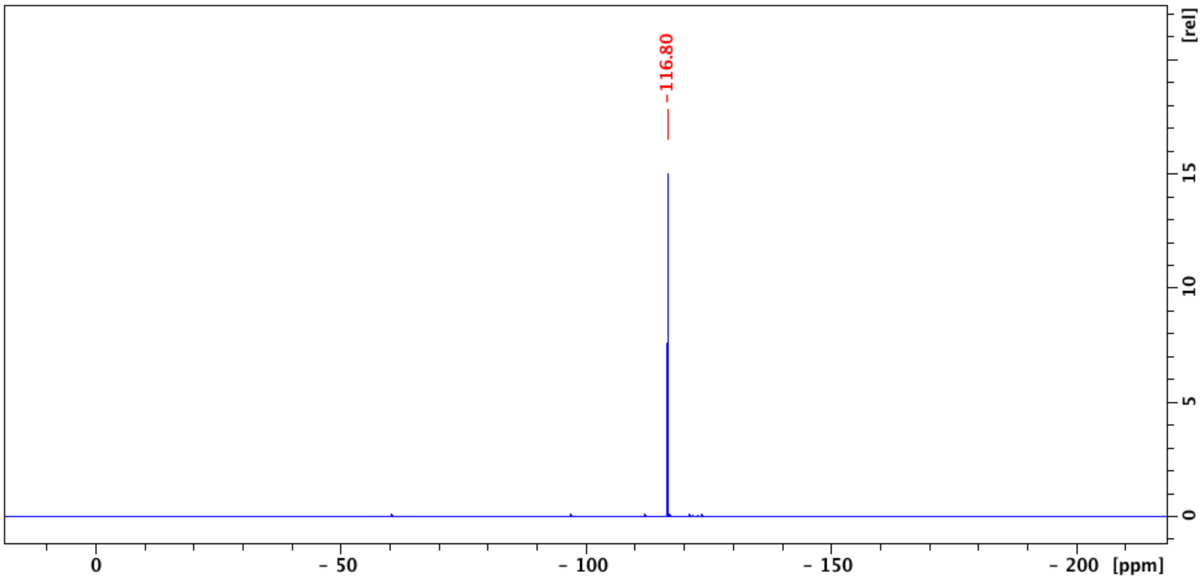

*Figure S37: ^{19}F {^{1}H} NMR spectrum of compound **2l** in D_6-DMSO at 298 K.*

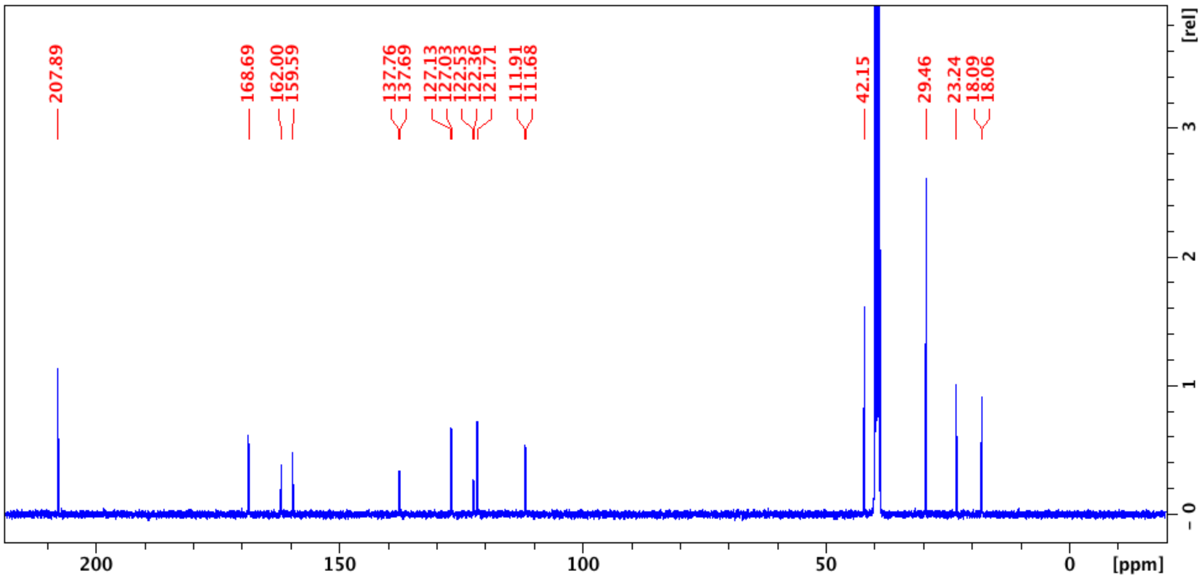

*Figure S38: $^{13}C\{^{1}H\}$ NMR spectrum of compound **2l** in D_6-DMSO at 298 K.*

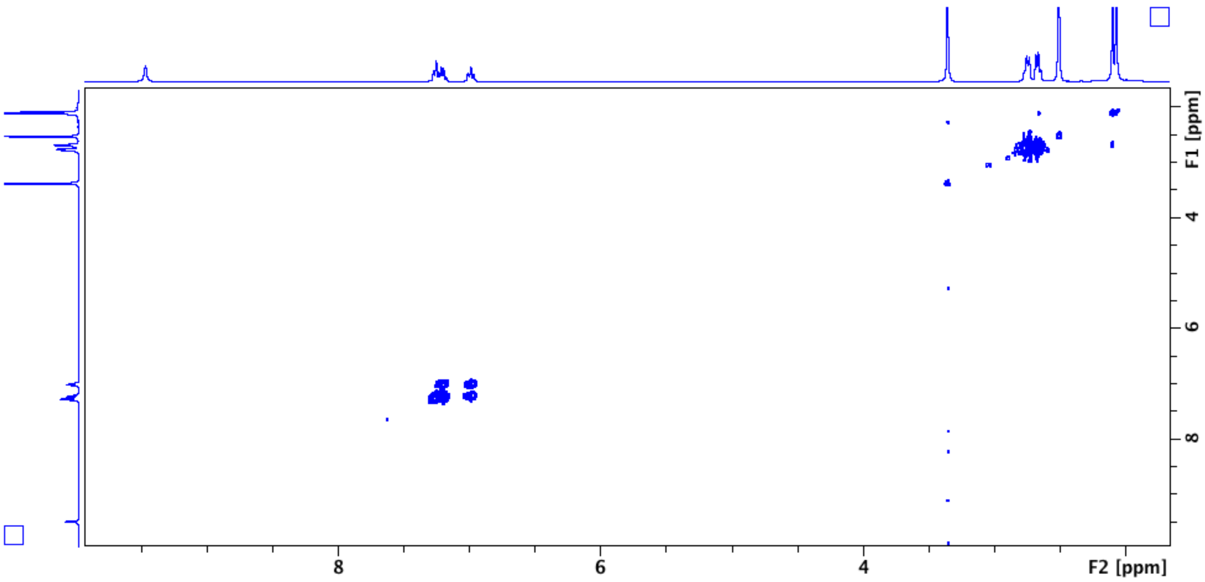

*Figure S39: COSY NMR spectrum of compound **2l** in D_6-DMSO at 298 K.*

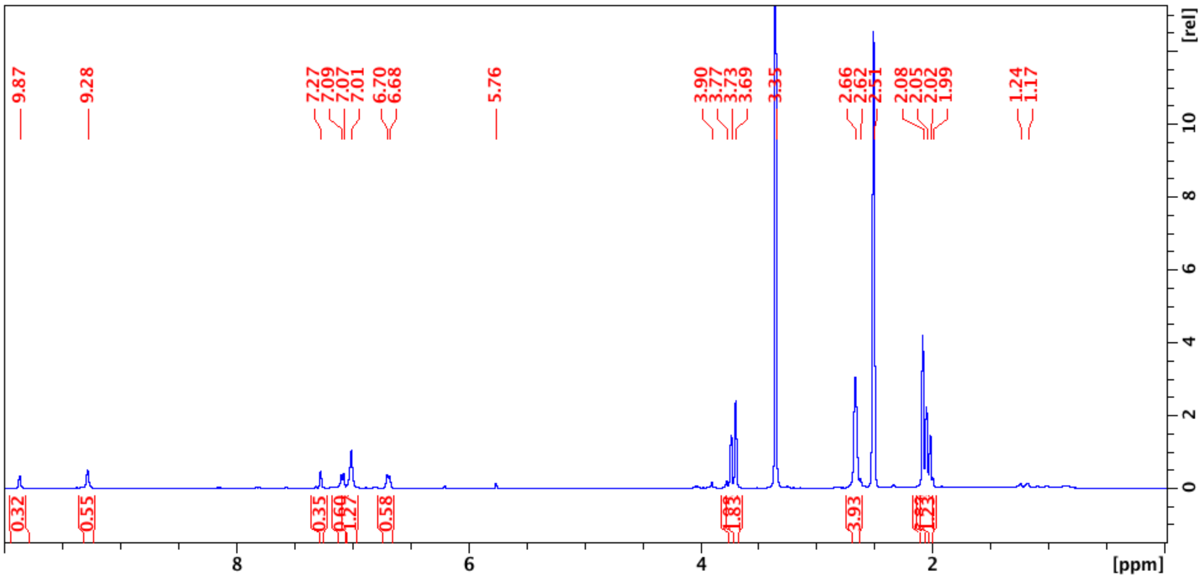

Figure S40: ^{1}H NMR spectrum of mixture of compounds **2ma** and **2mb** in D_6-DMSO at 298 K.

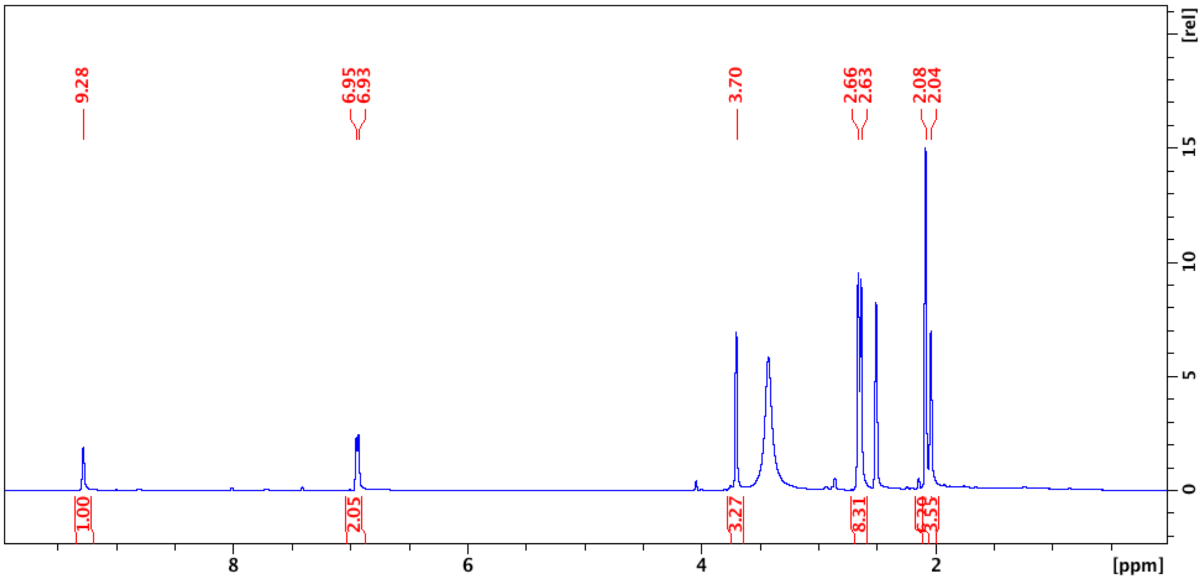

Figure S41: ^{1}H NMR spectrum of compound **2mc** in D_6-DMSO at 298 K.

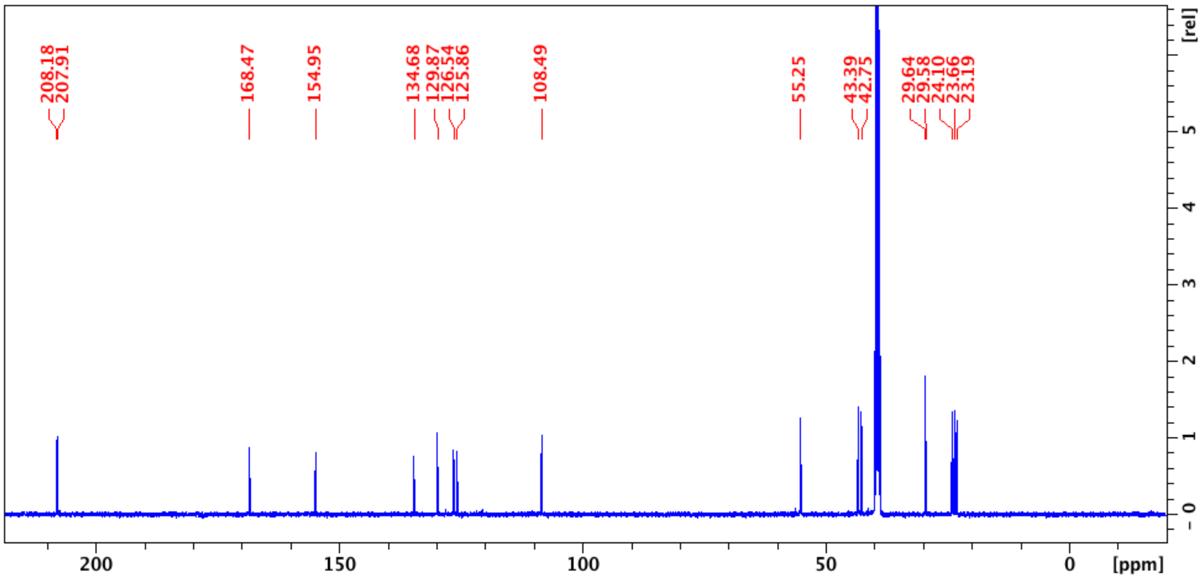

*Figure S42: $^{13}C\{^{1}H\}$ NMR spectrum of compound **2mc** in D_6-DMSO at 298 K.*

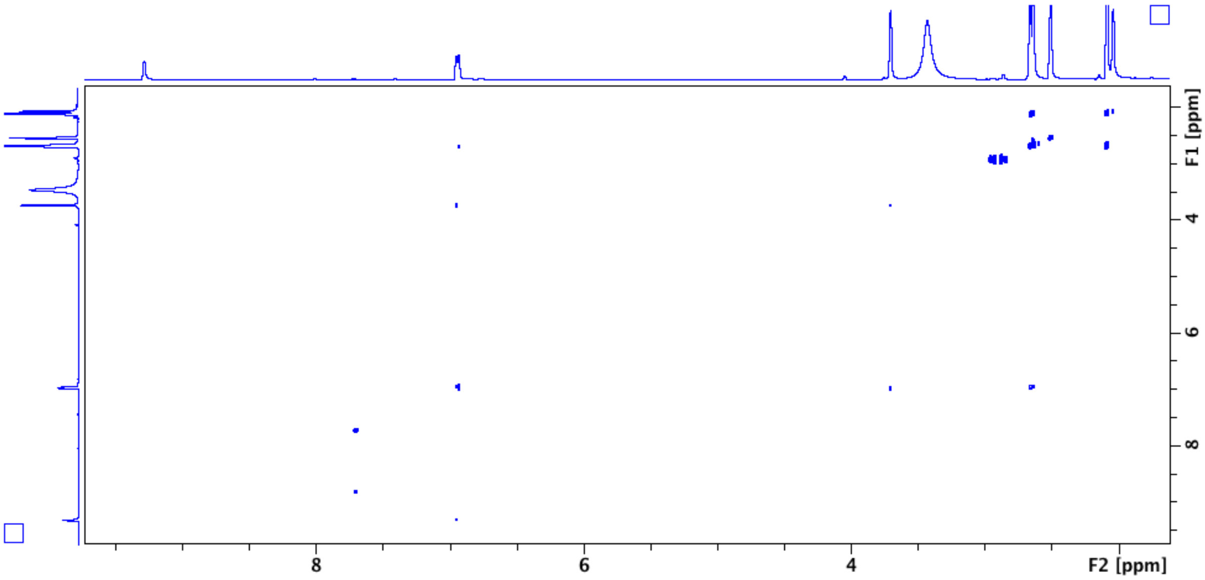

*Figure S43: COSY NMR spectrum of compound **2mc** in D_6-DMSO at 298 K.*

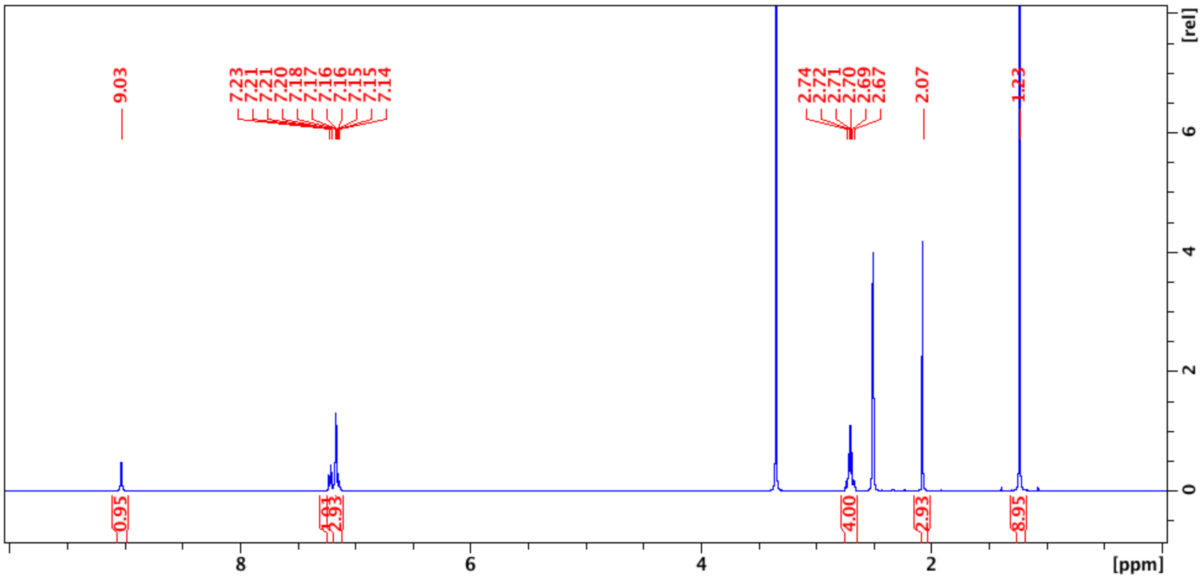

*Figure S44: ^{1}H NMR spectrum of compound **2p** in D_6-DMSO at 298 K.*

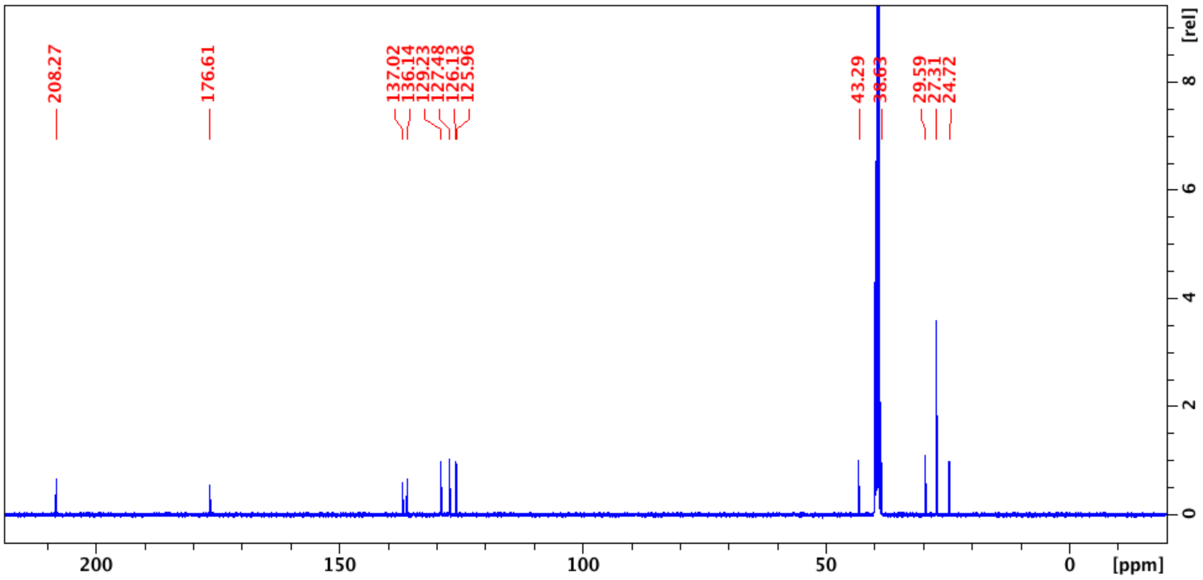

*Figure S45: $^{13}C\ \{^{1}H\}$ NMR spectrum of compound **2p** in D_6-DMSO at 298 K.*

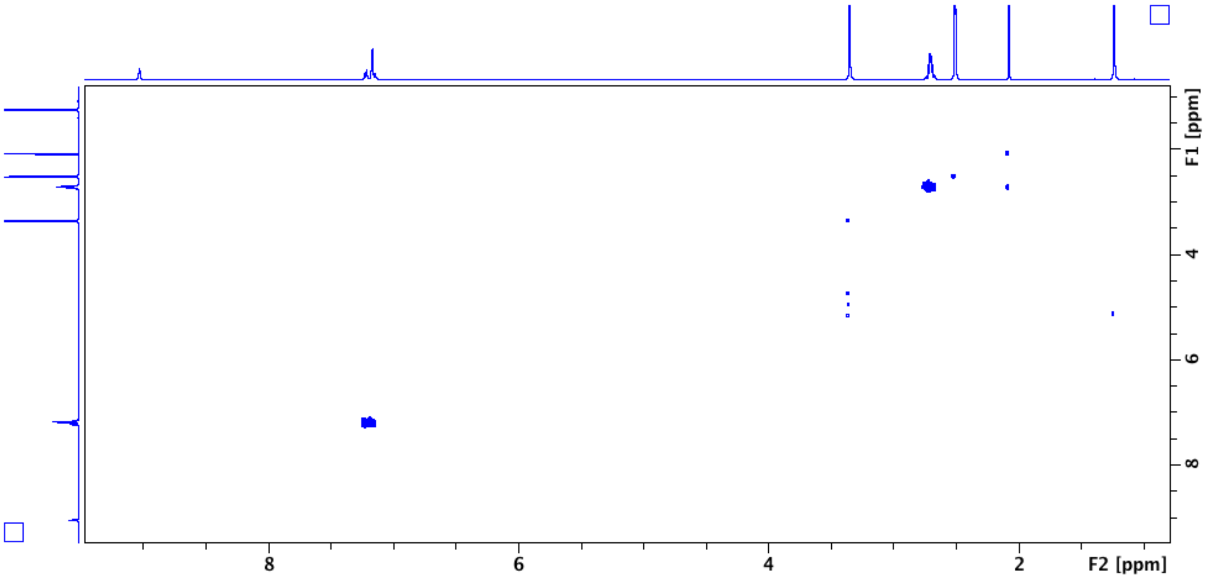

*Figure S46: COSY NMR spectrum of compound **2p** in D_6-DMSO at 298 K.*

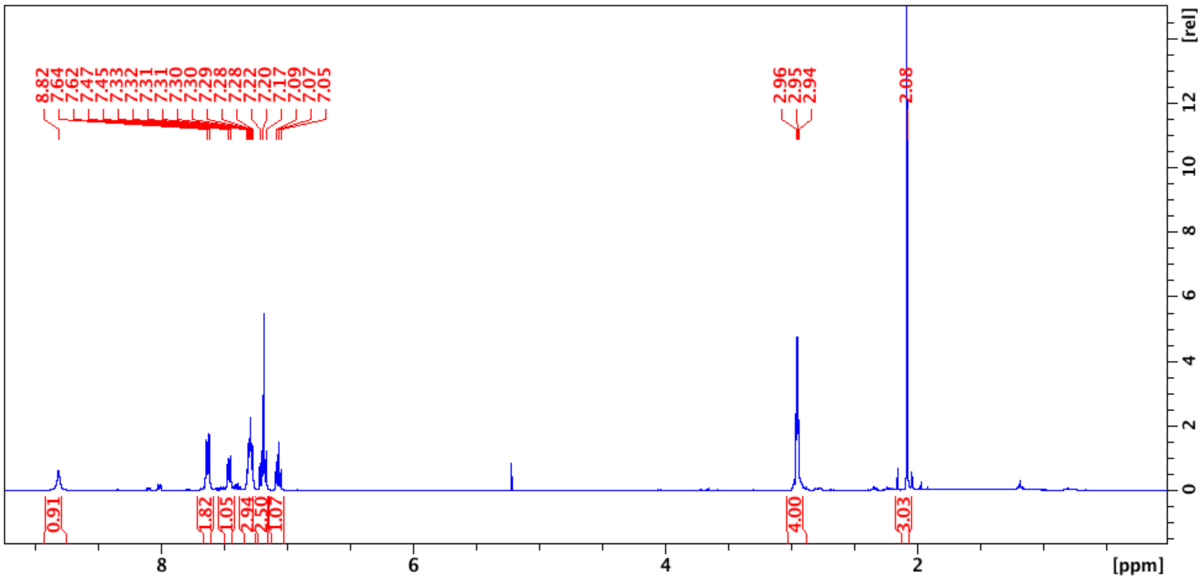

*Figure S47: ^{1}H NMR spectrum of compound **3q** in $CDCl_3$ at 298 K. Traces of unknown compound present, which are proposed to be alternative aromatic ring functionalization.*

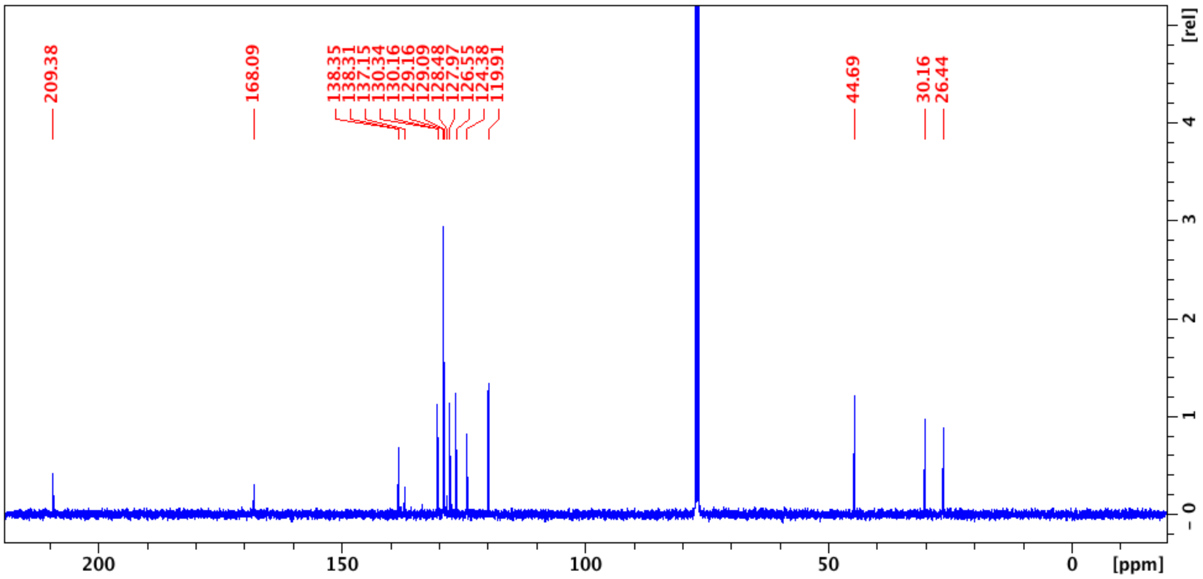

*Figure S48: $^{13}C\{^1H\}$ NMR spectrum of compound **3q** in $CDCl_3$ at 298 K.*

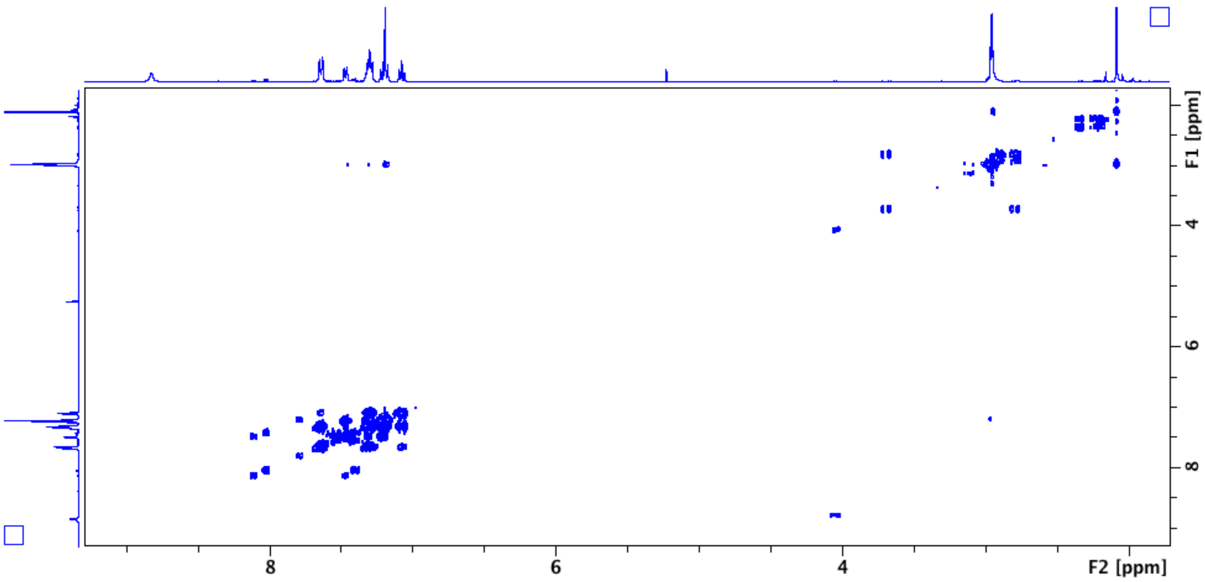

*Figure S49: COSY NMR spectrum of compound **3q** in $CDCl_3$ at 298 K.*

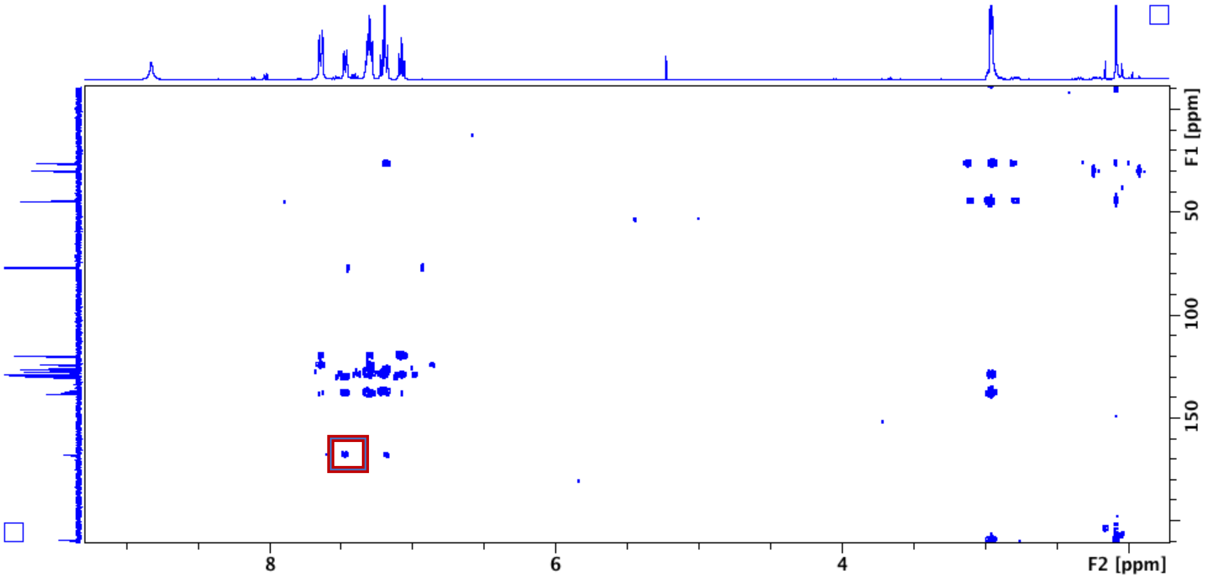

*Figure S50: HMBC NMR spectrum of compound **3q** in $CDCl_3$ at 298 K. Correlation between single aromatic proton and carbonyl of the benzamide highlighted.*

[5] Computational details:

Computational details: All DFT calculations undertaken using the ORCA 3.03 computational software.[3] Optimisations were performed at the RI-BP86-D3BJ/def2-TZVP level of theory[4-7] and single point energies and solvation corrections calculated at RIJCOSX-M06/def2-TZVP.[7-9] Frequencies calculations approximated the ZPE correction and entropic contributions to the free energy term as well as confirming all intermediate were true with no imaginary modes and all transition states had the correct critical frequency of decomposition (imaginary mode). Solvation correction was implemented with the COSMO[10] model for CH_2Cl_2. Graphical visualisation using Gabedit 2.4.8[11] and Avogadro 1.2.0[12] programs.

Calculated Structures

AcOH

C	0.07314062571764	0.39572173448082	0.08039171199520
O	1.24834503036206	0.36480072992513	0.38251116466215
O	-0.62346203314231	-0.72539872128957	-0.27658616421262
H	0.01984355239347	-1.46398387771840	-0.22399919101996
C	-0.78875934242156	1.63183919555068	0.04978177403677
H	-1.81693483017188	1.41431142021261	-0.25598148763634
H	-0.33864209041947	2.35714339224073	-0.64117089827268
H	-0.78398991231795	2.08722812659800	1.04910309044746

AcO^-

C	0.03598607385040	0.29685776359975	0.05511216778790
O	1.24642324127849	0.43030892687872	0.39046838938669

O	-0.59263483990739	-0.74820378331687	-0.27136091447641
C	-0.79308750064602	1.63367755651974	0.04924493905048
H	-1.83889776463871	1.47018902877139	-0.25380416156695
H	-0.32228645864540	2.35673393006308	-0.63766940877994
H	-0.77066303692124	2.08651073459101	1.05467090000639

ketone

C	1.57343543712572	-2.51205382504275	2.46839033020169
C	0.44820662757486	-3.17641642674301	2.18055536781546
H	1.56594448953168	-1.67041172015939	3.16699570150053
H	0.47901991532287	-4.01464776239209	1.48068847940661
H	-0.51052630725876	-2.90917765286697	2.62478734832061
C	2.88614943442465	-2.88881012018619	1.85507848199699
O	2.98754548046609	-3.80553891794395	1.04995844810694
C	4.08148631513075	-2.06864173830287	2.29405211769512
H	4.98559278502980	-2.40970484772349	1.77966970023948
H	4.21734535785612	-2.15442994450205	3.38433910664326
H	3.90908346479622	-1.00113504413723	2.08183391807329

Product

O	0.45193027060176	0.77162010864690	-4.90928429549663
C	-0.24964535890969	0.19813944317633	-4.07857675903456
N	-0.01209771007041	-1.07718623226093	-3.58958834193224
C	-1.51550936697133	0.82969612298734	-3.50487792401034
H	-1.71395169058746	0.51071152540964	-2.46338787940028
H	-2.38762991612555	0.53717462472279	-4.12651036935496
H	-1.41372180781324	1.92930623590408	-3.55614769894974
H	-0.63412001200059	-1.37730135824385	-2.82172362332136
C	1.06887754590527	-1.93435266176622	-3.85830037071558
C	2.20710627124495	-1.49522039795813	-4.57903659616938
C	3.28663700680852	-2.36025148743114	-4.79449161980131
C	3.25709089967738	-3.67732566373917	-4.30769618614177
C	2.11954369465706	-4.11906211009593	-3.61754677800031
C	1.01181470724672	-3.27912522667668	-3.38626154435329
H	2.20657107028131	-0.46918164395673	-4.96365751519852
H	4.16234029495760	-1.99570295573900	-5.35364616815887
H	4.10281727741505	-4.36016112198193	-4.47961324366389
H	2.07101763725896	-5.15986131053633	-3.25732069017850
C	-0.19557288568806	-3.85739730880556	-2.66760339360227
O	-1.09686493465688	-1.65107461180562	-1.04959375792459
C	-0.10661988713278	-3.84766092395723	-1.13102917930376
C	-0.50289982253295	-2.54563666091419	-0.45198328108856
H	0.90506434650662	-4.14903114332255	-0.78380113291984
H	-1.12992423563297	-3.34467791385452	-2.97683594043258
H	-0.30910798216034	-4.91148839465146	-2.99204121183996
H	-0.79445926619087	-4.61322773606444	-0.69976543916638
C	-0.15175652650329	-2.40625317644737	1.01366612440671
H	-0.30273400508750	-3.35433760763572	1.56812724600306
H	-0.73483154127565	-1.58670694349993	1.47137291466657
H	0.92948792677836	-2.15852146950246	1.08992665508314

iPr-benzamide

C	-3.13821301815115	-2.97055309700632	-0.55837356922019
N	-2.95182184174973	-1.53029621275897	-0.80613443721635
C	-2.07296955736953	-0.73389365835035	-0.12596036054088
O	-1.32006527642434	-1.15579551098839	0.75644972796873
C	-2.08117755908339	0.71914518645285	-0.52248354943748
C	-1.63777372402852	1.64883454250051	0.42867036701749
C	-1.61848508862599	3.01007344706662	0.12999721615357
C	-2.02474546247545	3.45723017273275	-1.13156509087905

C	-2.44722235592591	2.53576498780364	-2.09303296148590
C	-2.47818064779957	1.17340810457317	-1.78944719564795
H	-1.30798830661809	1.27336614991505	1.39750394243241
H	-1.28133581026148	3.72645525172958	0.88050875446221
H	-2.00275879913083	4.52195414269852	-1.36896021422422
H	-2.74449154259152	2.87790466709601	-3.08513850557296
H	-3.91099759443645	-3.27696197629526	-1.28161302464159
C	-3.66297704060144	-3.24044684322048	0.85764851771093
C	-1.85924426123588	-3.75702958052091	-0.86286441855652
H	-4.59360524165085	-2.68618067879549	1.04333722131947
H	-2.91775809568270	-2.93384933190022	1.60192637753493
H	-3.86959990048660	-4.31271759003693	0.98451768235797
H	-1.06095360200244	-3.46916959876130	-0.16838833593563
H	-1.51808467271752	-3.56398824157205	-1.88959310278355
H	-2.04944911802868	-4.83491633428004	-0.75781552655799
H	-2.77450367597961	0.46239117482073	-2.56293248776979
H	-3.63528980694235	-1.06606717290269	-1.39313002648764

[Cp*Co(III)OAc]$^+$

Co	0.98417452445472	-1.55154633671616	1.03382410819974
C	0.38348598124991	-1.08085806586443	2.88984959386938
C	0.53248269271206	-2.52652275011278	2.76210748463042
C	1.88000087763379	-2.78711244999296	2.38180959230683
C	2.57792251781877	-1.50377148767554	2.28675835464876
C	1.66039233192406	-0.45886736396026	2.62458919237865
C	1.92896840464291	1.00588785751504	2.63126032066688
H	2.84798019889235	1.25545955438671	2.08883667929965
H	2.04351253958611	1.36008853414174	3.66769267660681
H	1.10367275658335	1.57012580462784	2.17845364957831
C	3.98901173016382	-1.33517118292548	1.85679978689079
H	4.18767835406460	-0.32698408387611	1.47663825803326
H	4.27388210412669	-2.06738059527343	1.08999084289307
H	4.65401501467720	-1.50282609316472	2.72181279477845
C	2.48017744811512	-4.11342749506034	2.08043212592261
H	3.20439837354852	-4.05658328547986	1.25711770621035
H	1.71805159685779	-4.85763257100249	1.82321067537163
H	3.02422858868540	-4.48335141458388	2.96597220022162
C	-0.55578601507655	-3.52585565261090	2.93601916141632
H	-0.34499421329534	-4.45499259453577	2.39518258959504
H	-1.52252358247156	-3.13817606699802	2.59178092715557
H	-0.66244923841387	-3.77444862336219	4.00418547998189
C	-0.87594242099707	-0.36999143211076	3.21984221502902
H	-0.88375864416734	0.65497140710560	2.83180209519047
H	-0.97334265143006	-0.30698644129865	4.31826915738419
H	-1.76033184221468	-0.90222566146729	2.84884450827216
C	0.22463019664009	-1.30938180449407	-1.11466452675993
O	0.78220315317825	-0.34297204011753	-0.46293683158785
O	0.10537363830367	-2.41488916670002	-0.45374000815906
C	-0.26356882031229	-1.15755317325534	-2.50532170576319
H	-0.18484030111324	-2.10955139319407	-3.04397559795494
H	0.28109092633358	-0.35804050304729	-3.01955649840599
H	-1.32962822070075	-0.88039542889658	-2.46730300790091

Int A

C	-1.10780537372870	3.15025505965157	2.42368446860629
C	-1.40601854589647	3.33884585755402	1.03049504919633
C	-2.85165751985244	3.28862326729914	0.86461643249382
C	-3.43624945585070	3.13436729327006	2.17528056150014
C	-2.35939385071686	2.98719694737375	3.13680492432590
C	-2.49090487498655	2.84647004048446	4.61678255688107
H	-2.35539011148883	3.83785326710303	5.10156005886630

H	-1.71995995196221	2.16994246061335	5.03303315299794
C	0.24181677721301	3.08821763472462	3.05250952761300
H	0.42084866087718	4.01382522654290	3.64084234975888
H	1.04891615782204	3.00104054159679	2.30229840581717
C	-0.42868228080294	3.53737233744117	-0.07537975671016
H	-0.66147708653854	2.88590025794976	-0.93918622886128
H	0.60883450093429	3.33122723445437	0.24460674858644
H	-0.47223251412598	4.59139849993765	-0.42437659826170
C	-3.57589439746938	3.46485701479377	-0.42833204868586
H	-4.59668830949764	3.04225253811406	-0.39141923477377
H	-3.03069583019500	2.98753588338599	-1.26468156483673
H	-3.67197321198055	4.54542548355792	-0.66948987080843
C	-4.89096775991028	3.20137799144982	2.49765687517446
H	-5.13919982654057	4.24624460996151	2.78406618032188
H	-5.17385064566766	2.56029666992870	3.35364277433028
H	-5.53066643109896	2.94496902726080	1.63300196529961
Co	-2.21723121267754	1.51037941378482	1.68713574985446
H	0.32107119987697	2.23018852499173	3.74792693041403
H	-3.48605841628633	2.47137037053528	4.91767236236165
C	-3.47783696847915	0.17870141092479	2.69358947204125
C	-3.85899522758712	0.22023200549973	1.34889123320516
H	-2.83384362670045	-0.64971100739862	3.02921005686928
H	-4.04324590209553	0.71522890715489	3.46719697464339
H	-4.70365664701706	0.85032684683242	1.02417647157681
C	-3.45610217049410	-0.90049668402470	0.40896515220683
O	-2.73540724720150	-1.80902044424132	0.80249177832280
C	-3.99757636087328	-0.81547783743719	-0.99467541551893
H	-3.67405142365620	-1.69275929818945	-1.58280658368433
H	-3.61705782570307	0.11327471187365	-1.47002587150877
H	-5.10605766833577	-0.75265942695148	-0.98936879868276
C	-0.55344481514974	0.00096451951596	0.96124848190340
O	-0.75101869238066	0.28151043047630	2.19910915581732
O	-1.29557680947568	0.66221477536147	0.13882737316802
C	0.40926849845353	-1.03871655089068	0.51254970951852
H	0.78691901203749	-0.80979635150118	-0.50077369648748
H	-0.15299561895460	-1.99574923152468	0.46688363636154
H	1.23851680416353	-1.14816322924097	1.23497509878621

N-phenylacetamide complexes

Int 1

Co	2.12923352190813	-0.62856613633256	0.90875910157004
C	3.55546827198368	0.73234468122509	1.23864762596998
C	3.13510716474020	0.79349427860508	-0.15172373033448
C	1.72183040080124	1.06066603751765	-0.18109180240096
C	1.26488494559352	1.21230992790181	1.19675213708079
C	2.39335964823563	1.02163124167744	2.06714971324994
C	2.39309210185750	1.01091226561503	3.55894995719670
H	1.39033142171427	1.21795662643768	3.97235419365957
H	3.09396421514422	1.77732712279587	3.94999725876952
H	2.72289275482059	0.02303690372112	3.94071445733223
C	-0.13804438147013	1.53475119189681	1.58919817426827
H	-0.86896161177358	0.94807029584322	1.00005138047697
H	-0.34399239339953	2.60795243695154	1.38683798915956
C	0.83676682387860	1.14054018242012	-1.37966673056252
H	-0.08288667724173	0.54250368474209	-1.22708292130740
H	1.33971266806784	0.76514290010889	-2.28945672175169
H	0.53036171623563	2.19116154572804	-1.56691150670853
C	4.01994933882315	0.51299415257803	-1.31939045442642
H	3.45631748796549	0.48767106315739	-2.26929643098080
H	4.53308596602103	-0.46252129358705	-1.19937187710764

H	4.80206923902288	1.29700595356537	-1.40268890179666
C	4.93743195846157	0.46099404996682	1.73442148180206
H	4.91930646684830	-0.06421074678022	2.70792898962264
H	5.48941382249523	1.41508661028768	1.87656884497925
H	5.51455794442535	-0.15171277164614	1.01587768315203
C	3.26543415843317	-2.65696221644276	1.38921065666294
O	2.69772318558971	-1.95343118492222	2.29798004479405
O	3.18966291353406	-2.19281295105643	0.19413583955111
C	3.98487794831308	-3.92956302703818	1.71241038407073
H	3.50488797288730	-4.42848411490966	2.57477309625217
H	5.02914943226177	-3.68972337460684	2.00137083692546
H	4.01803812417519	-4.59616994232208	0.83090092163208
C	-0.01283649856979	-3.49664414069410	-0.99713775834690
H	-0.35280842408308	-4.54291077618958	-0.88433684854172
H	0.94580922028227	-3.47317050734668	-1.55409641169656
H	-0.75412176415161	-2.93728955283821	-1.60142630857410
N	0.04694095298696	-3.49431652426023	1.44553687098428
C	0.08433471421067	-3.04437702956334	2.79507044671947
C	0.49519340460202	-3.95743645183063	3.78494713906481
C	0.53447869510169	-3.55851094876206	5.12734434999631
C	0.15949811371600	-2.25273292209711	5.48658902555095
C	-0.27196517694796	-1.35582904550991	4.49526057625057
C	-0.31305882365041	-1.74119185905856	3.14837177579193
H	0.78926132877933	-4.98022870112123	3.50162571443046
H	0.85739758363035	-4.27399556764331	5.89775933188783
H	0.18708852820409	-1.94093320876956	6.54101440527221
H	-0.60190543292140	-0.34505154303730	4.77895468840295
H	-0.66450523917622	-1.04826888403906	2.37657437944218
H	-0.13326280700367	-4.49447312700086	1.32099985193449
H	-0.32320271301669	1.35668066656038	2.66352711139876
C	0.19050166546930	-2.77504436260169	0.30822746659326
O	0.47890212218477	-1.54672990729558	0.29554650263787

TS C-H

Co	-4.19015006081596	-4.43123885221201	3.29700828444732
C	-1.70199023454576	-5.88482986438286	3.73606482152220
O	-2.94466834648080	-5.93595165830140	3.55374326652453
C	-1.53020336350541	-3.42155182723153	4.07265173221288
C	-2.90867601492065	-3.23242820896558	4.38126243499212
C	-3.31114937464267	-1.91390964797973	4.71969456709161
C	-2.42383061876548	-0.83154008482396	4.69100163915213
C	-1.08224767037778	-1.04809613867625	4.32386892869845
C	-0.62925441352233	-2.34296034022175	4.02692156480699
H	-3.30299710809745	-4.08718508412487	5.23808233597027
H	-4.34821753286594	-1.75411115469392	5.05111341808140
H	-2.76513671339086	0.17676755367098	4.96778867448590
H	-0.37384474983945	-0.20699734620387	4.29179898058888
C	-5.98179905739661	-4.28779743554952	2.24557744063479
C	-5.29524810330097	-3.01554760489805	2.26729303601345
C	-4.00310922623747	-3.19897412490404	1.62400015519978
C	-3.88774766746858	-4.58144946001569	1.25914972696437
C	-5.10738324741488	-5.26864962052207	1.65847847076819
C	-5.38224679104784	-6.72687972069888	1.49457511859152
H	-4.51795586801849	-7.33375758940348	1.83111393303413
H	-5.57344974218161	-6.97579963424146	0.42938749916648
H	-6.26548450881031	-7.03985346377653	2.08120814503155
C	-2.72690412577683	-5.21570816301247	0.56624863088248
H	-2.64374027463296	-6.29213224384792	0.80637732624861
H	-1.77096939771769	-4.72047755466193	0.82256165136436
H	-2.85537591093050	-5.13169930585555	-0.53510493996811
C	-3.01928512922361	-2.12315495944298	1.30312601234914

H	-1.98324737529425	-2.50663306017978	1.26105991105082
H	-3.04902188788608	-1.29870109952021	2.03871191217900
H	-3.25570425110362	-1.69294714942935	0.30608730371508
C	-5.89327757743162	-1.71588660158854	2.70069740532910
H	-5.12554757185950	-0.93435705207665	2.84360648275871
H	-6.46285832448205	-1.81900776704510	3.64444293224005
H	-6.60460643207817	-1.35398199261568	1.92653482257815
C	-7.34304896575832	-4.55927741333742	2.78771258491603
H	-7.34252586535307	-5.46614863607278	3.42214677497342
H	-8.05167134475492	-4.72523847562279	1.94890285992466
H	-7.72224131232406	-3.71732518492166	3.39450796764295
H	0.43019439974942	-2.51249054362807	3.77525961263030
C	-4.78146526443658	-5.30773521049357	5.92853486294404
O	-5.26349482943646	-5.14090884697380	4.75017277208601
O	-3.63681237689451	-4.88787930481554	6.27933628223588
C	-5.66008599562228	-6.00391023109992	6.93988685502843
H	-5.04644431443207	-6.42898110509748	7.75389854532500
H	-6.26719087565281	-6.78837205085623	6.45211023451210
H	-6.35386696111328	-5.25873794046689	7.38141852468039
C	-0.92224699584473	-7.16855248207781	3.81813672106350
H	0.16536005610767	-7.02603125269695	3.67091468281761
H	-1.31119092548725	-7.88553230316836	3.07149311493729
H	-1.08525549660249	-7.61469667364416	4.82212004864538
N	-1.01089873371060	-4.72512463786665	3.88839278662549
H	0.00492447363057	-4.81557744972729	3.97245214430523

Int 2_{AcOH}

C	-1.34258205315657	0.28175910882690	-0.08405873656129
C	-0.20237263122768	0.65065422659596	0.72295222132682
C	-0.17196250661866	-0.21946136168968	1.90160912499111
C	-1.32740736382722	-1.03693696870596	1.86416481134759
C	-2.09191168368293	-0.70046464657604	0.65791189515300
C	-3.35178163577954	-1.39002374655260	0.24572957654928
H	-3.13368970578388	-2.43326388168508	-0.06899221421117
H	-3.84444035770804	-0.88618685114268	-0.60611237895534
C	-1.61748827738844	0.78356120635154	-1.46393198035683
H	-1.01662337328107	0.19580829434153	-2.18971999962814
H	-1.33787021976889	1.84790691157402	-1.58005634352995
C	0.85837272124497	1.63223565099910	0.33773226868724
H	1.39883976110191	2.02071181351805	1.22182934883510
H	0.43730928327515	2.48858274119237	-0.22293432583401
H	1.61486403396445	1.14800455130580	-0.31786986385077
C	0.86630698266865	-0.15040552294613	2.97185357246259
H	0.68983095995123	-0.89740103445911	3.76727990373602
H	0.86788833416780	0.85145821553077	3.44907839332038
H	1.87841923453954	-0.32673131342710	2.55308105314353
C	-1.77704641763249	-2.03580119232747	2.87733062618215
H	-1.85465156160666	-3.04414571373245	2.42018476707131
H	-2.78095408449327	-1.76785343475820	3.26418188983026
H	-1.08595582590890	-2.09961426869753	3.73776205810604
Co	-1.90530308322824	1.02485046961421	1.74925550320073
O	-4.30062283826576	2.65134667622486	3.28919815836690
O	-3.33287433687270	0.62062876141668	3.07293052027818
O	-1.11319635717435	2.29055323254731	3.01149450924356
C	-4.11968726817288	1.39819174345762	3.65627129197892
C	-0.73510947324865	3.46226321277426	2.73257675614554
N	-1.19875820349835	4.14872873289123	1.66630599183395
C	0.27973055691735	4.13092874120382	3.61981924505710
H	0.42499335150442	5.20220373250375	3.38857668487445
H	1.25256516039582	3.60642932555157	3.52373254475903
H	-0.04144060507857	4.02401092131933	4.67445029265119

C	-4.92011960733446	0.96065418479581	4.84406054416914
H	-4.82489495667465	-0.12769059962006	4.99550993583338
H	-5.98164739211584	1.25025218382444	4.71733600055031
H	-4.54542575310502	1.49106782346247	5.74365051129146
H	-0.83851354131673	5.09772543589539	1.54074248117231
C	-2.27553235391969	3.77417080786382	0.81757614940391
C	-2.82107512099630	4.78544132903787	-0.00346705005128
C	-3.94832576103488	4.51730267524060	-0.78845193793794
C	-4.54721974938477	3.24665628481957	-0.73227562383884
C	-3.98024141018260	2.24438138954873	0.07053100907835
C	-2.81507109145732	2.46111181791727	0.84484134428535
H	-2.37153248772824	5.79204593213497	-0.00822015656820
H	-4.37212964951483	5.30923919881724	-1.42273040518578
H	-5.45597615348035	3.03677448627598	-1.31556505135775
H	-4.46471898934972	1.25584793429303	0.10447789790874
H	-2.68069695816356	0.68201424545932	-1.74555427041913
H	-4.07175958284181	-1.44745216029183	1.08604330019960
H	-3.78420195772681	2.78667669748468	2.43534215526187

Int $2_{substrate}$

C	-0.15343320990823	2.03116693011574	2.38497193906062
C	-0.02057613942667	2.01326771539824	0.95531262681182
C	-0.88598864521298	3.04655549695283	0.40127988404709
C	-1.58099117329028	3.65635690622193	1.48593976792147
C	-1.18683823490130	2.98306644983178	2.71705706526503
C	-1.60334467429441	3.40837890227030	4.08803763597636
H	-1.01513126376199	4.29630707998812	4.40493337895299
H	-1.43055690537903	2.61907074351919	4.84281727806659
C	0.73999654097052	1.29871786053040	3.32983423829998
H	1.72287415386912	1.81700662781282	3.35231776236516
H	0.91819361228930	0.25500747098202	3.01237135916310
C	0.92013315728491	1.15347355167282	0.17804738341458
H	0.56543873074318	0.99619800939529	-0.85721599164457
H	1.05786690827596	0.16529957139320	0.65771808587766
H	1.92029674346477	1.63436024722690	0.11983483386579
C	-0.98462179272305	3.38365231596686	-1.04911619079755
H	-1.69963031629604	4.20599922017806	-1.23407704972665
H	-1.31220843971690	2.49606693407798	-1.62775325563521
H	0.00242244816314	3.69689223124447	-1.44753468883927
C	-2.44899753016617	4.87097455683244	1.42119637235545
H	-1.82431903370289	5.76923451008050	1.61649993840632
H	-3.24494299269120	4.86584967009427	2.19113497254379
H	-2.91704502518285	5.00488173345394	0.42867329961911
Co	-1.99650960394858	1.53575157557740	1.42009401232383
O	-2.11064372896165	0.65834006895879	-0.31394507658098
C	-2.18397889652346	-0.57003283563975	-0.60101494472839
N	-2.00351773584289	-1.54265877189672	0.30295167653018
C	-2.52959781935182	-0.95920527940917	-2.00919110714510
H	-2.26150813734046	-2.00550945257820	-2.24683075414714
H	-2.03079197445291	-0.27656834309326	-2.72066306718391
H	-3.62785419904217	-0.82962132592063	-2.11866707163294
H	-2.08871869943115	-2.50223601561794	-0.04088975976121
C	-1.81681420762364	-1.41416718530570	1.70249953264040
C	-1.61677017933285	-2.63011718579027	2.39788038915323
C	-1.51486060020891	-2.64979986011488	3.79037627564207
C	-1.63006163637299	-1.44149973017247	4.49509134421068
C	-1.80979264482145	-0.24094332535592	3.79262689586512
C	-1.87160035694551	-0.17587246453084	2.38455244366287
H	-1.55457597529868	-3.57062091874848	1.82592864583242
H	-1.36591487838414	-3.60213209392112	4.31906032538383
H	-1.58908159352063	-1.42928473281205	5.59476124595801

H	-1.92285336036784	0.68257170706727	4.37614211067104
H	0.34766945856549	1.28034287320663	4.36120048886971
H	-2.67126787032742	3.69765945077315	4.12797659423588
C	-3.88698068266653	1.26760161870160	2.30469218441829
C	-4.03797763753769	2.07796106187669	1.17171164071870
H	-4.25209692563958	0.23232937205987	2.24159999406146
H	-3.83872314414739	1.70203322420205	3.31376594874720
H	-4.08660615323928	3.16963055518073	1.27115573410089
C	-4.63152515135428	1.50802946078192	-0.08961715574073
O	-4.89424845388249	0.31295126644812	-0.18875903543549
C	-4.87270901786889	2.49297642186853	-1.21027452464710
H	-5.37652946750543	1.99894447226738	-2.06013324707367
H	-3.89785385232325	2.90593359128480	-1.54498569302882
H	-5.48223479270842	3.35249906541236	-0.86170269128998

TS C-C

C	-0.09471517139806	2.06348990189221	2.34619099253970
C	0.05745883288991	2.07900928318748	0.92715437798529
C	-0.88165404937782	3.05136807313982	0.37256685371959
C	-1.61410587381010	3.61850377778031	1.46339089533297
C	-1.18207506884843	2.96345755396097	2.68493923879440
C	-1.63950483266930	3.34998520173397	4.05671985711363
H	-1.30392844643734	4.38233937784298	4.29245157765769
H	-1.22493828694432	2.68702809913010	4.83745719371352
C	0.78319223498533	1.30780686195106	3.28791613887520
H	1.78534993394283	1.78606332442056	3.31105336123810
H	0.92334976331802	0.25671818374212	2.97079034533709
C	1.04162689848377	1.27795444023888	0.14068976622289
H	0.67139748047258	1.06810973545536	-0.87968588911549
H	1.27041497555968	0.31514500468141	0.63608124788879
H	1.99778400467206	1.83561425400062	0.04258824587323
C	-0.99292230531612	3.39133116157088	-1.07671230907648
H	-1.74042603189531	4.18514541448537	-1.25937242712435
H	-1.28001379915717	2.49450134380807	-1.66285017196830
H	-0.01870881012504	3.74872834444747	-1.46977707345687
C	-2.55828215916609	4.77567836337225	1.40159521978754
H	-1.98320891629394	5.71859252679543	1.52504572258147
H	-3.30992627083208	4.74911391602854	2.21381101511986
H	-3.09109324660006	4.83671535379994	0.43541659769117
Co	-1.92038068360518	1.52759656599031	1.34877999948096
O	-1.91933811183416	0.56331885128291	-0.34742980064074
C	-2.12386443214561	-0.66092735402756	-0.56808258962607
N	-2.08912113619705	-1.59022355441059	0.40660438895240
C	-2.42553415726317	-1.10730260701136	-1.96761504298334
H	-2.30895435063075	-2.19765886020624	-2.11122321604163
H	-1.77290373526881	-0.56398408282792	-2.67511383875537
H	-3.47493556951621	-0.81300721134044	-2.17848130465275
H	-2.23425073097729	-2.55972014953011	0.11467864689548
C	-1.82038008309927	-1.39699839955254	1.78454179632620
C	-1.39918497374817	-2.53758038793922	2.49934030283478
C	-1.19701230554185	-2.48349188600040	3.88280275931550
C	-1.43227519385174	-1.27492165677907	4.55818046759033
C	-1.83747271795418	-0.14372505463314	3.83974604488341
C	-2.01608233382365	-0.14492406469673	2.43427164742858
H	-1.23548858537329	-3.48066390586567	1.95362895902450
H	-0.87719448074841	-3.38313538789032	4.42759750368166
H	-1.31180247531239	-1.21279278171840	5.65004080543248
H	-2.05577061688282	0.77775581884999	4.39650611095479
H	0.38898926898810	1.29985779027448	4.31861110236951
H	-2.74420911201145	3.34232107114164	4.14564684230287
C	-3.75916163369507	0.77143480081616	2.08504198092989

C	-3.91651071346194	1.87568689137487	1.17518446566540
H	-4.17226398559381	-0.17659410415309	1.70107829175642
H	-3.98187600743870	0.95844927090463	3.14521714351341
H	-4.11571177335781	2.87183072927536	1.59371412294140
C	-4.50186651067029	1.61439195256522	-0.17600712226383
O	-4.69323012110142	0.46502599100526	-0.57533537229491
C	-4.84254666236529	2.81670204977230	-1.02930623686203
H	-5.48121282286025	2.51173195058393	-1.87762976165371
H	-3.90083158069222	3.24468260890758	-1.43494939385840
H	-5.33895052741885	3.61600660837236	-0.44376347937785

Int 3_{ketone}

C	-0.00101115044135	1.85497405180140	2.28387684980511
C	0.05665241416734	2.00877852388726	0.87366219270463
C	-1.01608664952196	2.91908469961981	0.47622548115430
C	-1.70356616579059	3.35482159539487	1.67558281140179
C	-1.12191900055198	2.65305783279824	2.78650237371065
C	-1.46830577734181	2.87791345441061	4.22304694390126
H	-0.97766483386344	3.80775955053764	4.58425197686271
H	-1.12219895364924	2.05166139717950	4.86954583560969
C	0.97114071092778	1.08406414343650	3.11316013231871
H	1.90753128562558	1.67220206132227	3.22280249793693
H	1.24229527721428	0.11797867162335	2.64690012554571
C	1.02797902618363	1.36741107165512	-0.06193166278190
H	0.54873268342128	1.10591907383112	-1.02429226544270
H	1.46529372640542	0.44674867442027	0.36767728654331
H	1.86515142126230	2.06266523591807	-0.28576435041014
C	-1.25947772333669	3.37177839505667	-0.92436684990222
H	-2.11643647543026	4.06633791509118	-0.99493104045144
H	-1.45011130948667	2.50701393612386	-1.59063118121692
H	-0.36296324172772	3.89939895432982	-1.31410305689102
C	-2.73565770081058	4.42886302755903	1.76444827724362
H	-2.23313835406022	5.40256711463545	1.95013526847713
H	-3.44364456788428	4.26434065774615	2.59876022530929
H	-3.31808069055130	4.52936914028073	0.83210413556768
Co	-1.86548495465142	1.32826476923162	1.37981678185137
O	-1.91954683250192	0.31590546905216	-0.27826187301920
C	-2.19507297744345	-0.90354052441090	-0.42412191630081
N	-2.17941627676869	-1.74904350440988	0.63436572099493
C	-2.52770855943449	-1.44512141478959	-1.77977963303817
H	-2.44752187611069	-2.54674414578277	-1.84059348313460
H	-1.86931236972353	-0.97642999546015	-2.53373139962082
H	-3.57018129531085	-1.13411769153631	-1.99728960605487
H	-2.31240604524838	-2.74681724414890	0.45029200134710
C	-1.84218733474394	-1.36817423882155	1.97045869349154
C	-0.73469108664295	-2.00628895270451	2.57121398559804
C	-0.45733819691040	-1.81049322653852	3.92700173786308
C	-1.27215080352594	-0.95182133293955	4.69331465712985
C	-2.35501186575040	-0.30530376810778	4.09528001403447
C	-2.69057390591581	-0.50393138469338	2.72790842852123
H	-0.10740346673904	-2.67370289382795	1.96158126396370
H	0.39433138272997	-2.32849893951462	4.39218943039648
H	-1.06785188803782	-0.80774591484430	5.76480445198377
H	-3.01784292277628	0.32998320794600	4.70156635115513
H	0.58621000609697	0.87609455680996	4.12591490968592
H	-2.55802456624275	3.00484516417186	4.37144272174889
C	-4.02699044021935	-0.00724796988308	2.19107107590190
C	-3.88853665282077	1.32207694121873	1.45064135365106
H	-4.43787258373473	-0.75451011818493	1.48076809564648
H	-4.73176002243411	0.08395429589574	3.04202150901577
H	-4.18695304193577	2.20060487009935	2.05027817662051

C	-4.52336550947304	1.31470778962470	0.09804516270938
O	-4.73486270445573	0.25584569817585	-0.50265404341302
C	-4.91106428854557	2.63689389632729	-0.53646364141613
H	-5.61662183230463	2.45263410079365	-1.36693109676312
H	-4.00677133377883	3.12020453650884	-0.95955394204477
H	-5.35779070540513	3.33808078608402	0.19584510449876

Int3$_{enol}$

C	0.05547061696052	-1.66889130340108	-1.34696705466720
C	1.38285177522133	-1.21819721894544	-1.66614053108728
C	2.25083128844004	-1.48838571117861	-0.52928262017486
C	1.45591356632651	-2.14972057703403	0.48228812743037
C	0.09650341356108	-2.21347856388694	-0.00147751267226
C	-1.05129427650786	-2.79901889023622	0.74633704100545
H	-1.06949931180398	-3.90169671182630	0.60093854753559
H	-2.01527499798443	-2.39127262206516	0.39587572716951
C	-1.11396682360097	-1.66674465480337	-2.27705933171742
H	-0.99689226694027	-2.46690756434895	-3.03944399729524
H	-1.21144559407841	-0.70811008882040	-2.82507505677096
C	1.81459814201652	-0.57656117641724	-2.94302520308122
H	2.51680896298039	0.25945144353785	-2.76067248924013
H	0.95459589490882	-0.19393803091063	-3.52407144091909
H	2.34019679874786	-1.32119058853852	-3.57911761085498
C	3.70538919206318	-1.16933838790766	-0.43756752394185
H	4.03426037961106	-1.07724120634486	0.61366433194852
H	3.95274016676399	-0.22667984411604	-0.96259149632608
H	4.30480353581895	-1.97677871062404	-0.91263384726986
C	1.93768076262041	-2.56278916922631	1.83150874584189
H	2.93304187655030	-3.04412189604337	1.76805165189935
H	1.24102210104195	-3.26945833667098	2.31996021057106
H	2.02922122364627	-1.65915481044257	2.47424586462628
Co	0.75401734523766	-0.26469195701004	0.02540045982470
O	0.79167694845236	1.45733652322853	-0.89617976603965
C	-0.10160678668700	2.06886635938954	-1.53751509857436
N	-1.42592412932854	1.90620363666013	-1.28236584230426
C	0.29445334961363	3.04173907715563	-2.61464234493113
H	-0.57044241650986	3.47699092662737	-3.14760301435772
H	0.96637695783124	2.54235073904934	-3.33905324189391
H	0.87617718236115	3.86373695061731	-2.14983825317777
H	-2.09105355447698	2.36808775964335	-1.90783607377719
C	-1.94087726373638	1.20937692091712	-0.14815892171947
C	-3.00197540924144	0.29781291489794	-0.32673149184204
C	-3.53882106296507	-0.37304041541474	0.77659940057979
C	-2.98749060529830	-0.16111184438918	2.05578831087723
C	-1.96201740956334	0.77372528690719	2.23095855451392
C	-1.45079013425187	1.52138859904328	1.14654780234506
H	-3.40585541792043	0.13041847453248	-1.33546797287207
H	-4.38354551326380	-1.06428456721744	0.64077811362866
H	-3.38989225133774	-0.70182312558176	2.92488270815929
H	-1.57253453778803	0.98371572824815	3.23877065983567
H	-2.06029342933445	-1.85561958495691	-1.74025845367877
H	-0.97337680455897	-2.60361322015588	1.83269785423717
C	-0.62163421721375	2.76010496349352	1.41572319742138
O	1.24577106242100	0.32574106236251	1.67925086490009
C	0.71737794625048	2.63157245500505	2.07620614079099
C	1.52769317276079	1.53124260794513	2.16708311396456
H	-1.25175048534334	3.39860697619882	2.07716246013176
H	1.08034990215749	3.54525836904363	2.57091519730153
H	-0.52470557882657	3.36567315927441	0.48821284495966
C	2.84212583716162	1.57542052822845	2.91266847323809
H	3.04372400578205	2.57172489618767	3.34795202294054

H	3.67422680048669	1.31516782733389	2.22577227832223
H	2.84554707076642	0.82468559298641	3.73043348518640

Int3$_{enol}$ + AcOH

C	-0.00187436749913	1.98494473051639	0.38433150312548
C	-0.03340180991398	1.69953779062508	1.80136773952547
C	1.33952354936494	1.60135490214802	2.24821822607594
C	2.21154026110336	1.79250886799217	1.10992824683133
C	1.37608595290925	2.04786311426983	-0.04841110852012
C	1.89028899305632	2.26781098256258	-1.42845481845719
H	2.64963744642826	3.07662231045287	-1.43518350924910
H	1.08682019462887	2.53353511378737	-2.13740830416688
C	-1.18802757181361	2.18006563866740	-0.49709118489349
H	-1.42904698217273	3.26308768409699	-0.55720238296119
H	-2.08342328031493	1.66219581690359	-0.10755412610550
C	-1.25189948832496	1.52430010075608	2.64692777797027
H	-1.06357327726688	0.82306744931143	3.48210952387103
H	-2.09926386578754	1.12882277475603	2.05568707381927
H	-1.57606478251843	2.49142515134183	3.08717191566892
C	1.78505837795060	1.31567248352019	3.64039995998136
H	2.72534623728051	0.73365637148539	3.65879484278820
H	1.02030903776101	0.75862867265069	4.21048362495703
H	1.97347550617213	2.27579914123008	4.16771449510332
C	3.70208340313540	1.75514931511581	1.09084827171298
H	4.11532905349163	1.25306737880452	1.98465336722125
H	4.11308447839570	2.78705279156202	1.06260770585106
H	4.05928400221637	1.21705724973513	0.19233499714460
Co	0.98143658260682	0.22421509693537	0.77243968698769
O	-0.61086421589169	-0.87012136805996	0.11943914600488
C	-1.48933243078925	-1.20236597098345	-0.70649342840440
N	-1.36848456011485	-1.10178333237510	-2.05220809557343
C	-2.77992650031073	-1.79777187423498	-0.18737394641729
H	-3.52630363516071	-2.00818849804563	-0.97564220177790
H	-3.21897999573878	-1.11387278964527	0.56504610523440
H	-2.53559825705336	-2.74466600685748	0.33533216897511
H	-2.19399525559254	-1.36446250072939	-2.59775178455536
C	-0.21110937473563	-0.69352156171965	-2.79719421264700
C	-0.32769345515245	0.43422329471162	-3.63478727053197
C	0.75493438094891	0.84113685943597	-4.42350262273263
C	1.96078242377819	0.11943909372345	-4.36084259948249
C	2.05844979682298	-1.01544850503268	-3.54677342694125
C	0.97411504903585	-1.46194273179461	-2.76160765437602
H	-1.28386950038182	0.97839290788655	-3.67541990774182
H	0.65925015665468	1.71703179707212	-5.08186655629135
H	2.82307426687537	0.43261032452212	-4.96821576699017
H	2.99497117981553	-1.59388182499556	-3.52326518290092
H	-0.98553596776024	1.82702911120744	-1.52464954626577
H	2.37789030847311	1.33898715793697	-1.79356290125609
C	1.07097396391881	-2.78644846945254	-2.03257376057365
O	2.21192256563606	-0.60192719850224	-0.41825672326906
C	2.13409995533814	-2.89500918949533	-0.96251472530187
C	2.64607991701471	-1.83842337676028	-0.24085591810750
H	2.60587707057720	-3.88226617573700	-0.83370515851973
H	0.07350794160998	-3.07106778843681	-1.63337370248407
H	1.28792569800337	-3.56296333445334	-2.79773000143527
C	0.50294005206473	-2.31497261419205	2.36195636800894
O	0.96378480110778	-1.15778308238083	2.26075011596525
O	0.54704502217256	-3.22545191640318	1.41741763820519
H	1.10482964384060	-2.91286082315886	0.61085228413189
C	-0.18359837564688	-2.75540806992486	3.62456262985028
H	-0.14282473260341	-3.85282961606673	3.74675451314766

H	-1.24916560546457	-2.44862231819552	3.56404321063910
H	0.26401487640150	-2.24480708647608	4.49602414691225
C	3.76790139727679	-2.02846862693513	0.75675807234650
H	4.03676898976744	-3.09062334792687	0.90535952074543
H	3.48530728400853	-1.58574473682097	1.73366655360398
H	4.66785347036506	-1.48408373993076	0.40338009652439

TS C-H

C	0.05221133056136	2.18996540555858	0.46110673080873
C	0.06086533797742	1.82985075149967	1.86454696083260
C	1.44349220172756	1.61754402197487	2.25001907709186
C	2.27010806371407	1.78281770113151	1.07919897616512
C	1.40521315575749	2.14951113309447	-0.03218142737342
C	1.87685043990051	2.40844429789206	-1.42343656607528
H	2.68844839068415	3.16526052135785	-1.41998308568997
H	1.06388261668465	2.77648127411570	-2.07398833846796
C	-1.15652211378368	2.50062837365887	-0.35377144237599
H	-1.33535938884747	3.59752480953892	-0.34903434057478
H	-2.05901981273946	2.00904548289239	0.05219152388133
C	-1.12551183795822	1.70732546515322	2.76114016992057
H	-0.92927464454799	1.00024387556300	3.58857694902393
H	-2.01316634439636	1.34955476105858	2.20609680124307
H	-1.38523664744009	2.68992657921352	3.21021037150672
C	1.89269789579644	1.21649102698481	3.61257242582152
H	2.98109306842414	1.02782638356928	3.65395909516443
H	1.36654867522400	0.29245447597624	3.92575574577978
H	1.64924475071111	2.01564119690442	4.34331667151157
C	3.75436949354121	1.67171277054319	0.99067333046305
H	4.18245519954190	1.09813210455054	1.83318124659240
H	4.20848044537814	2.68591450955312	1.01122059474055
H	4.05658479048354	1.18815369553706	0.04323053696123
Co	0.88837215142696	0.33228726455131	0.74860227884009
O	-0.83751277106471	-0.47311598902911	0.06243062450133
C	-1.49666170251224	-1.24125611905460	-0.67339998963394
N	-1.22860086473717	-1.47818165178667	-1.97771880608517
C	-2.67251315201500	-1.98901595054108	-0.08712657932896
H	-3.38275039156558	-2.36071008837450	-0.84926557675299
H	-3.20378130642666	-1.33425645985783	0.62784326948846
H	-2.26515886154284	-2.85330585386216	0.47661175808903
H	-1.87513598057044	-2.10708624326604	-2.46323426415700
C	-0.13994167560373	-0.94253538370409	-2.74926634893803
C	-0.33471193308315	0.25983992826412	-3.45057662530128
C	0.69255563100354	0.77810087461471	-4.25139007413605
C	1.90908245828755	0.08064699283719	-4.34889180074856
C	2.08807593193674	-1.12478178018770	-3.65597573232635
C	1.07079382516819	-1.66324885284424	-2.84241440015620
H	-1.30610832360838	0.77144156999189	-3.37612310174742
H	0.54115755462620	1.71562563797940	-4.80623821419748
H	2.71965732726285	0.47231798909726	-4.98168297961734
H	3.03961180834361	-1.67159132722925	-3.74627127582467
H	-1.02538808171183	2.18347703383876	-1.40500349418253
H	2.27789571323520	1.47957515026615	-1.87737589162534
C	1.27311615550557	-2.98700945791056	-2.13463969616540
O	1.98490735091426	-0.64573672854200	-0.50095159602146
C	2.12518139105138	-2.95493151319171	-0.86323902826601
C	2.56009753163664	-1.76659688701109	-0.23513344598859
H	2.74659896833972	-3.84972300954647	-0.68836030886318
H	0.28636581345431	-3.44373746940576	-1.91004491620532
H	1.76502585736191	-3.68461275566397	-2.84407736447799
C	0.26136114200473	-2.28120487939677	2.07582716506899
O	0.77863543446037	-1.11523834499522	2.10954861042477

O	0.28033278591818	-3.09600099886820	1.08835234374993
H	1.19696544433451	-2.96591335442704	0.19704768327590
C	-0.46740358525782	-2.73474493281293	3.32199396294637
H	-0.58875182539867	-3.83233287539895	3.33730924882299
H	-1.47126899096123	-2.26184686712216	3.32911885178127
H	0.06328863774801	-2.38715374941767	4.22833243878411
C	3.60496798755016	-1.83071060095653	0.85400599231796
H	3.87182787793958	-2.86820932744714	1.12494155789350
H	3.24807832962701	-1.29367812139263	1.75312416906358
H	4.52224727052789	-1.32218648551859	0.49294854874795

Int 5

C	1.80108951440924	1.83992161809646	-0.53432396563899
C	1.45672558386358	1.86101522094534	0.87317798495581
C	2.08077836961006	0.70152393669243	1.50039551166785
C	2.76832880971504	-0.03939649576243	0.47782537125060
C	2.57411720336218	0.64703133104777	-0.79223123546535
C	3.11451853737363	0.21348058514353	-2.10963985747366
H	4.10288358608353	0.69069261977972	-2.28766485591527
H	2.43439963639635	0.50344368957710	-2.93371034986705
C	1.36514606227733	2.81730729640147	-1.56730415810843
H	2.23741226528502	3.43881551041739	-1.86601504529996
H	0.57640514725012	3.49558115475467	-1.19382078309465
C	0.63232403552316	2.89227102252579	1.57026997476983
H	0.08585036646060	2.46131360624545	2.42983311216308
H	-0.10451605940811	3.34829144421199	0.88243704153369
H	1.27873311504442	3.70942388541628	1.95610367901200
C	1.95187465049712	0.34643269128309	2.94391526468752
H	2.35098756040123	-0.66089415521111	3.16240801787686
H	0.89159058883251	0.37902646630877	3.26480784745807
H	2.51107355447861	1.07450315113099	3.56808695006360
C	3.58384485536713	-1.27998962703291	0.64286421372750
H	3.48523714481938	-1.72325881474637	1.65139047807475
H	4.65744904393157	-1.03490249378429	0.49334493345327
H	3.32686358306307	-2.04588980310326	-0.11556175792637
Co	0.76556103665413	0.16625504364799	0.04704845346848
O	0.62763811165592	0.91851253204134	-4.00237669180328
C	-0.38344419019758	0.21920401358823	-4.10893995697796
N	-0.32771360591850	-1.15615289749742	-4.21503175011264
C	-1.77692127565900	0.81235379864026	-4.08258047793522
H	-2.57433843945926	0.10468197106337	-4.38134577561272
H	-1.80553226791409	1.69934601438827	-4.74292216136301
H	-1.96190920398938	1.15521064668719	-3.04469553443080
H	-1.21946563873530	-1.64723706565779	-4.28403341781324
C	0.85129982320212	-1.94100511975707	-4.11264441437579
C	2.00725286780676	-1.56359071307266	-4.82643447334942
C	3.18383060497014	-2.31481502305141	-4.71471847027217
C	3.20982993869340	-3.46074841958042	-3.90270022834135
C	2.05291263352520	-3.84372037306075	-3.20772360082361
C	0.86191300788577	-3.09395060280272	-3.28550383765011
H	1.97137159462132	-0.66969977296180	-5.46158496609038
H	4.07951129996968	-2.01153525071119	-5.27673711102705
H	4.12458126263949	-4.06713403262429	-3.82471296322334
H	2.07630541758695	-4.75898337771484	-2.59519941972477
H	1.00225029226199	2.30559255001498	-2.48435807569907
H	3.25868226127749	-0.88269038770533	-2.15614871313106
C	-0.37322699402781	-3.52579062148084	-2.51641317380483
O	0.45974595282733	-1.58906011852740	-0.73354719559905
C	-0.12763933716056	-3.86305043468037	-1.04322468538362
C	0.27187420433306	-2.69435192938348	-0.18617360262088
H	0.64733461294761	-4.65227204338988	-0.90550043510513

H	-1.13499017454518	-2.72227586709167	-2.55100914609195
H	-0.82773542348677	-4.41586139833741	-3.00249189007381
C	-1.59478367720641	0.38236058672150	0.26331932824882
O	-0.86477268568571	-0.08604485028633	1.21317993878484
O	-0.97612804573074	0.74140446468766	-0.79768694891531
H	-1.03712147684326	-4.30511090923460	-0.57471543179465
C	-3.07846563456453	0.53237997593197	0.41024212794040
H	-3.57867747087736	0.46943937107942	-0.57336292862879
H	-3.29511962811617	1.53359170588035	0.83834458513554
H	-3.48151463518768	-0.22654558660732	1.10577019315046
C	0.39202564390724	-2.88289376838820	1.29556045596515
H	0.27807921246105	-3.93746441559040	1.60256265430401
H	-0.38309801475529	-2.25436548469848	1.78346316900722
H	1.36647888619713	-2.49234005081634	1.64414519986538

Benzamide complexes

Int 2_{AcOH}

C	1.40112879825708	-2.95537476173215	1.50124034020774
Co	-2.35486362647622	-5.43954451803007	3.10452811697216
N	1.34781012869755	-4.37311121363035	1.90317297416855
C	0.23119330227481	-5.00826446912652	2.28467305646614
O	-0.89270590051221	-4.39798422792653	2.28795810149865
C	0.21698126999375	-6.43253801313870	2.66791804542402
C	-1.10179740816719	-6.88918385863800	2.98540969222553
C	-1.26620655817022	-8.27204645468342	3.21470367319098
C	-0.16679854656778	-9.14859572689534	3.18364931105504
C	1.13034115792987	-8.66827338651291	2.91603880457347
C	1.32196007723643	-7.30807002954492	2.64217126587119
H	-1.44522527748439	-7.04459459429678	1.12578895682792
H	-2.26862233782015	-8.68147290597172	3.41303875645106
H	-0.32201547023083	-10.22279072327468	3.36952028185081
H	1.98475951494851	-9.36024767585948	2.90117603744438
H	0.37905508125813	-2.71654217711426	1.14284531761454
C	2.40023718008761	-2.79333592455533	0.35403568863927
C	1.72284950924870	-2.06364110825591	2.70763594333146
H	2.13058823740449	-3.43055742052213	-0.51087118444423
H	3.43019600768662	-3.05651257161404	0.67736241090803
H	2.42300794903653	-1.74067964190298	0.01234485892045
H	2.72932644798979	-2.29098065478359	3.11618515164592
H	0.97838162837014	-2.20480631851801	3.51678113075340
H	1.70988265572848	-0.99584123236435	2.41199481672074
C	-4.12162631495413	-4.45922158202966	3.81650995841551
C	-3.98349650530910	-5.84406722682942	4.27825163115991
C	-2.77258316335482	-5.93285937008772	5.05482279216853
C	-2.09264056246365	-4.66239184825867	4.94546197788576
C	-2.96807450211278	-3.73911895001179	4.21844079380084
C	-2.61717608948051	-2.32144421499950	3.90631355385476
H	-1.64914217747027	-2.27649476091304	3.36743199630112
H	-2.51373937481246	-1.72921517096184	4.83904498497394
H	-3.38142909959262	-1.83369028392166	3.27348931338467
C	-0.77886255697986	-4.31898897120440	5.56744161377669
H	-0.27732309990054	-3.49568704679996	5.02510164992644
H	-0.09830415594547	-5.19264680950960	5.58034117309780
H	-0.91703683031277	-3.98935916021534	6.61971522857847
C	-2.33445155014370	-7.07304824525054	5.91434244016366
H	-1.23706724110827	-7.20897387662670	5.90470567561721
H	-2.80229312608622	-8.02933516376368	5.61940034368177
H	-2.64113085735393	-6.86730963877436	6.96270261111568
C	-5.00233519954817	-6.91849521916473	4.07810076766634
H	-4.57720560285829	-7.92495812181322	4.25407861075704

H	-5.41910944436309	-6.89433297633131	3.05306198570259
H	-5.85222637615596	-6.78857882262918	4.78281504495013
C	-5.26363098131796	-3.96608382228335	2.99217662701286
H	-5.13434343172296	-2.90955363162668	2.69418721164345
H	-6.21517060036956	-4.05028293208949	3.55750431517885
H	-5.36608136431394	-4.57146053777894	2.06961380670620
H	2.33362966734515	-6.94436851006786	2.39715596621504
H	2.22915997776317	-4.89130248442631	1.93669876970664
C	-2.89722641392772	-6.17693096853825	0.29520585507719
O	-3.25724751652753	-5.62488766764527	1.35652992786271
O	-1.83738369476689	-6.95668134470717	0.20548894229925
C	-3.65778575670628	-5.99471172655961	-0.98266420006588
H	-3.00795020332132	-5.48675795675375	-1.72451672370369
H	-4.56597347099984	-5.39383532407750	-0.81027161225130
H	-3.92066920154719	-6.98328202445738	-1.40803058097739

Int $2_{substrate}$

C	-3.19276531911706	-2.72914726122106	-0.04513249975214
Co	-0.27339259522789	1.06168982387514	0.16037607051832
N	-1.73079688552745	-2.68367389294170	-0.22458775346960
C	-0.98397024350098	-1.57747875839824	-0.16109989485181
O	-1.53715056494272	-0.42114766980914	-0.09051815672261
C	0.48082957930037	-1.61310571635381	-0.15604608309277
C	1.06406078466982	-0.34186066807822	0.08762234716588
C	2.46222486468650	-0.26691738704107	0.20591891113407
C	3.25754162135429	-1.41113902387813	0.03017148135569
C	2.67157571195350	-2.66118404960400	-0.25227558123884
C	1.28013195817875	-2.76417213761839	-0.33320310752429
H	2.95265123869024	0.68598842317840	0.45534728419785
H	4.35181494871740	-1.32885057978143	0.12225248300198
H	3.30194402644938	-3.55055748576926	-0.39469210116210
H	-3.50834448475892	-1.66820897834930	-0.00946297664917
C	-3.51621824752618	-3.40036661895089	1.29587490294017
C	-3.84949229695653	-3.41875999695703	-1.24551376950901
H	-2.98669757501543	-2.88574877556746	2.12173045428200
H	-3.20849056011765	-4.46735360522549	1.29002102314487
H	-4.60719287879583	-3.37082811694009	1.48812295429900
H	-3.51320210822767	-4.47317964957569	-1.33442390661999
H	-3.60769270534554	-2.89863756518882	-2.19369473344206
H	-4.95084099728488	-3.43066209684330	-1.12520920207064
C	-0.59605187015430	3.14767491458347	-0.08727403603345
C	0.74984655253634	2.76929548898976	-0.48836473761475
C	0.63927934545623	1.84155865110846	-1.59013876121704
C	-0.75947391359186	1.58776498533204	-1.80537234020154
C	-1.52915779435241	2.39799657300153	-0.87373935065452
C	-3.02117769747589	2.44278063061771	-0.80606916428593
H	-3.45055932967215	1.42160955851089	-0.82778392410035
H	-3.43945594819395	2.99570238712646	-1.67382993973896
H	-3.37767817114588	2.94821809565605	0.11084545348304
C	-1.32520254493712	0.67258734413541	-2.83799764997036
H	-2.31406542830714	0.28305070626910	-2.53680224521132
H	-0.65353640433836	-0.18547183758994	-3.03164660718143
H	-1.45095657510335	1.21886734989208	-3.79740107086939
C	1.74968513573626	1.33006522982887	-2.44778672456810
H	1.57699942319981	0.28475433833116	-2.76563516769242
H	2.72831833171416	1.37010152603046	-1.93732346397880
H	1.81732821040210	1.95525419740809	-3.36323037832664
C	2.00695586046848	3.40728145942260	0.00532004205539
H	2.88697697875957	2.75110494854636	-0.12487986886374
H	1.94051299271738	3.69441068733873	1.07164810165891
H	2.20593260293393	4.33842981587078	-0.56738774174649

C	-0.91231817835123	4.20491392486197	0.91969294381555
H	-1.96843558830683	4.17274971649752	1.24545708550967
H	-0.73715959271870	5.20558612150660	0.46919841943113
H	-0.26636004852342	4.14359866486986	1.81803074353894
H	0.82297848867153	-3.74471587120286	-0.54341233830798
H	-1.23664350917320	-3.57997822737376	-0.22399367631356
C	0.41439429249054	1.06485650727562	2.12365030586054
C	-0.98442656278335	1.20254129729343	2.12486671711000
H	1.07261935089772	1.93923845457095	2.24101715492404
H	0.82892033737291	0.10132092340238	2.45368167649866
H	-1.45716592410948	2.19040468168482	2.21947964745286
C	-1.85412254060695	0.02496344007504	2.48037069851945
O	-1.37060704751878	-1.07980437665278	2.70918724677089
C	-3.33792969421618	0.30439001280982	2.54187611337088
H	-3.90336472616835	-0.61571537547949	2.77228778102867
H	-3.67543456772841	0.72540727148441	1.57260239771372
H	-3.55344651753521	1.06960957100542	3.31754951219962

TS C-C

C	-3.21114877626006	-2.78748751248544	0.00042822747153
Co	-0.34794998375009	1.05878082455097	0.11250709802759
N	-1.73887773996737	-2.72183898260764	-0.03921963370173
C	-1.02402892318579	-1.59803363048403	-0.04490167587204
O	-1.59289264346666	-0.45410429326134	-0.06678386599615
C	0.44251365895525	-1.61180861046802	-0.06398376159447
C	1.06011370752012	-0.39304888578734	0.34448584437302
C	2.45908979779428	-0.30751194723962	0.22704777023891
C	3.20948439662382	-1.36331213066386	-0.28417954549131
C	2.58869080787868	-2.55410800791642	-0.67903719055888
C	1.20373058338351	-2.67090806354883	-0.56898928466577
H	2.97351516963164	0.58930653440749	0.57694289256746
H	4.29249636764025	-1.26091533701434	-0.36758970886496
H	3.17865044319017	-3.37808611884276	-1.07859591781849
H	-3.54489399806893	-1.74496983971454	-0.08250730800098
C	-3.66571086161188	-3.36188134427707	1.34303108705064
C	-3.72027669130316	-3.59149145063911	-1.19681515376153
H	-3.28525775155830	-2.75378279755707	2.17344089178947
H	-3.30973068868174	-4.39631326650536	1.46739726554376
H	-4.76319914704132	-3.38160413966418	1.38691586497369
H	-3.36050335637038	-4.63060883848386	-1.15497265111615
H	-3.38986176099975	-3.14945305708334	-2.14660649374242
H	-4.81813058243609	-3.62017021686541	-1.19059031122011
C	-0.75386603988425	3.09223588601043	-0.10716512685803
C	0.62791659019555	2.80894988670488	-0.40566192764432
C	0.66733363981273	1.91338278148167	-1.54512181469337
C	-0.66917995707229	1.57087390016645	-1.87638003615629
C	-1.56155062824778	2.29319980700163	-0.97564004345583
C	-3.04870838507489	2.22317075901065	-1.01761126715226
H	-3.39008885682298	1.17951419366154	-0.98552995938680
H	-3.42745070557352	2.66733831922072	-1.95172434011556
H	-3.50589202972752	2.76527996362668	-0.18227391707137
C	-1.10251627108371	0.64240802379994	-2.95754973539710
H	-2.03752643578005	0.13177379077110	-2.69641914250449
H	-0.33804093762443	-0.11591531226877	-3.16640108613636
H	-1.27798921292912	1.20224344749189	-3.89012728777385
C	1.88395527655813	1.47997953395285	-2.28728032592709
H	1.81694842583586	0.43439778578828	-2.61167859123552
H	2.79442681867214	1.58967567213252	-1.68784427550818
H	2.00099174849550	2.10325694968064	-3.18704142917160
C	1.80671744488510	3.44816065544453	0.24789772025730
H	2.70112148022701	2.81747873801059	0.17843798590252

H	1.61910128043855	3.66726346991854	1.30656280428855
H	2.04388836997025	4.40450030294435	-0.24487832751426
C	-1.24268247420245	4.09846698730456	0.87892052805737
H	-2.23572093520446	3.84708314548179	1.27009505248961
H	-1.31896574290616	5.08128232033196	0.38742992260988
H	-0.55513073748041	4.21677263676102	1.72585129554597
H	0.71316242105065	-3.57885026895232	-0.92482879070136
H	-1.23140273706499	-3.59531442951863	0.08069217098917
C	0.57437680565936	0.39121234359344	1.92896405252918
C	-0.69301262597879	1.09500450080854	2.08117165237294
H	1.46888605500254	0.96816966222662	2.16621785936988
H	0.54546170457691	-0.58296963241371	2.42881530812977
H	-0.67711118890202	2.13907969870455	2.39608906016395
C	-1.85421799786622	0.29674835182693	2.56064015040089
O	-1.75355875577883	-0.91355067710013	2.75621808612587
C	-3.15409429627397	1.03217034120536	2.77516459858838
H	-3.84879911129705	0.41645658696667	3.35645639781138
H	-3.60372475105224	1.24103007443802	1.79156568958059
H	-2.99633427546846	1.99849291593540	3.27481364955932

RS 2 acetanilide

C	-1.54569542945636	3.22289572244975	0.50576947895965
C	-2.96338239611228	3.43941118739389	0.71203271721986
C	-3.21788587757037	3.48186432113638	2.15573474757824
C	-2.00372192960482	3.17256476794825	2.81206484995643
C	-0.96113378948308	2.97799489128760	1.79634493099956
C	0.47100093257856	2.69728401459614	2.11823073153980
H	0.91788175055443	3.54821338977619	2.67584035177444
H	1.08014144696736	2.54278146978845	1.20877247559217
C	-0.84039724015067	3.33799391287227	-0.80656606377132
H	-0.67504820524846	4.41170554508535	-1.03797543401574
H	-1.43014051655301	2.89959159099240	-1.63311397317237
C	-3.97011501913654	3.72807042070691	-0.35428204976727
H	-4.99697112942256	3.48649220547338	-0.02052162276808
H	-3.75866855591439	3.15923814144539	-1.27992436747998
H	-3.95748479271414	4.80775722740754	-0.61855921997432
C	-4.56361842166936	3.69151041390557	2.76857785194934
H	-4.50701135639657	3.72814490199205	3.87257016320933
H	-5.24709407935490	2.86136156292495	2.49018635098152
H	-5.01537098374340	4.64157663520134	2.41733563033764
C	-1.76549972955746	3.01360232820497	4.27739829990820
H	-1.03365860658829	3.76791957496090	4.63529915473941
H	-1.33926839024588	2.01432452708252	4.49852914436660
H	-2.69292443598113	3.13690751468604	4.86661270986443
Co	-2.45870301719509	1.62389792511224	1.40236311123087
O	-4.26624241623584	0.94664945367758	1.35773997906261
C	-4.87355081984338	0.38124270993833	0.40864571174199
N	-4.23535698184041	-0.13636350581816	-0.65904796926277
C	-6.37262154734632	0.27837507721582	0.47327289256480
H	-6.80318580844904	-0.35550553987577	-0.32423996062635
H	-6.81443714686507	1.29311811105009	0.40625635739852
H	-6.65203535392136	-0.13246271308534	1.46345087494783
H	-4.81689246189630	-0.58460507940604	-1.37087599404128
C	-2.83076367791720	-0.22433512552896	-0.85293103260609
C	-2.39759982175599	-1.06354540349617	-1.90537847601030
C	-1.03207561016042	-1.25583139760396	-2.13627233801194
C	-0.09902245118312	-0.61776774339893	-1.30097865837620
C	-0.54610757678638	0.22379976047359	-0.26924405169754
C	-1.91586979219817	0.46720881757579	-0.02224102929494
H	-3.14185348603843	-1.57452012635381	-2.53860079377192

H	-0.69910035097149	-1.90608962550692	-2.95853453324210
H	0.98004054977402	-0.77215292931767	-1.45477887543653
H	0.20431108863035	0.70582987993618	0.37373228299236
H	0.14514665464971	2.84036279916512	-0.79838476462529
H	0.56485906798132	1.79829556238369	2.75989523531536
H	-3.50812337212618	0.80769805046595	4.34097927550811
O	-1.83275664841107	0.23838668292973	2.78925124521532
C	-1.99029317194433	-1.00827093589544	2.78275970110070
N	-3.16001981968729	-1.61020905000183	3.12047912539054
C	-0.85579959776677	-1.92255562727207	2.39949317377865
H	-1.05326519418899	-2.99424872056636	2.59096875985776
H	0.04816759313603	-1.61242460371439	2.95800147448712
H	-0.63573540901052	-1.76694334555297	1.32284806740056
H	-3.16864696437131	-2.63018749913345	3.03406096029478
C	-4.36961682602665	-1.04642705642618	3.60547677204141
C	-5.54290052545160	-1.81734848014445	3.47117501730921
C	-6.76464897255735	-1.32783893680469	3.95584624855199
C	-6.82337521716843	-0.06779488108593	4.57438527896489
C	-5.64781572309926	0.69022511253110	4.71424399311860
C	-4.42055473594722	0.21272282450226	4.23654832483687
H	-5.49713435105407	-2.80600416366903	2.98778164053683
H	-7.67347069878697	-1.93965009006496	3.85450344157238
H	-7.77938847295179	0.31517988830659	4.95978343206684
H	-5.68479717821428	1.66992065714119	5.21249924168878

RS 2 benzamide

C	-0.26166619732530	-1.76783785420156	-3.68763613754834
Co	0.45104054026255	-0.73469418943375	0.92426237526285
N	-0.66921320199070	-2.54066293736084	-2.50326057667739
C	-0.40827620671063	-2.18321249335808	-1.24133500486763
O	0.24740643225905	-1.11519200841566	-0.99808745684223
C	-0.82494362469251	-2.98925448396496	-0.09258224157801
C	-1.41272599948213	-4.26236825514064	-0.15569957639010
C	-1.67964422949741	-4.95421248618724	1.02160582342713
C	-1.34840742620869	-4.37225061319995	2.25319799277728
C	-0.76129706756455	-3.10447185262653	2.31122696365190
C	-0.49655950569827	-2.38011835263541	1.14333369947638
H	-1.64537672003483	-4.73166061639245	-1.11509569749836
H	-2.13851547942675	-5.94181866078638	0.98682842189120
H	-1.55319070248929	-4.91574486178968	3.17749890313267
H	-0.50663097052941	-2.68722477335177	3.28662236041658
H	0.74712685569601	-1.39092788627662	-3.46366987323586
C	-1.20089754892149	-0.58249419359063	-3.91908015658290
C	-0.20593726438862	-2.70796105852768	-4.88686917862311
H	-1.23526403873038	0.06613382944768	-3.03490893534764
H	-2.22158572680687	-0.92729633034669	-4.13804384690494
H	-0.85158292523261	0.01671053979984	-4.77148712943167
H	-1.20519953133082	-3.10642253141546	-5.12178924720808
H	0.47326425929200	-3.55020368743304	-4.70136661127010
H	0.14816081340666	-2.16874653524686	-5.77459946492401
C	-0.81046475243117	0.84160638919921	0.88190708895750
C	-0.76206530918950	0.29967861642900	2.21412512758208
C	0.60125723776789	0.35891595977788	2.65110850205654
C	1.37785333363668	1.04464586617879	1.62125984664882
C	0.51718864224857	1.34590186102611	0.54541647140209
C	0.86683312430748	1.97814948330691	-0.75854437162475
H	1.94376250692050	2.16044735484657	-0.84693916400618
H	0.34899724171455	2.94223071793144	-0.87325599687807
H	0.56009325031130	1.33300059470625	-1.59463365418290
C	2.84473033021689	1.29149089977067	1.71219639371705

H	3.25413123652877	1.67324539848952	0.76978600206440
H	3.37958376039178	0.36641519638119	1.96759511268478
H	3.06246745493128	2.02961558478356	2.49978053330359
C	1.14675967080989	-0.09465165362257	3.96200758613449
H	2.13086820250439	-0.56554013291080	3.83775287060669
H	0.47754301863244	-0.81428601891467	4.45211258754390
H	1.27223240532140	0.75871796561428	4.64780675282131
C	-1.94199251627272	-0.14986021182715	3.00622252288667
H	-1.66006084824957	-0.81642404195584	3.82856198457597
H	-2.67813084323470	-0.67132795064108	2.38275438218693
H	-2.43574575828761	0.72963147644571	3.44914253734444
C	-2.02555840697776	0.96938278094486	0.02974160729543
H	-1.76810402452195	0.93508717118528	-1.03585242384323
H	-2.53049608455222	1.93202342854843	0.21704839175599
H	-2.74830760549125	0.17069891268248	0.23853044258747
H	-1.22061327865633	-3.38087330322677	-2.65059371813680
C	2.71097233088943	-3.62180553741765	3.28429549779260
H	3.18120061869236	-1.40868633153959	-1.14452148536770
N	2.78312391713958	-3.84087816576966	1.83329262285784
C	2.44606468777869	-2.92247330102191	0.91680471521635
O	2.15539137401468	-1.74489796178431	1.27880002533706
C	2.49222914615750	-3.34444411619712	-0.50340532170054
C	2.14092336145533	-4.64575658940388	-0.89644480497844
C	2.25212535793599	-5.02397136221699	-2.23365090302870
C	2.72696697007695	-4.11019987844655	-3.17948663900500
C	3.05983649151815	-2.80749616625364	-2.79417006643906
C	2.93182968991753	-2.42029602958278	-1.46192314963377
H	1.74086561525845	-5.34690853737935	-0.16204557664032
H	1.97212609242805	-6.03228232066644	-2.53895158582141
H	2.84131478423913	-4.41574114152092	-4.22002037548792
H	3.42991733052051	-2.09682202213733	-3.53358777663409
H	1.90705897644561	-2.88524440794132	3.42238851431448
C	4.02336994930198	-3.03623293026632	3.81303945068992
C	2.32713534724075	-4.93308491598989	3.96448813825093
H	4.27498826564602	-2.10445420304964	3.28900055964505
H	4.85315595922572	-3.74525098720168	3.67606044843146
H	3.93587618001793	-2.81869731754281	4.88621191527726
H	3.09793039679649	-5.70400956095019	3.80770773464916
H	1.36969951895724	-5.31029746331002	3.58088941560514
H	2.22994444192135	-4.78086455265004	5.04747462005268
H	3.16859267419054	-4.72179925247485	1.50071320602710

[6] References:

[1] Sun, B.; Yoshino, T.; Matsunaga, S.; Kanai, M. *Adv. Synth. Catal*. **2014**, *356*, 1491-1495.

[2] Chirila, P. G.; Adams, J.; Dirjal, A.; Hamilton, A.; Whiteoak, C. J. *Chem. Eur. J.* **2018**, *24*, 3584-3589.

[3] Neese, F. *WIREs Comput. Mol. Sci*. **2012**, *2*, 73–78.

[4] Neese, F. *J. Comp. Chem*. **2003**, *24*, 1740-1747.

[5] (a) Becke, A. D. *Phys. Rev. A* **1988**, *38*, 3098-3100. (b) Perdew, J. P. *Phys. Rev. B* **1986**, *33*, 8822-8824. (c) Perdew, J. P. *Phys. Rev. B* **1986**, *34*, 7406.

[6] Grimme, S.; Enrlich, S.; Goerigk, L. *J. Comput. Chem*. **2011**, *32*, 1456-1465.

[7] (a) Weigend, F.; Ahlrichs, R. *Phys. Chem. Chem. Phys.* **2005**, *7*, 3297-3305. (b) Schäfer, A.; Horn, H.; Ahlrichs, R. *J. Chem. Phys*. **1992**, *97*, 2571-2577.

[8] Neese, F.; Wennmohs, F.; Hanson, A.; Becker, U. *Chem. Phys*. **2009**, *356*, 98-109.

[9] Zhao, Y.; Truhlar, D. *Theor. Chem. Account* **2006**, *120*, 215-241.

[10] Sinnecker, S.; Rajendran, A.; Klamt, A.; Diedenhofen, M.; Neese, F. J. Phys. Chem. A **2006**, *110*, 2235-2245.

[11] Allouche, A.-R. *J. Comp. Chem*. **2011**, *32*, 174-182.

[12] Hanwell, M.; Curtis, D. E.; Lonie, D. C.; Vandermeersch, T.; Zurek, E.; Hutchison, G. R. *J. Cheminform*. **2012**, *4*, 1-17.
